# Genetic Variation and Antioxidant Response Gene Expression in the Bronchial Airway Epithelium of Smokers at Risk for Lung Cancer

**DOI:** 10.1371/journal.pone.0011934

**Published:** 2010-08-03

**Authors:** Xuting Wang, Brian N. Chorley, Gary S. Pittman, Steven R. Kleeberger, John Brothers, Gang Liu, Avrum Spira, Douglas A. Bell

**Affiliations:** 1 Environmental Genomics Section, Laboratory of Molecular Genetics, National Institute of Environmental Health Sciences, National Institutes of Health, Research Triangle Park, North Carolina, United States of America; 2 Environmental Genetics Section, Laboratory of Respiratory Biology, National Institute of Environmental Health Sciences, National Institutes of Health, Research Triangle Park, North Carolina, United States of America; 3 Section of Computational Biomedicine, Department of Medicine and the Pulmonary Center, Boston University, Boston, Massachusetts, United States of America; Helmholtz Zentrum München/Ludwig-Maximilians-University Munich, Germany

## Abstract

Prior microarray studies of smokers at high risk for lung cancer have demonstrated that heterogeneity in bronchial airway epithelial cell gene expression response to smoking can serve as an early diagnostic biomarker for lung cancer. As a first step in applying functional genomic analysis to population studies, we have examined the relationship between gene expression variation and genetic variation in a central molecular pathway (NRF2-mediated antioxidant response) associated with smoking exposure and lung cancer. We assessed global gene expression in histologically normal airway epithelial cells obtained at bronchoscopy from smokers who developed lung cancer (SC, n = 20), smokers without lung cancer (SNC, n = 24), and never smokers (NS, n = 8). Functional enrichment analysis showed that the NRF2-mediated, antioxidant response element (ARE)-regulated genes, were significantly lower in SC, when compared with expression levels in SNC. Importantly, we found that the expression of MAFG (a binding partner of NRF2) was correlated with the expression of ARE genes, suggesting MAFG levels may limit target gene induction. Bioinformatically we identified single nucleotide polymorphisms (SNPs) in putative ARE genes and to test the impact of genetic variation, we genotyped these putative regulatory SNPs and other tag SNPs in selected NRF2 pathway genes. Sequencing MAFG locus, we identified 30 novel SNPs and two were associated with either gene expression or lung cancer status among smokers. This work demonstrates an analysis approach that integrates bioinformatics pathway and transcription factor binding site analysis with genotype, gene expression and disease status to identify SNPs that may be associated with individual differences in gene expression and/or cancer status in smokers. These polymorphisms might ultimately contribute to lung cancer risk via their effect on the airway gene expression response to tobacco-smoke exposure.

## Introduction

Approximately 1.3 billion people smoke cigarettes worldwide, contributing to almost 5 million preventable deaths per year [1]. Smoking is a significant risk factor for lung cancer, the leading cause of cancer-related death in the United States, and chronic obstructive pulmonary disease, the fourth leading cause of death overall [2]. Even with the high attributable risks due to cigarette smoke exposure, only 10–15% of all smokers develop lung cancer [3], suggesting genetic variability may play a role in susceptibility to lung cancer. Lack of knowledge of the genetic basis of lung cancer prevents accurate prediction of smokers with the highest risk. However, rapid advances in high-throughput genomics techniques, especially gene expression profiling and single nucleotide polymorphism (SNP) genotyping, show promise for characterizing risk. Understanding how genetic variation influences smoking-induced gene expression in the lung and airway could reveal susceptibility factors.

Previous studies have demonstrated that cigarette smoke exposure creates a “field of injury” in airway epithelial cells (reviewed in [4]). Spira *et al* [5] have measured whole-genome gene expression profiles in epithelial cell brushings collected at bronchoscopy from the mainstem bronchus of healthy smokers and never smokers,. Smoking-induced gene expression was observed for genes involved in regulation of oxidant stress, xenobiotic metabolism, and oncogenesis, while genes involved in inflammation and tumor suppression pathways were down regulated. Recently, using a similar approach, an 80-gene biomarker was developed to help diagnose individuals with lung cancer among a group of smokers having a bronchoscopy due to suspicion of lung cancer [6], [7]. Profiles of histologically normal large-airway epithelial cells obtained at bronchoscopy were effectively used as an early diagnostic lung cancer biomarker, with an accuracy of 83%. These observations reveal airway gene-expression differences among individuals in response to smoking, but do not point to the molecular mechanisms that contribute to the heterogeneity in this gene-expression response.

Human genetic variability in the response to environmental exposure is generally accepted as an important determinant in susceptibility to cancer [8]. However, a challenging problem in association studies is interpreting if statistical evidence of genotype–phenotype/disease correlation is biologically plausible. Often the relationship between specific single nucleotide polymorphisms (SNPs) and gene expression or activity has been difficult to study *in vivo*. Examining SNPs in transcription factor pathways and specifically, in transcription factor binding sites, in relationship to gene expression is an approach that can yield functional information [9]. Recently, *cis*-acting regulatory SNPs have been discovered using a regional association approach [10], [11]. To better understand these functional relationships that contribute to *in vivo* differences in gene expression and possibly to lung cancer susceptibility, we have used a three-part approach to test associations between: (1) gene expression and lung cancer status, (2) SNP genotype and gene expression, and (3) SNP genotype and lung cancer status.

Using the approach described by Spira *et al* [7], we assessed global gene expression in cytologically normal airway epithelial cells obtained by bronchoscopy from smokers with suspicion of lung cancer and from a control group of never smokers. We identified that the antioxidant response pathway regulated by the transcription factor NRF2 (nuclear factor erythroid-derived 2-like 2, or NFE2L2) differed among these groups of subjects. We found that the expression of MAFG (a binding partner of NRF2) was correlated with the expression of NRF2 pathway genes. We used bioinformatics strategies to identify putative regulatory SNPs in NRF2 binding sites [9], [12], [13] and to select tag SNPs for NRF2-mediated genes. Additionally, we sequenced the MAFG locus in our study subjects. Gene expression, genotype and lung cancer status was integrated and compared, and we identified SNPs that were associated with individual differences in gene expression and/or cancer status.

## Results

### Cigarette smoking, lung cancer and the bronchial airway transcriptome

**Figure 1:**
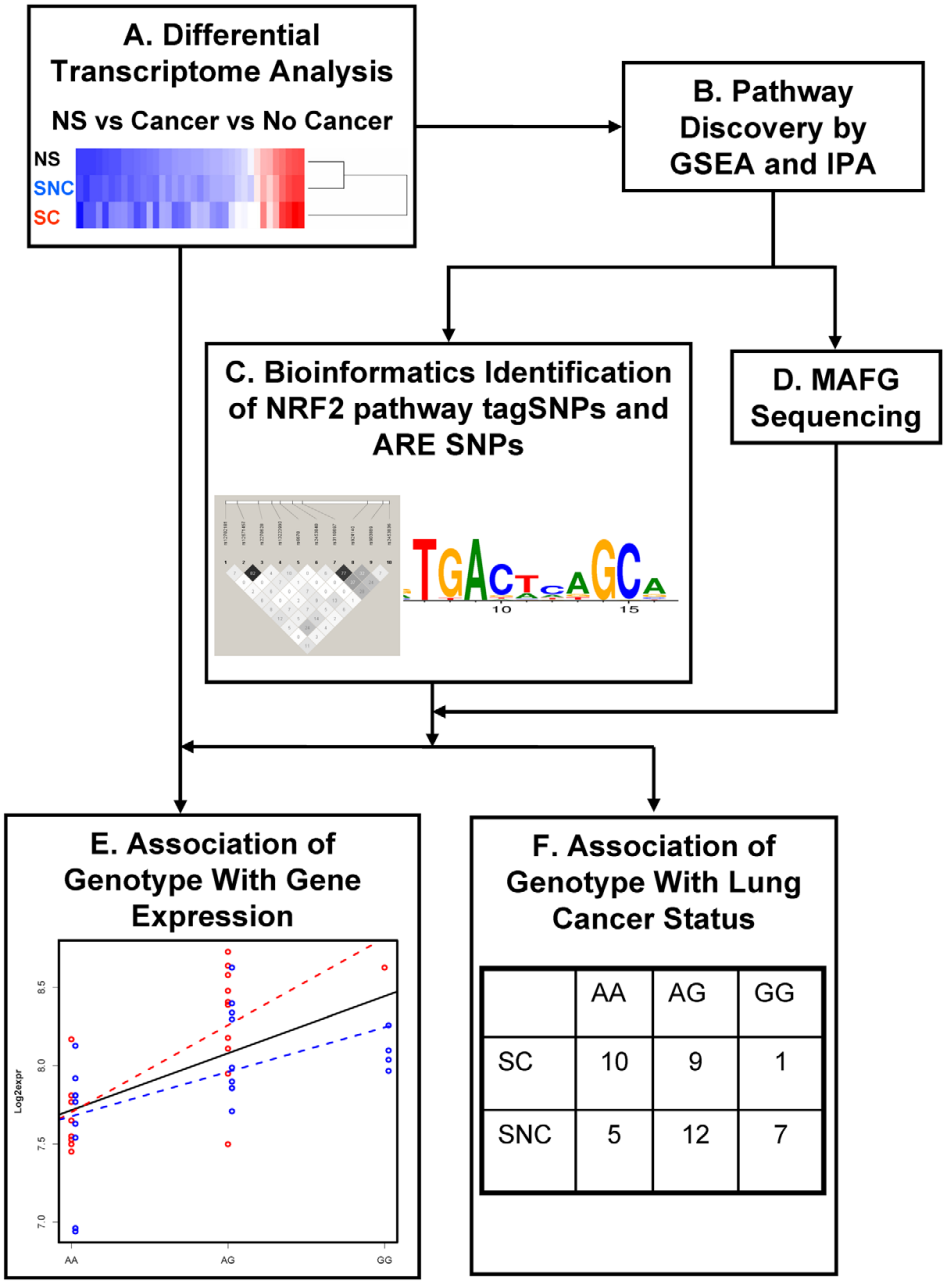
The workflow for examining the relationship between gene expression variation and genetic variation in the NRF2-mediated antioxidant response pathway associated with smoking exposure and lung cancer. (A) We assessed microarray gene expression profiles of histologically normal airway epithelial cells obtained by bronchoscopy from smokers with suspicion of lung cancer and from a control group of never smokers. (B) We identified that the antioxidant response pathway regulated by the transcription factor NRF2 differed among these groups of subjects. We found that the expression of MAFG (a binding partner of NRF2) was correlated with the expression of NRF2 pathway genes. (C) Bioinformatics strategies were used to identify putative regulatory SNPs in NRF2 binding sites and to select tagging SNPs for NRF2-mediated genes. (D) The MAFG locus was sequenced in our study subjects. We identified SNPs that were associated with individual differences in: (E) gene expression and/or (F) cancer status by integrating gene expression, genotype and lung cancer status.

With the aim of identifying potential functional SNPs and/or haplotypes in antioxidant response pathways associated with smoking-induced lung cancer, we used a three-part approach to analyze the associations between: 1) gene expression and lung cancer status; 2) gene expression and genotype of SNPs selected by several bioinformatics strategies, and 3) SNP genotypes and lung cancer status. The overall workflow of our approach is outlined in Figure 1.

Bronchial airway epithelial cells were obtained by flexible bronchoscopy from 8 healthy never-smokers (NS) and from smokers enrolled in a diagnostic study for clinical suspicion of lung cancer including 20 smokers with lung cancer (SC), and 24 smokers without lung cancer (SNC) (Table 1). These subjects were a subset two larger gene expression projects [5], [7] for whom we could obtain sufficient genomic DNA for genotyping. Expression data for 31 subjects from these projects [5], [7] and data for an additional 21 newly recruited patients were used. Using Affymetrix HG-U133A microarrays, we found 11285 probe-sets expressed at measurable levels (detection p-value < = 0.05 in at least 20% of individuals in either of SC, SNC, or NS), which corresponded to 8159 protein-coding RefSeq genes based on Affymetrix annotation (HG-U133A.na28.annot.csv, March 2009).

**Table 1:** Demographic characteristics of the study population.

Parameters	Never smokers (n = 8)	Smokers without cancer (n = 24)	Smokers with cancer (n = 20)	Difference between Cancer/No Cancer
Age(years)	32±9	53±18	68±15	p<0.05 by t-test
Smoking history(packs/year)	0	52±53	58±28	p = 0.63 by t-test
Gender(female:male)	3 ∶ 5	2 ∶ 22	4 ∶ 16	p = 0.13 by Fisher's exact test
Race (AFA:ASI:CAU:HIS:OTH)*	0∶0∶6∶1∶1	6∶3∶13∶1∶1	2∶0∶18∶0∶0	p = 0.07 by Fisher's exact test

* AFA =  African American; ASI =  Asian; CAU =  Caucasian; HIS =  Hispanic; OTH =  Other.

To examine the differential responses to the effect of cigarette smoking on the bronchial airway transcriptome, we used profiles from never-smokers as baseline and compared the average log2-expression values of SC or SNC with that of NS by t-test, followed by multiple testing correction (Benjamini-Hochberg false discovery rate, FDR [14]). Compared with never-smokers, we found differential expression (FDR 0.1) for 846 probe-sets (774 genes) in smokers without cancer, and 919 probe-sets (834 genes) in smokers with cancer. We next classified smoking-dependent genes into over-expressed (fold change >1.2) and under-expressed (fold change <0.8) genes. In smokers without cancer, there were 210 probe-sets over-expressed and 628 probe-sets under-expressed; in smokers with cancer, there were 263 probe-sets over-expressed and 644 probe-sets under-expressed, compared with never-smokers. The list of probe-sets, average log_2_-expression values, and statistical values are included in the Supporting Information S1.

### Functional enrichment analysis of smoking affected gene expression signatures

To identify sets of related genes with common biological function, we analyzed expression profiles using Gene Set Enrichment Analysis (GSEA) [15], Ingenuity pathway analysis (IPA), and Gene Ontology enrichment analysis (GOEA). GSEA tested whether any *a priori* defined canonical pathways were enriched among differentially expressed genes in the groups. We were particularly interested in differences between the cancer and no cancer groups. We found that two gene sets were enriched in SNC versus NS, at the FDR 0.1 level. The first gene set was the “antioxidant response element genes” (curated and published previously [9]), with a normalized enrichment score (NES) of 2.04 and a FDR q-value of 0.021; the second gene set was the “actin Y pathway” (NES = 1.87, q-value = 0.077). Two gene sets were enriched in NS, including “Histidine metabolism” (NES = 1.93, q-value = 0.082) and “RAR/RXR pathway” (NES = 1.84, q-value = 0.093). No gene set was enriched at FDR 0.1 or 0.25 level in SC compared to NS. We also observed no enriched gene set in comparing SNC versus SC at the FDR 0.1 level, however we found 4 gene sets enriched at the FDR 0.25 level, the most significant gene set was “antioxidant response element genes” (NES = 1.86, q-value = 0.120). These results suggested that the set of “antioxidant response element genes” were significantly enriched in SNC but not in SC.

**Figure 2:**
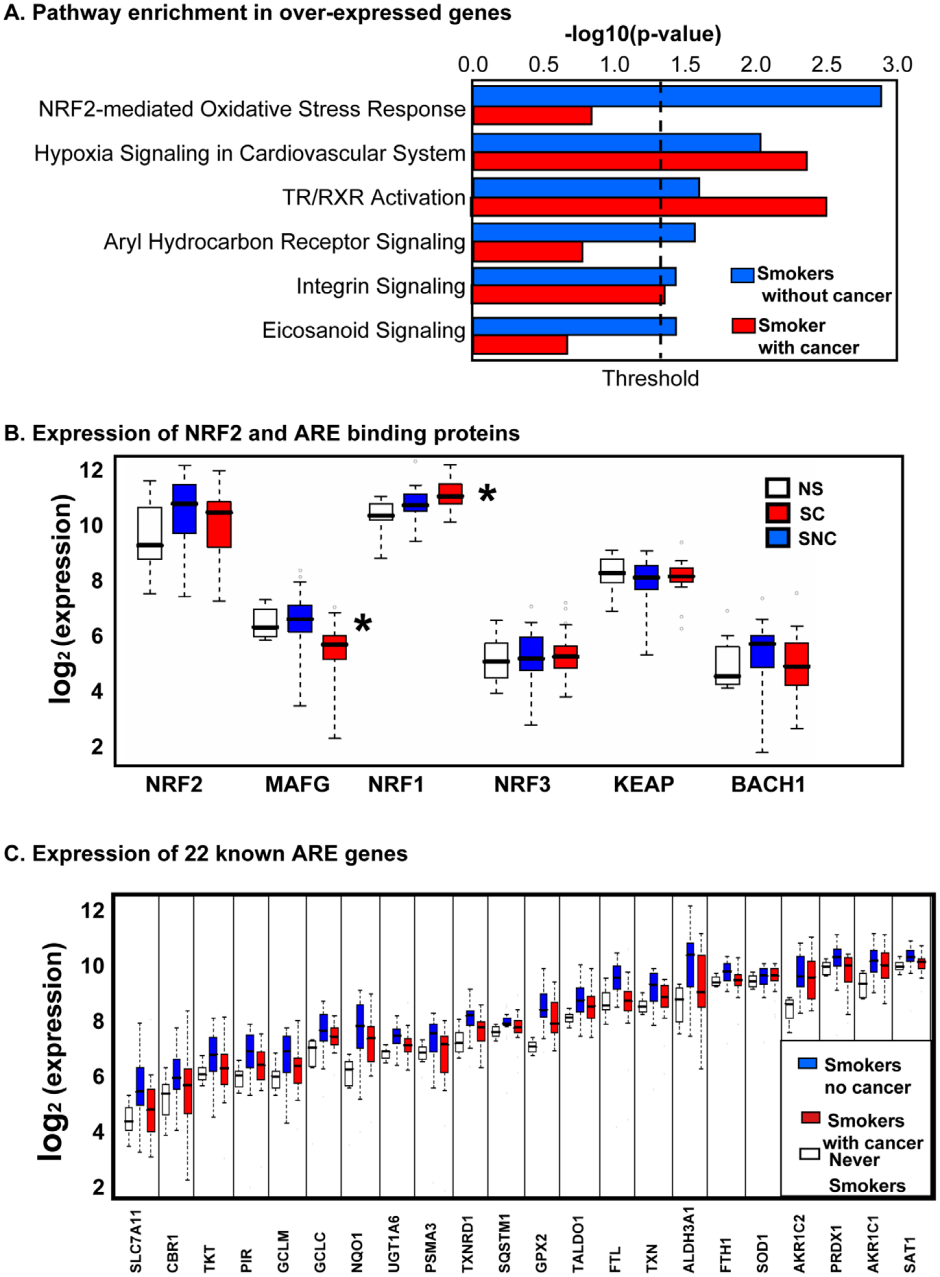
Pathway analysis and NRF2 pathway gene expression. (A) Ingenuity Pathway Analysis revealed differential effects of cigarette smoking in smokers without cancer (SNC) and smokers with cancer (SC) relative to nonsmokers (NS). Six canonical pathways were significant enriched in the 210 over-expressed probe-sets when comparing SNC with NS (blue bars). Three pathways were significantly enriched in the 263 over-expressed probe-sets when comparing SC versus NS (red bars). We observed a very pronounced difference in the significance level of “NRF2-mediated oxidative stress pathway” between SNC and SC. (B) The mRNA levels of the interacting, regulatory components of the NRF2 pathway displayed as boxplots. A boxplot depicts a dataset through five-number summaries: the smallest observation, lower quartile, median, upper quartile, and largest observation. Compared with NS, the master regulator NRF2 showed no difference between SNC and SC. However, the binding partner MAFG was significantly lower in SC, and the competitor NRF1 was significantly higher in SC. NRF3, KEAP1, and BACH1 mRNA showed no significant changes. (C) We observed that 22 genes with known NRF2 binding sites showed significant differences among the groups at FDR 0.1 level. Consistently, the expression of these genes in SNC was higher than that in NS; and most of these genes have lower expression in SC than that in SNC. This pattern was similar to MAFG expression pattern.

We also used IPA to test for enriched canonical pathways using lists of over-expressed or under-expressed probe-sets and determining the relative weight of identified pathways in the different phenotype groups. For the 210 over-expressed probe-sets between SNC and NS, six pathways were significant (Figure 2A), including “NRF2-mediated oxidative stress response”, “hypoxia signaling in cardiovascular system”, “TR/RXR activation”, “aryl hydrocarbon receptor signaling”, “integrin signaling”, and “eicosanoid signaling”. GSEA and IPA represent two distinct functional enrichment methods that are based on independent knowledge databases and different statistical tests. Both identified the NRF2 pathway as the most enriched pathway, demonstrating consistency between these methods. Most importantly, using IPA, for over-expressed genes we observed a very pronounced difference in the significance level of “NRF2-mediated oxidative stress pathway” between SNC and SC (Figure 2A). Furthermore, Gene Ontology analysis also supported the role of oxidative stress genes among over-expressed genes in SNC. There were 15 GO terms enriched from over-expressed genes at FDR 0.1 level in SNC and all of them were molecular function terms for antioxidant response genes (Supporting Information S1); however, no GO terms were enriched among over-expressed genes in SC. Thus the three bioinformatic analyses support a role for NRF2 pathway genes in the group without cancer but not in the lung cancer group.

### Correlation of MAFG with the expression of antioxidant response genes

Functional enrichment methods strongly implicated the significance of NRF2 pathway (defined by genes containing antioxidant response elements (AREs) in the upstream regions). As a result, we examined this group of genes more closely. Under oxidative or electrophilic stress, NRF2 is released from its interaction with KEAP1 and translocates to the nucleus, where it heterodimerizes with small MAF proteins such as MAFG, and then binds ARE sequences upstream of NRF2 target genes [16]. NRF1, NRF3, and BACH1 can compete with NRF2 to bind with MAFG at ARE sites, potentially leading to decreased ARE-mediated gene expression [17], [18], [19]. Figure 2B displays the mRNA levels of the interacting regulatory protein components of the NRF2 pathway. Compared with NS, we found no significant difference in the master regulator NRF2 between either SNC or SC. However, we found significant difference in the competitor NRF1 and the binding partner MAFG that are consistent with a role in regulation of the downstream target genes (Figure 2B). NRF1 was significantly higher (fold change  = 1.23, p = 0.0115 for SC versus SNC; and fold change  = 1.54, p = 0.0084 for SC verse NS, by t-test). The change in NRF1 expression in this process supports the role for NRF1 proposed by Wang *et al* [20] and Ohtsuji *et al* [16], who demonstrated that NRF1 binding to AREs *in vivo* repressed ARE-dependent gene expression and modulated response to oxidative stress. That is, NRF1 was observed to be anti-correlated with expression of NRF2 pathway genes. MAFG was lower in SC compared to NS or SNC (fold change  = 0.55, p = 0.0022 for SC versus SNC, and fold change  = 0.60, p = 0.0076 for SC versus NS, by t-test). No significant changes were found in NRF3, KEAP1, and BACH1 mRNA (Figure 2B). As expected based on the IPA and GSEA results, we found that 22 ARE-regulated genes were different between groups, and correlated with MAFG levels (lower in SC but higher in SNC, Figure 2C). NRF2 target genes shown in Figure 2C (AKR1C1, AKR1C2, ALDH3A1, CBR1, FTH1, FTL, GCLM, GCLC, GPX2, NQO1, PIR, PRDX1, PSMA3, SAT1, SLC7A11, SOD1, SQSTM1, TALDO1, TKT, TXN, TXNRD1, and UGT1A6) were induced among SNC but were consistently lower in the SC patients. This novel observation suggests that reduced levels of MAFG and higher levels of NRF1 (negative regulator) may be suppressing the transcription of these ARE-regulated genes among smokers who go on to develop lung cancer.

**Figure 3:**
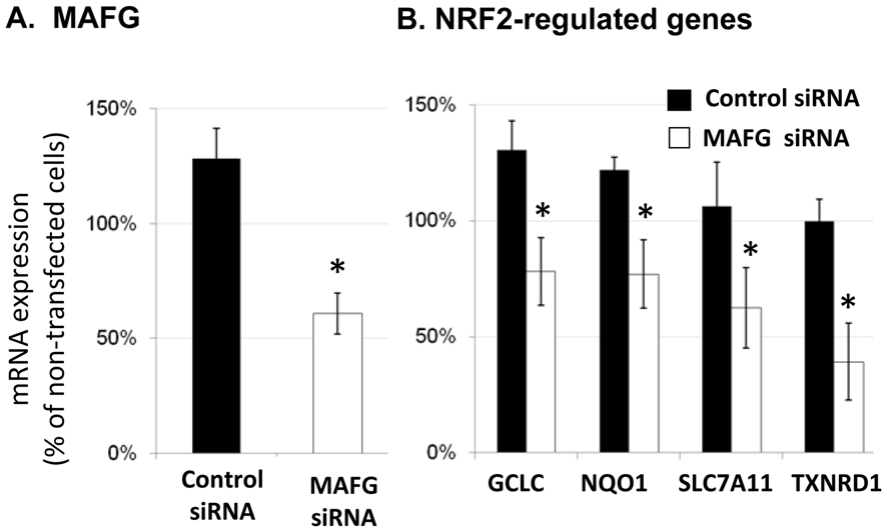
MAFG silencing attenuates downstream antioxidant and Phase II gene expression. Following transient transfection with MAFG siRNA in the A549 airway cell line, gene expression was measured using real-time qPCR. Transfection with scrambled control siRNA produced a general increase in NRF2 pathway genes (black bars) relative to nontransfected cells (set at 100%). (A) MAFG gene expression was significantly reduced (55%) compared to non-specific siRNA control. (B) GCLC, NQO1, SLC7A11, and TXNRD1 gene expression was significantly reduced with MAFG silencing compared to non-specific siRNA controls. * (p≤0.05, t-test). All data presented as mean ± SEM (n = 3).

We re-examined a larger, previously published dataset [7] and also found significantly lower MAFG levels in smokers with lung cancer (n = 90) compared with that in smokers without cancer (n = 97) (Supporting Information S1). Further evidence for a regulatory role for MAFG in airway epithelial cells is emerging. In a related project, cigarette smoke-induced airway expression of MAFG has been observed to be regulated by the microRNA miR-218 [21]. We explored the impact of MAFG expression level on downstream NRF2-pathway genes. Figure 3A displays MAFG siRNA knockdown in the A549 cell line. Figure 3B shows that MAFG silencing leads to attenuated expression of GCLC, NQO1, SLC7A11, TXNRD1 (all ARE genes). We hypothesize that reduced MAFG levels and the subsequent reduction in the protective oxidative stress response may represent a gene expression hallmark in bronchial epithelial cells of smokers who develop lung cancer. However, the pattern of gene expression over time (duration of smoking) could be important and recent smoking among the current smokers might influence some of the patterns that we observe in the bronchial epithelial cells.

### SNP selection, genotyping and MAFG sequencing

In order to examine if genetic variation was contributing to airway gene expression differences, we used a bioinformatics strategy to identify SNPs in potential NRF2 binding sites [9], [13] and also identify tagging SNPs for several genes involved in the NRF2-mediated anti-oxidant response pathway. The gene and SNP list is included in the Supporting Information S1. After genotyping, about 77% (348 SNPs) of the identified SNPs passed the initial quality control criteria (genotyping rate  = 90%, MAF threshold  = 0.01 and Hardy-Weinberg equilibrium p-value threshold  = 0.001, GenTrain score 0.25) but we only examined 312 SNPs with allele frequencies ≥0.05 in the 52 subjects.

Expression analysis suggested that MAFG levels could potentially be rate-limiting in the expression of antioxidant response genes. To better evaluate if sequence variability in the MAFG gene affected gene expression, we sequenced a 16.5-kb genomic region (chr17:77,467,438-77,483,879) in our study subjects. This region was from 5000-nt upstream of the transcription start site to 2000-nt downstream of MAFG's transcription end site, covering introns, exons and untranslated regions. We discovered 33 SNPs in this region, including 1 coding SNP, 16 SNPs in 3′ UTR, and 3 SNPs in introns, and 13 SNPs in upstream. The location, allele frequency and linkage disequilibrium (LD) is included in Supporting Information S1. Comparing this information with the NCBI dbSNP build 130 (May 2009), we found that only 3 of these SNPs had been previously reported.

### Association analysis of SNP genotype and gene expression

**Figure 4:**
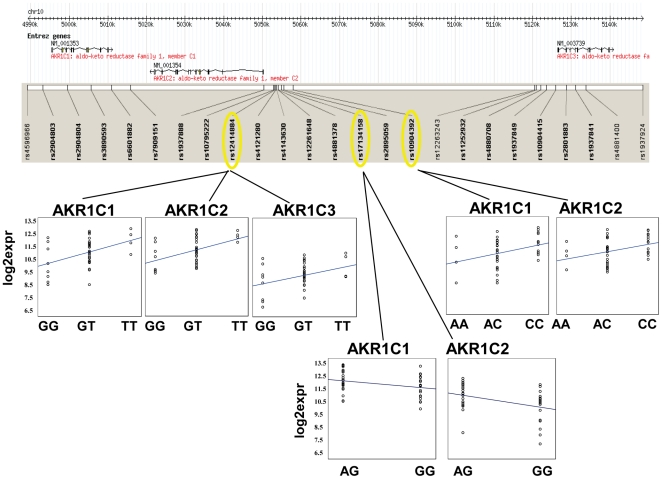
SNPs associated with the expression of 3 known ARE genes, AKR1C1, AKR1C2, and AKR1C3 among smokers. We genotyped 25 SNPs in the genomic region containing AKR1C1, AKR1C2, and AKR1C3. In each plot of expression verse genetype, circles were log2 expression, and lines were linear regression trend lines. The genotypes of SNP rs1241488 associated with the expression levels of all 3 genes. The genotypes of SNP rs1090439 associated with the expression levels of AKR1C1 and AKR1C2. Note: The SNP rs17134158 only had 2 genotypes AG and GG in smokers, but did have AA genotype in NS and its overall minor allele frequency >0.05. Linkage disequilibrium analyses indicated these 3 SNPs were in linkage (r^2^>0.88).

We performed linear regression between normalized log_2_-transformed gene expression values and genotypes of SNPs that were near each gene (SNP position within 10-kb of a gene's upstream, coding, and downstream regions). Statistical significance was evaluated using 10,000 permutations of expression values relative to the genotypes as previously described by Stranger *et al* [22] with a corrected *p* value threshold of 0.05. In 44 smokers, from among 338 detectable probe-sets (213 genes) nearby our selected 312 SNPs, we found significant association between 26 SNPs and 29 probe-sets (25 genes, listed in Table 2). Among these are 21 putative ARE SNPs and 6 known ARE genes, including AKR1C1, AKR1C2, AKR1C3, EPHX1, FTL, and HMOX1. We found a cluster of SNPs associated with expression of 3 adjacent NRF2-regulated genes, AKR1C1, AKR1C2 and AKR1C3 (Figure 4). We genotyped 25 tag SNPs in this genomic region. SNP rs12414884 is located -3792-nt upstream of AKR1C2 and was associated with the expression of all 3 genes. Two SNPs, rs17134158 and rs10904392, which are 1768-nt and 4715-nt away from rs12414884 respectively, were associated with the expression of AKR1C1 and AKR1C2. Further linkage disequilibrium analyses indicated these 3 SNPs were in linkage (r^2^>0.8). We also tested the association between newly identified SNPs in MAFG and MAFG gene expression. The promoter SNP at chr17:77482956 (-4077, A/C, minor allele freq  = 0.09) was associated with MAFG expression (p_corrected  = 0.0038).

**Figure 5:**
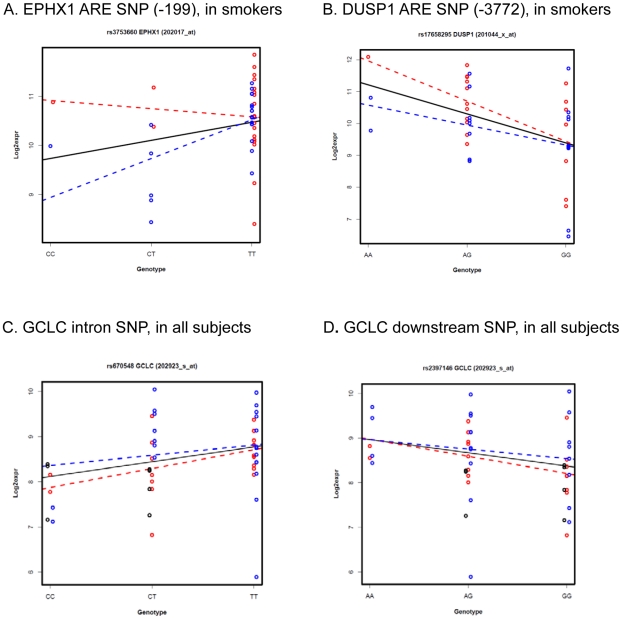
Plots of association between SNP genotype and gene expression for selected SNPs. In each plot, SC, SNC and NS are colored with red, blue, and black, respectively. (A) The association of a putative ARE SNP rs3753660 in the promoter of EPHX1 gene displays distinct trends in SC and SNC; (B) DUSP1 putative ARE SNP rs17658295 associated with its expression. The minor allele was significantly associated with higher expression and the significance level was more pronounced in the cancer group; C) GCLC intronic SNP rs670548 minor allele associated with lower expression among all subjects; (D) GCLC 3′ downstream SNP rs2397146 minor allele associated with higher expression among all subjects.

Although our focus was the association between genotype and expression in all smokers in the study, we also examined the difference in associations between SC and SNC groups. This type of exploratory analysis could reveal SNP effects, that might be related to differential susceptibility in smokers. An association was found between expression and a putative ARE SNP rs3753660 at -199-nt of epoxide hydrolase 1 (EPHX1) gene (Figure 5A). The association was strongest among SNC and the effect of the SNP appears to be quite different between the two groups (Figure 5A and Table 2). Human EPHX1 has two putative AREs (including the polymorphic ARE), but EPHX1 expression did not follow the pattern as displayed by many other ARE genes in Figure 2C. The C allele of rs3753660 was predicted to have lower NRF2 binding and we observed it was associated with lower expression in SNC. This suggests a possible interaction between the genetic variant, expression and the group phenotype. DUSP1 also had a putative ARE SNP rs17658295 associated with its expression (Figure 5B). The minor allele was significantly associated with higher expression and the significance level was more pronounced in the cancer group.

**Table 2:** Association of SNP genotype and gene expression (sorted on SNP class and gene symbol).

SNP	Probe-set	p-value among all 52 subjects (SC+SNC+NS)	p-value among all smokers (SC+ SNC)	p-value in SC	p-value in SNC	SNP location	Symbol	SNP class
***Significant in smokers (SC+SNC) and one sub-group(SC or SNC)***								
rs12414884	204151_x_at		**0.0066**	0.0096		downstream	AKR1C1^ *^	tagSNP
rs12414884	216594_x_at		**0.0051**	0.0176		downstream	AKR1C1^ *^	tagSNP
rs12414884	209699_x_at		**0.0033**	0.0059		upstream	AKR1C2^ *^	tagSNP
rs10904392	209699_x_at		**0.0216**	0.0221		upstream	AKR1C2^ *^	tagSNP
rs17134158	211653_x_at		**0.0176**		0.0391	upstream	AKR1C2^ *^	tagSNP
rs12414884	209160_at		**0.0383**	0.0368		upstream	AKR1C3^ *^	tagSNP
rs12105811	209939_x_at	0.0054	**0.0062**	0.0148		intron	CFLAR	ARE SNP
rs12105811	209508_x_at		**0.035**		0.0421	intron	CFLAR	ARE SNP
rs3810427	206153_at	0.0499	**0.0412**	0.0483		upstream	CYP4F11	ARE SNP
rs6588537	200862_at	0.0164	**0.0288**		0.0479	upstream	DHCR24	ARE SNP
rs17658295	201041_s_at	0.0138	**0.0046**	0.0111		upstream	DUSP1	ARE SNP
rs3753660	202017_at	0.0206	**0.0309**		0.0017	upstream	EPHX1^ *^	ARE SNP
rs17883018	203665_at	0.0191	**0.0249**	0.0352		intron	HMOX1^ *^	ARE SNP
rs3756273	211548_s_at	0.0056	**0.017**	0.0081		upstream	HPGD	ARE SNP
rs35258303	219212_at	0.0108	**0.0176**		0.0202	upstream	HSPA14	ARE SNP
rs2071204	209100_at	0.0405	**0.0109**		0.012	upstream	IFRD2	ARE SNP
rs2256974	215633_x_at	0.048	**0.0232**	0.0059		intron	LST1	ARE SNP
rs7037941	204917_s_at		**0.0047**		0.0045	intron	MLLT3	ARE SNP
rs10098474	219281_at		**0.0294**	0.0244		upstream	MSRA	ARE SNP
rs3762111	201602_s_at	0.0052	**0.0294**	0.0116		upstream	PPP1R12A	ARE SNP
***Significant in smokers (SC+SNC)***								
rs35408448	202888_s_at	0.0436	**0.0463**			upstream	ANPEP	ARE SNP
rs12105811	211862_x_at		**0.0406**			Intron	CFLAR	ARE SNP
rs17047438	208896_at		**0.0236**			upstream	DDX18	ARE SNP
rs4754450	203647_s_at		**0.033**			upstream	FDX1	ARE SNP
rs10078827	222034_at		**0.0475**			upstream	GNB2L1	ARE SNP
rs840466	215446_s_at	0.028	**0.0139**			upstream	LOX	ARE SNP
rs2658718	209861_s_at	0.0239	**0.0273**			upstream	METAP2	ARE SNP
rs631744	202884_s_at	0.0451	**0.0495**			upstream	PPP2R1B	ARE SNP
rs337253	218989_x_at		**0.0427**			Intron	SLC30A5	ARE SNP
rs10904392	216594_x_at		**0.0215**			downstream	AKR1C1^ *^	tagSNP
rs17134158	204151_x_at		**0.05**			downstream	AKR1C1^ *^	tagSNP
rs12414884	211653_x_at		**0.0143**			upstream	AKR1C2^ *^	tagSNP
rs17134158	209699_x_at		**0.0262**			upstream	AKR1C2^ *^	tagSNP
rs1805419	212788_x_at		**0.0395**			upstream	FTL^ *^	tagSNP
rs737777	203665_at	0.0178	**0.0131**			downstream	HMOX1^ *^	tagSNP
MAFG upstream SNP	204970_s_at	0.0012	**0.0037**			upstream	MAFG	new SNP
***Significant in all subjects(SC+SNC+NS)***								
rs504348	202805_s_at	0.0469				upstream	ABCC1 *	ARE SNP
rs2397146	202923_s_at	0.0387				downstream	GCLC *	tagSNP
rs670548	202923_s_at	0.0029		0.0476		upstream	GCLC *	tagSNP
MAFG 3′UTR SNP	204970_s_at	0.0317				3′UTR	MAFG	new SNP

The linear regression model was used to evaluate the association between log2-transformed expression values of a probe-set and genotypes of a SNP. An association was considered significant if the p-value from the analysis of the observed data was lower than the threshold of the 0.05 tail of the distribution of the minimal p-values from 10,000 permutations. Our focus was the association in all smokers (e.g. p-value in SC (n = 20) and SNC (n = 24), shown in bold fonts). We also tested the association in all samples (including SC (n = 20), SNC (n = 24), and NS (n = 8)), and in sub-groups (ie.: SC, SNC), and the results are displayed below. Only p-values less that 0.05 are displayed. Known ARE genes marked with *.

A few SNPs were associated with expression of known ARE genes among all 52 subjects but not within all smokers. For example, two SNPs (rs670548 and rs2397146) in the glutamate-cysteine ligase catalytic subunit (GCLC) gene that were not in LD (r^2^ = 0.28), were independently associated with expression (Figure 5C). Interestingly, the minor allele of rs670548 (in intron) was associated with low expression while the minor allele of rs2397146 in 5′ upstream region was associated with high expression (Figure 5D), suggesting the possibility of two distinct allelic phenotypes. Variation in GCLC has previously been associated with low level of lung function in two independent populations [23].

### SNPs that may contribute to cancer status via gene expression

**Figure 6:**
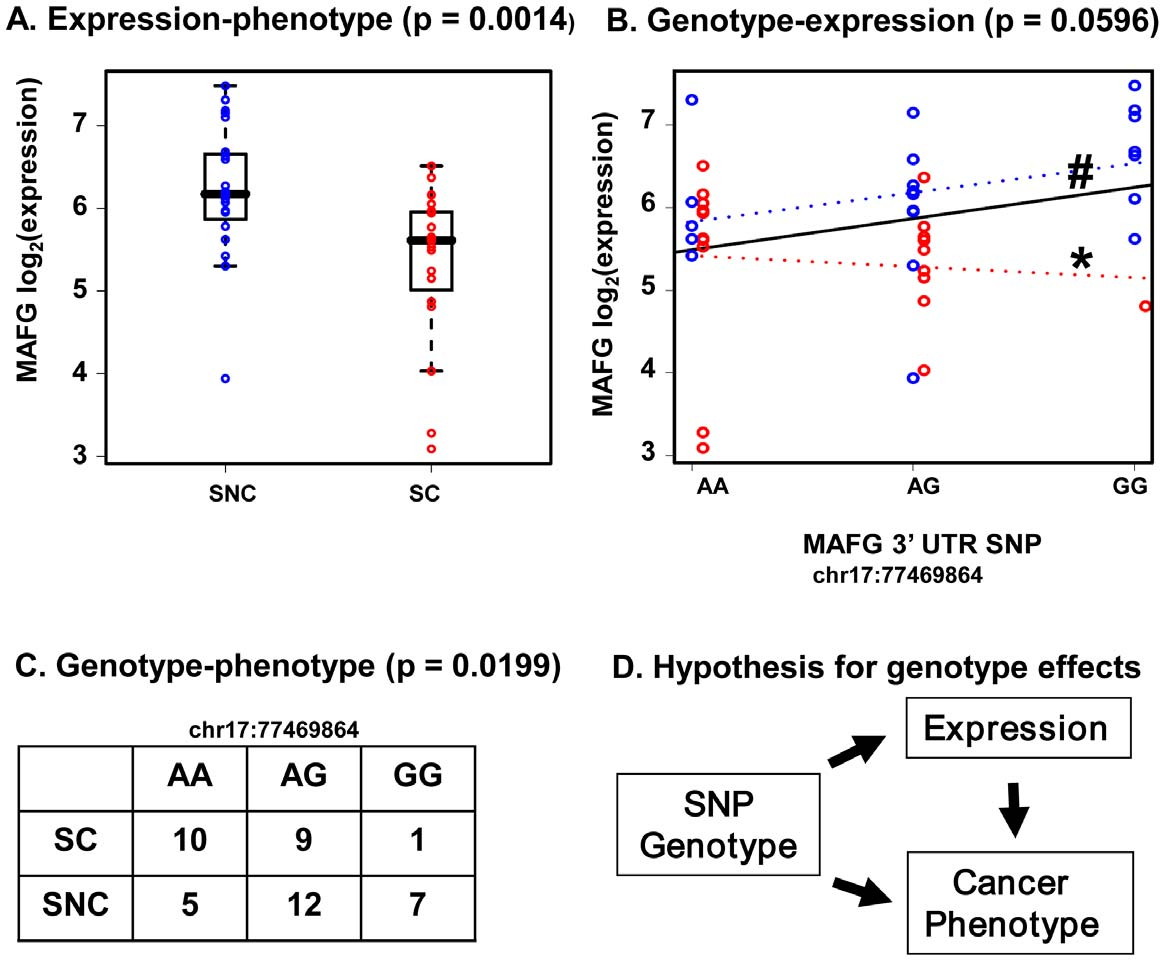
An example of how our three-part approach for association analysis of expression, genotype, and phenotype data may reveal biologically plausible SNPs. The MAFG 3′UTR SNP at chr17:77469864 may potentially contribute to phenotype (lung cancer status) via gene expression, based on: (A) Expression of MAFG was higher in smokers without cancer than that in smokers with cancer; (B) Genotype GG displays a trend toward higher expression levels of MAFG (#, black line); slopes of genotype by expression plots differ between groups (* red dotted line vs blue dotted line); (C) Genotype GG, associated with higher expression, was more common in smokers without cancer; (D) Hypothesis. Individuals with genotype GG display higher MAFG expression in bronchial epithelial cells; MAFG expression higher in smokers without cancer suggesting it is protective against lung cancer; GG genotype is less frequent among cancer group, consistent with a protective effect.

To identify SNPs that might affect lung cancer via gene expression we first used logistic regression to test the relationship between cancer or non-cancer status and log2-transformed gene expression levels. This identified 34 probe-sets (31 genes) in the antioxidant response pathway that were associated with cancer status of smokers at a corrected p-value < = 0.05. Notably, the MAFG probe-set 204970_s_at was associated with “without cancer” status (p = 0.0014) (Figure 6A). As outlined in Figure 6, we reasoned that a SNP that affected gene expression might differ in frequency among groups. While the statistical power for such a comparison is low, we did observe such an effect for the MAFG 3′UTR SNP mentioned previously (chr17:77469864; p = 0.058) (Figure 6B–C). As shown in Figure 6, the GG genotype was associated with the “without cancer” status of smokers (p = 0.0199), and also marginally with the higher expression level of MAFG. If these frequency differences could be substantiated in larger groups of patients, they could indicate protective or risk alleles and might be useful for predicting risk.

## Discussion

There is an important heritable component to lung cancer [24] and understanding how genetic variation alters smoking-induced gene expression could provide genetic biomarkers for diagnosis and reveal genetic susceptibility alleles. Numerous studies of human airway [5], [7], [25], [26]mouse lung [27] or *in vitro* cell culture [28] have reported on gene expression signatures related to smoking. Spira *et al* identified gene expression profiles in cytologically normal large-airway epithelial cells that can serve as a diagnostic biomarker for lung cancer [7]. The present work identifies molecular and genetic features of the NRF2-regulated pathway that are central to this airway gene expression response. Using pathway analysis tools, we identified differences in NRF2-mediated transcription profiles from bronchial airway epithelial cells obtained from nonsmokers, cigarette smokers with suspicion of lung cancer, and those with a subsequent diagnosis of lung cancer. We also revealed a potential role for MAFG (a NRF2-binding partner) in modulating smoking-induced gene expression.

NRF2 is activated by oxidative stress and translocates to the nucleus where it heterodimerizes with small MAF proteins to form a transactivation complex that binds to specific DNA regions termed antioxidant response elements (ARE) [29] and up-regulates antioxidant and phase II detoxification enzymes. We examined the expression of NRF2 and its interacting partners (e.g. MAFG, NRF1, NRF3, and BACH1) and made a novel observation that MAFG expression was strongly correlated with expression of downstream NRF2 target genes. At present, there are 42 NRF2 target genes discovered in various human tissues, and we found 22 of them correlated with MAFG gene expression level in human airway epithelial cells. In addition, NRF1, a negative and competitive regulatory factor, was anti-correlated with downstream antioxidant gene expression. The possibility that MAFG expression might limit downstream antioxidant gene expression was explored further. Silencing MAFG with siRNA in A549 cells attenuated the expression of known ARE genes (Figure 3B) and this was consistent with published experiments in MafG knockout mice [29]. A similar pattern for MAFG expression relative to other antioxidant genes was found when we carried out a retrospective analysis of expression data from a related, previously-published, larger-scale study [7]. We pursued a possible genetic cause for reduced MAFG expression among the SC group by re-sequencing MAFG in the subjects in this study and uncovered more than 30 novel SNPs in the 16.5-kb MAFG region. A SNP at chr17:77482956 in the MAFG promoter region was associated with lower MAFG mRNA levels, while another in the 3′ UTR displayed a marginal association with expression and lung cancer status (Figure 6). Thus some of the variability in gene expression among groups may be due to genetic variation, but it is also likely that other regulatory mechanisms, as well as the timing and duration of cigarette smoking in these patients have affected MAFG levels.

To explore how genetic variation in other NRF2 pathway genes might contribute to smoking-induced lung cancer disease susceptibility, we tested the association of many genotypes with both expression and/or group phenotype. We observed possible *cis*-acting effects for putative regulatory SNPs on genes affected by cigarette smoking and these could impact susceptibility of the airway to smoking-related diseases through various mechanisms, including metabolism of carcinogens. For example, members of the aldo-keto reductase (AKR) superfamily, AKR1C1, AKR1C2, and AKR1C3, catalyze the conversion of aldehydes and ketones to their corresponding alcohols by utilizing NADH and/or NADPH as cofactors. Polymorphisms of AKR1C3 have been implicated in susceptibility to various types of cancer, including lung cancer [30], [31], [32]. Microsomal epoxide hydrolase 1 (EPHX1) plays an important role in both the activation and detoxification of tobacco-derived carcinogens. Polymorphisms at exons 3 and 4 of the EPHX1 gene have been associated with variation in EPHX1 activity and a low-activity genotype of EPHX1 gene was associated with decreased risk of lung cancer among whites [33]. While the associations we found in this hypothesis-generating study were modest and need to be confirmed by follow-up in larger studies, we suggest that this approach may prove useful for identifying functional SNPs that contributing to a phenotype via an impact on gene expression.

Disease-association studies, both candidate gene-based and genome-wide association studies (GWAS), have identified genetic variants that associate with both monogenic and complex diseases like lung cancer. Recently several genomic regions that may affect nicotine metabolism or dependency in lung cancer patients were identified by GWAS with very high statistical significance [34], [35]. Presumably individuals with these genetic traits may use more tobacco and receive higher doses of the carcinogenic compounds in cigarette smoke. However, while the variants identified in the lung cancer GWAS studies, and in many other GWAS, point to potentially important loci, the functional relationship between the SNP and a molecular genetic mechanism to explain the biological phenotype is not apparent. Thus, understanding the molecular genetic basis of human phenotypic variation still remains a major challenge for genetics.

The approach we have used integrates information about gene expression in target tissue, variation in transcription factor binding sites and genotype frequency among cancer status groups in order to identify biologically-plausible functional polymorphisms. It could be generally useful for identifying SNPs that contribute to disease risk through their impact on gene expression. While the present study is limited by statistical power, applying this method to larger studies of exposed-tissue samples from clinically characterized patients may reveal useful expression-based and/or genetic biomarkers and provide a basis for prevention efforts.

## Materials and Methods

### Study population

Previously we recruited a group of healthy never smokers (<100 cigarettes in their lifetime; no second hand smoke) to undergo bronchoscopy at Boston University Medical Center [5], as well as a cohort of current and former smokers who were undergoing bronchoscopy as a diagnostic study for clinical suspicion of lung cancer at four institutions: Boston University Medical Center, Boston Veterans Administration, Lahey Clinic and St. James's Hospital [7].

### Ethics Statement

The study was approved by the Institutional Review Boards of all medical centers, and all participants provided written informed consent.

### Airway epithelial cell collection

Clinical subjects in the latter cohort were classified as either having lung cancer if their bronchoscopy or subsequent lung biopsy yielded lung tumor cells or not having lung cancer if the bronchoscopy or subsequent lung biopsy yielded a non-lung-cancer pathology (or if their radiographic abnormality resolved on follow-up chest imaging). Bronchial airway epithelial cells were obtained from the uninvolved right mainstem bronchus with an endoscopic cytobrush (Cellebrity Endoscopic Cytobrush, Boston Scientific). RNA was extracted and its integrity and epithelial cell content was confirmed as described previously [7].

Individuals with final diagnoses as of July 2008 and with blood genomic DNA for genotyping were included in this study, consisting of 20 smokers with lung cancer (SC), 24 smokers without lung cancer (SNC), and 8 never-smokers (NS) (Table 1). We observed significant differences in age between SC and SNC groups (p<0.05 by t-tests); no significant difference in gender, race, or cumulative tobacco exposure was found between SC and SNC groups. Because age was significantly different between these groups of patients, we tested the association between log2-expression values and age using linear regression. At the False Discovery Rate (FDR) 0.1 level, we found that 12 probe-sets (11 genes) correlated level with age (Supporting Information S1), and these probes were excluded from further analysis.

### Microarray data acquisition and preprocessing

Approximately eight micrograms of total RNA was processed, labeled and hybridized to Affymetrix HG-U133A GeneChips containing 22,283 probe-sets as described previously [7]. The MAS 5.0 algorithm was used for background adjustment, normalization, and probe-level summarization of microarray data [36].

### GEO accession number

All microarray data have been submitted to the Gene Expression Omnibus (GEO) under accession number GSE 19027.

### Gene set enrichment analysis (GSEA)

GSEA is a computational method [11], [37] that determines whether any *a priori* defined set of genes were enriched at the top or bottom of a list of genes ordered on the basis of expression difference between two biological states (e.g. phenotypes). It takes normalized array intensity values, rank genes according to score (e.g., signal-to-noise) between phenotype groups. It then walks through the ranked list of genes to calculate Enrichment Score (ES) for a gene set. In this study, we focused on the canonical pathways, i.e.: the c2.cp library of “molecular signature database” of GSEA software (v2.0.4), which included 639 curated gene sets (canonical pathways) from online pathway databases, publications in PubMed, and knowledge of domain experts. In addition, we tested a set of “antioxidant response element genes”, which included 37 human genes that have *bona fide* NRF2 binding sites (or antioxidant response elements), curated by mining PubMed database [9].

### Ingenuity Pathway Analysis (IPA)

IPA is software for identifying pathways most relevant to experimental datasets (http://www.ingenuity.com). Genes from the data set that met the FDR cutoff (q≤0.1) and were associated with biological functions in the Ingenuity Pathways Knowledge Base were considered for analysis. Fisher's exact test was used to determine the probability that each biological function assigned to that data set was due to chance alone.

### NRF2 gene silencing and gene expression analysis

We silenced MAFG in A549 cells by transiently transfecting small interfering RNA (siRNA) targeted to MAFG mRNA. Cells at 90% confluence were transfected with 0.4 µM siRNA (MAFG, ID# s8419 or control, ID# 4390843, Ambion) in the presence of 4.7 µl Lipofectamine 2000 transfection reagent (Invitrogen) for each well of a 6-well plate. We incubated transfected cells in antibiotic-free Ham's F12K without serum for 24 h before cell lysate collection. RNA was collected using RNeasy kit (Qiagen), including DNase treatment, and reverse transcribed using Superscript II cDNA synthesis kit (Invitrogen), following manufacturer's instructions. NQO1, GCLC, TXNRD1, MAFG, and ACTB (beta actin) gene expression levels were measured using TaqMan assays (Applied Biosystems). For SLC7A11, we designed expression primers using Primer3 (Rozen and Skaletsky 2000) (fwd primer 5′- GGCTGCCTTCCCTGGGCAAC-3′, rev primer 5′- CAGCAGTAGCTGCAGGGCGTA-3′) and assayed using SYBR green (Applied Biosystems). For real time analysis we performed 40 PCR cycles using 15 seconds 95°C melting temperature and 1 minute 60°C annealing/extension temperature per cycle and measured fluorescence intensity with an ABI 7900HT and calculated initial fluorescence (R_o_ value) of each amplified sample using the method described by Peirson and colleagues (Peirson et al. 2003). We normalized all target values with ACTB mRNA values. Experiments were performed in triplicate; PCRs were carried out in triplicate and values are reported as mean ± standard error of the mean (SEM).

### SNP identification in MAFG locus by sequencing

A 16.5-kb genomic region of the MAFG locus, starting 5000-nt upstream of the transcription start site and ending 2000-nt from the transcription end site was sequenced. Briefly,30 primer sets were designed to amplify the MAFG region in DNA from 52 study participants. After amplification, bidirectional sequencing of amplicons was carried out with an ABI DNA sequencer (Applied Biosystems, Foster City, California). The SNPs were detected by PolyPhred software developed by University of Washington.

### Tag SNP selection

Tag SNPs were selected based on Hapmap genotyping data on 60 unrelated CEU individuals (U.S. Utah residents with ancestry from northern and western Europe), using the Tagger software [38] implemented in Haploview software [39]. An r^2^ threshold of 0.8, minor allele frequency threshold of 0.1 was used.

### ARE SNP selection

SNPs in putative NRF2 binding sites (or AREs) were selected using an approach developed by our group [9], [13], [40]. Briefly, we constructed a position weight matrix (PWM) for NRF2 (ARE motif) based on the collection of experimentally discovered AREs. We then predicted AREs in the dbSNP entries using the PWM method and mapped these SNPs to the upstream regions of genes. To select the most likely functional AREs, we examined evolutionary conservation by phylogenetic footprinting. Lastly, we prioritized ARE candidate SNPs based on microarray expression profiles from tissues in which NRF2 was either silenced or over-expressed.

### SNP genotyping

Utilizing Illumina's Assay Design Tool, we designed Illumina GoldenGate assays for candidate SNPs (putative functional SNPs and tag SNPs). DNA samples derived from whole blood were subjected to whole genome amplification prior to genotyping. Post-amplification DNA products were cleaned and genotyped according to the manufacturer's protocol on an Illumina BeadStation 500G GoldenGate genotyping platform. Genotypes were assigned using Illumina's Beadstudio v3.0 Genotyping software with GenTrain scores >0.25. A total of 450 SNPs, including 342 SNPs located in putative binding sites of transcription factor NRF2 and 108 tag SNPs for 16 known ARE genes were placed on Illumina Golden Gate arrays and used to genotype DNA from our study subjects.

### Association analyses and multiple testing correction

A linear regression model was used to evaluate the association between log2-transformed expression values of a probe-set and genotypes of a SNP as previously described [10], [22]. Fisher's exact test was used to evaluate the association between genotypes of a SNP and phenotypes (lung cancer status in smokers); and a logistic regression model was used to evaluate the association between log2-transformed expression values of a probe-set and lung cancer status in smokers. We performed 10,000 permutations of each expression value or phenotype relative to the genotypes. An association was considered significant if the p-value was lower than 0.05 as determined by the tail end of the distribution of the minimal p-values from 10,000 permutations. All association tests and permutations were performed using the PLINK software (v1.0.6), an open-source whole genome association analysis toolset [41], available at http://pngu.mgh.harvard.edu/purcell/plink/.

### Other statistical analysis

All other statistical analyses were accomplished using the R statistical software package (v2.8.0) and the SAS JMP statistical discovery software (v7, SAS Institute Inc., Cary, NC). Gene Ontology analysis was done through the GoMiner web interface (http://discover.nci.nih.gov/gominer/), developed by National Cancer Institute, Bethesda, Maryland [42].

## Supporting Information

Supporting Information S1 Supporting Tables and Figure.
(3.53 MB DOC)
