# Supplementary material for: Genetic Variation and Antioxidant Response Gene Expression in the Bronchial Airway Epithelium of Smokers at Risk for Lung Cancer
Source: PLoS One. 2010 Aug 3;5(8):e11934. doi: 10.1371/journal.pone.0011934 (PMC2914741; doi:10.1371/journal.pone.0011934)
Supplement: Supporting Information S1 — Supporting Tables and Figure. (3.53 MB DOC) [file pone.0011934.s001.doc]

Table S1. Subject information

| subject | patientID | age | sex | race | cancer type | cancer stage | smoking_status | current/former smoker | pack -years |
| --- | --- | --- | --- | --- | --- | --- | --- | --- | --- |
| SC1 | 216 | 65 | F | CAU | Non Small Cell | 4 | smoker with cancer | former | 47 |
| SC2 | 238 | 69 | F | CAU | Squamous | 3a | smoker with cancer | former | 100 |
| SC3 | 239 | 59 | M | CAU | Squamous | 1a | smoker with cancer | current | 43 |
| SC4 | 263 | 59 | M | CAU | Squamous | 3a | smoker with cancer | former | 76 |
| SC5 | 264 | 62 | M | CAU | Non Small Cell | 3a | smoker with cancer | former | 34 |
| SC6 | 266 | 62 | M | CAU | Squamous | 4 | smoker with cancer | former | 120 |
| SC7 | 267 | 66 | M | CAU | Squamous | 3b | smoker with cancer | current | 37 |
| SC8 | 268 | 77 | F | CAU | Non Small Cell | 3a | smoker with cancer | former | 80 |
| SC9 | 277 | 75 | M | CAU | Squamous | 1b | smoker with cancer | current | 55 |
| SC10 | 285 | 54 | M | CAU | Adenocarcinoma | 1b | smoker with cancer | current | 38 |
| SC11 | 349 | 83 | M | CAU | Non Small Cell | 4 | smoker with cancer | former | 80 |
| SC12 | 435 | 78 | M | CAU | Small Cell | Ext | smoker with cancer | former | 85 |
| SC13 | 446 | 70 | F | CAU | Adenocarcinoma | 1a | smoker with cancer | former | 9 |
| SC14 | 450 | 83 | M | CAU | Squamous |  | smoker with cancer | former | 57 |
| SC15 | 453 | 57 | M | AFA | Adenocarcinoma |  | smoker with cancer | current | 19.5 |
| SC16 | 457 | 75 | M | CAU | Non Small Cell | 3a | smoker with cancer | former | 23 |
| SC17 | 472 | 59 | M | CAU | Adenocarcinoma | ? | smoker with cancer | current | 75 |
| SC18 | 478 | 67 | M | CAU | Squamous | 3a | smoker with cancer | current | 75 |
| SC19 | 483 | 75 | M | CAU | Non Small Cell | 3a | smoker with cancer | former | 60 |
| SC20 | 485 | 57 | M | CAU | Adenocarcinoma | 4 | smoker with cancer | current | 51.2 |
| SNC1 | 26 | 71 | M | CAU | None | None | smoker without cancer | former | 122.5 |
| SNC2 | 77 | 48 | M | AFA | None | None | smoker without cancer | former | 3 |
| SNC3 | 78 | 27 | F | AFA | None | None | smoker without cancer | former | 0.5 |
| SNC4 | 102 | 39 | M | CAU | None | None | smoker without cancer | current | 25 |
| SNC5 | 103 | 30 | M | HIS | None | None | smoker without cancer | current | 4 |
| SNC6 | 114 | 32 | M | HIS | None | None | smoker without cancer | current | 2 |
| SNC7 | 121 | 23 | M | HIS | None | None | smoker without cancer | current | 7 |
| SNC8 | 187 | 23 | M | ASI | None | None | smoker without cancer | current | 7 |
| SNC9 | 269 | 71 | M | CAU | None | None | smoker without cancer | former | 70 |
| SNC10 | 312 | 69 | M | CAU | None | None | smoker without cancer | current | 114 |
| SNC11 | 313 | 58 | M | CAU | None | None | smoker without cancer | current | 22 |
| SNC12 | 317 | 71 | M | CAU | None | None | smoker without cancer | current | 232 |
| SNC13 | 318 | 71 | M | CAU | None | None | smoker without cancer | current | 80 |
| SNC14 | 319 | 69 | M | CAU | None | None | smoker without cancer | current | 25 |
| SNC15 | 323 | 79 | M | CAU | None | None | smoker without cancer | current | 64 |
| SNC16 | 359 | 75 | M | CAU | None | None | smoker without cancer | former | 66 |
| SNC17 | 373 | 34 | M | AFA | None | None | smoker without cancer | current | 17 |
| SNC18 | 406 | 45 | F | AFA | None | None | smoker without cancer | current | 14 |
| SNC19 | 430 | 63 | M | CAU | None | None | smoker without cancer | former | 84 |
| SNC20 | 441 | 65 | M | CAU | None | None | smoker without cancer | current | 99 |
| SNC21 | 456 | 44 | M | AFA | None | None | smoker without cancer | current | 45 |
| SNC22 | 459 | 40 | M | HIS | None | None | smoker without cancer | current | 27.5 |
| SNC23 | 462 | 50 | M | CAU | None | None | smoker without cancer | current | 57 |
| SNC24 | 508 | 65 | M | OTH | None | None | smoker without cancer | former | 63 |
| NS1 | 90 | 28 | M | CAU |  |  | Never smoker |  | 0 |
| NS2 | 91 | 31 | M | CAU |  |  | Never smoker |  | 0 |
| NS3 | 95 | 28 | M | AFA |  |  | Never smoker |  | 0 |
| NS4 | 97 | 27 | F | CAU |  |  | Never smoker |  | 0 |
| NS5 | 98 | 68 | M | CAU |  |  | Never smoker |  | 0 |
| NS6 | 116 | 25 | M | CAU |  |  | Never smoker |  | 0 |
| NS7 | 460 | 26 | F | HIS |  |  | Never smoker |  | 0 |
| NS8 | 464 | 23 | F | OTH |  |  | Never smoker |  | 0 |

Table S2. Gene expression probes associated with age in the study population. Because age differed significantly among these groups of patients, we tested the association between log2-expression values and age using linear regression. At a FDR 0.1 level, we found that expression of 12 probe-sets (11 genes) correlated with age, and these probes were excluded from further analysis.

| FDR q | Probe | average expression SC | average expression  SNC | average expression  NS | Symbol |
| --- | --- | --- | --- | --- | --- |
| 0.0614 | 202912_at | 6.25 | 5.96 | 4.12 | ADM |
| 0.0029 | 203963_at | 8.02 | 7.56 | 5.85 | CA12 |
| 0.0033 | 215867_x_at | 8.12 | 7.95 | 6.54 | CA12 |
| 0.003 | 220389_at | 6.97 | 7.23 | 8.48 | CCDC81 |
| 0.0104 | 41660_at | 7.26 | 7.52 | 8.15 | CELSR1 |
| 0.0578 | 221676_s_at | 6.76 | 6.94 | 5.83 | CORO1C |
| 0.007 | 210835_s_at | 10.44 | 10.06 | 9.74 | CTBP2 |
| 0.0088 | 205403_at | 5.96 | 5.31 | 3.48 | IL1R2 |
| 0.0805 | 202018_s_at | 5.03 | 5.67 | 7.07 | LTF |
| 0.0012 | 214722_at | 7.57 | 8.04 | 9.33 | NOTCH2NL |
| 0.0434 | 219382_at | 6.90 | 6.73 | 6.42 | SERTAD3 |
| 0.0596 | 221562_s_at | 5.68 | 5.56 | 6.69 | SIRT3 |

Table S3. Differentially expressed probes identified by comparing smokers without cancer (SC) or smokers without cancer (SNC) with never-smokers (NS). To examine the effect of cigarette smoking on the bronchial airway transcriptome, we used profiles from never-smokers as baseline and compared the average log2-expression values of SC or SNC with that of NS by t-test, followed by multiple testing correction (Benjamini-Hochberg false discovery rate, FDR). Compared with never-smokers, we found differential expression (FDR 0.1) for 846 probe-sets (774 genes) in smokers without cancer, and 919 probe-sets (834 genes) in smokers with cancer.

| probe | avg_expr SC | avg_expr SNC | avg_expr NS | fdr SC_NS | ttest SC_NS | fdr SNC_NS | ttest SNC_NS | symbol |
| --- | --- | --- | --- | --- | --- | --- | --- | --- |
| 200804_at | 10.54 | 10.98 | 11.15 | 0.473 | 0.191 | 0.091 | 0.007 | 'TEGT |
| 201199_s_at | 8.88 | 8.9 | 9.22 | 0.093 | 0.007 | 0.084 | 0.006 | 'PSMD1 |
| 208469_s_at | 4.65 | 4.65 | 5.74 | 0.073 | 0.004 | 0.059 | 0.002 | 'EGFL8 |
| 217978_s_at | 7.38 | 7.63 | 7.82 | 0.575 | 0.291 | 0.029 | 0 | 'UBE2Q1 |
| 218661_at | 6.39 | 6.4 | 6.93 | 0.079 | 0.005 | 0.139 | 0.016 | 'FLJ14154 |
| 218153_at | 7.36 | 6.89 | 7.65 | 0.022 | 0 | 0.33 | 0.091 | 'CARS2 |
| 209240_at | 7.93 | 8.18 | 8.55 | 0.285 | 0.063 | 0.104 | 0.009 | 'OGT |
| 32259_at | 6.76 | 6.69 | 7.01 | 0.09 | 0.006 | 0.181 | 0.027 | 'EZH1 |
| 205749_at | 5.18 | 7.35 | 4.34 | 0.009 | 0 | 0.451 | 0.175 | 'CYP1A1 |
| 203852_s_at | 4.76 | 5.32 | 5.73 | 0.405 | 0.135 | 0.048 | 0.002 | 'SMN1|SMN2 |
| 211623_s_at | 9.09 | 8.91 | 9.6 | 0.075 | 0.004 | 0.172 | 0.024 | 'FBL |
| 44146_at | 7.58 | 7.33 | 7.7 | 0.042 | 0.001 | 0.606 | 0.331 | 'GMEB2 |
| 202748_at | 6.54 | 6.56 | 5.62 | 0.061 | 0.003 | 0.059 | 0.003 | 'GBP2 |
| 217865_at | 7.41 | 8.04 | 8.71 | 0.175 | 0.023 | 0.068 | 0.003 | 'RNF130 |
| 208620_at | 8.59 | 9.1 | 9.27 | 0.653 | 0.378 | 0.104 | 0.009 | 'PCBP1 |
| 213390_at | 6.68 | 6.63 | 7.11 | 0.085 | 0.005 | 0.213 | 0.038 | 'ZC3H4 |
| 200013_at | 10.49 | 10.59 | 11 | 0.17 | 0.022 | 0.103 | 0.009 | 'RPL24 |
| 207011_s_at | 5.97 | 5.74 | 6.61 | 0.065 | 0.003 | 0.131 | 0.014 | 'PTK7 |
| 221971_x_at | 7.48 | 7.69 | 8.29 | 0.028 | 0.001 | 0.009 | 0 | 'CTGLF2 |
| 218348_s_at | 4.78 | 5.59 | 5.88 | 0.453 | 0.175 | 0.045 | 0.001 | 'ZC3H7A |
| 203155_at | 5.55 | 5.75 | 6.32 | 0.173 | 0.023 | 0.078 | 0.005 | 'SETDB1 |
| 203923_s_at | 5.58 | 5.74 | 4.98 | 0.092 | 0.006 | 0.191 | 0.03 | 'CYBB |
| 208656_s_at | 10 | 10.2 | 10.59 | 0.106 | 0.008 | 0.05 | 0.002 | 'CCNI |
| 36711_at | 7.42 | 7.09 | 5.19 | 0.03 | 0.001 | 0.02 | 0 | 'MAFF |
| 212423_at | 5.79 | 5.66 | 6.51 | 0.037 | 0.001 | 0.075 | 0.004 | 'ZCCHC24 |
| 211654_x_at | 7.84 | 8.33 | 8.88 | 0.379 | 0.118 | 0.083 | 0.005 | 'HLA-DQB1 |
| 205379_at | 7.08 | 6.98 | 5.88 | 0.124 | 0.011 | 0.084 | 0.006 | 'CBR3 |
| 208371_s_at | 7.33 | 7.49 | 7.88 | 0.116 | 0.01 | 0.05 | 0.002 | 'RING1 |
| 209231_s_at | 6.26 | 5.97 | 5.71 | 0.443 | 0.165 | 0.087 | 0.006 | 'DCTN5 |
| 215749_s_at | 6.79 | 7.01 | 7.17 | 0.472 | 0.19 | 0.053 | 0.002 | 'GORASP1 |
| 202518_at | 6.49 | 6.55 | 7.05 | 0.077 | 0.004 | 0.131 | 0.014 | 'BCL7B |
| 203911_at | 6.98 | 7.01 | 6.12 | 0.028 | 0.001 | 0.042 | 0.001 | 'RAP1GAP |
| 38290_at | 5.37 | 5.25 | 6.1 | 0.059 | 0.003 | 0.091 | 0.007 | 'RGS14 |
| 213412_at | 6.76 | 7.12 | 8.18 | 0.013 | 0 | 0.043 | 0.001 | 'TJP3 |
| 209369_at | 6.32 | 6.61 | 5.63 | 0.042 | 0.001 | 0.12 | 0.012 | 'ANXA3 |
| 212449_s_at | 7.55 | 7.43 | 6.68 | 0.126 | 0.012 | 0.073 | 0.004 | 'LYPLA1 |
| 214092_x_at | 5.59 | 5.55 | 6.5 | 0.016 | 0 | 0.017 | 0 | 'SFRS14 |
| 212861_at | 7.5 | 7.25 | 7.12 | 0.568 | 0.283 | 0.035 | 0.001 | 'MFSD5 |
| 219488_at | 5.99 | 5.98 | 6.48 | 0.035 | 0.001 | 0.115 | 0.011 | 'A4GALT |
| 202440_s_at | 8.47 | 8.26 | 8.68 | 0.058 | 0.003 | 0.4 | 0.135 | 'ST5 |
| 205680_at | 6.81 | 6.36 | 7.99 | 0.004 | 0 | 0.032 | 0.001 | 'MMP10 |
| 201104_x_at | 7.83 | 8.34 | 8.54 | 0.541 | 0.255 | 0.055 | 0.002 | 'NBPF11 |
| 212886_at | 6.91 | 6.67 | 7.59 | 0.029 | 0.001 | 0.128 | 0.013 | 'CCDC69 |
| 206461_x_at | 9.26 | 9.27 | 10.33 | 0.061 | 0.003 | 0.062 | 0.003 | 'MT1H|MT1P2 |
| 221689_s_at | 8.24 | 8.4 | 7.86 | 0.09 | 0.006 | 0.276 | 0.062 | 'PIGP |
| 204754_at | 5.74 | 5.34 | 6.18 | 0.04 | 0.001 | 0.251 | 0.052 | 'HLF |
| 211883_x_at | 6.14 | 6.62 | 5.82 | 0.099 | 0.007 | 0.512 | 0.23 | 'CEACAM1 |
| 218067_s_at | 7.23 | 7.59 | 8.11 | 0.036 | 0.001 | 0.029 | 0 | 'ARGLU1 |
| 205847_at | 6.83 | 7.13 | 7.33 | 0.524 | 0.236 | 0.091 | 0.007 | 'PRSS22 |
| 203660_s_at | 6.74 | 6.55 | 7.4 | 0.017 | 0 | 0.047 | 0.002 | 'PCNT |
| 203554_x_at | 8.1 | 7.93 | 7.28 | 0.153 | 0.018 | 0.082 | 0.005 | 'PTTG1 |
| 201861_s_at | 7.53 | 7.73 | 8.37 | 0.14 | 0.015 | 0.059 | 0.002 | 'LRRFIP1 |
| 205323_s_at | 7.51 | 7.75 | 8.05 | 0.139 | 0.014 | 0.038 | 0.001 | 'MTF1 |
| 215438_x_at | 7.25 | 7.37 | 6.77 | 0.063 | 0.003 | 0.191 | 0.03 | 'GSPT1 |
| 201952_at | 11.66 | 11.45 | 11.78 | 0.088 | 0.006 | 0.602 | 0.324 | 'ALCAM |
| 209835_x_at | 7.37 | 7.61 | 6.99 | 0.059 | 0.003 | 0.263 | 0.057 | 'CD44 |
| 221989_at | 5.52 | 6.11 | 6.8 | 0.097 | 0.007 | 0.017 | 0 | 'RPL10 |
| 220046_s_at | 6.25 | 7.09 | 7.25 | 0.618 | 0.336 | 0.055 | 0.002 | 'CCNL1 |
| 217678_at | 5.51 | 5.83 | 4.86 | 0.048 | 0.002 | 0.2 | 0.033 | 'SLC7A11 |
| 204053_x_at | 6.7 | 6.77 | 6.42 | 0.072 | 0.004 | 0.15 | 0.018 | 'PTEN |
| 212186_at | 7.15 | 7.36 | 7.93 | 0.109 | 0.009 | 0.096 | 0.007 | 'ACACA |
| 214211_at | 9.77 | 9.98 | 9.17 | 0.002 | 0 | 0.032 | 0.001 | 'FTH1 |
| 200875_s_at | 7.41 | 7.47 | 7.85 | 0.145 | 0.016 | 0.086 | 0.006 | 'NOL5A |
| 209225_x_at | 8.89 | 8.47 | 7.81 | 0.103 | 0.008 | 0.031 | 0 | 'TNPO1 |
| 213645_at | 6.87 | 7.19 | 7.65 | 0.272 | 0.057 | 0.094 | 0.007 | 'ENOSF1 |
| 208791_at | 9.7 | 10 | 10.82 | 0.124 | 0.011 | 0.07 | 0.004 | 'CLU |
| 211941_s_at | 9.29 | 9.65 | 10.26 | 0.155 | 0.018 | 0.058 | 0.002 | 'PEBP1 |
| 210020_x_at | 6.97 | 6.18 | 5.8 | 0.398 | 0.129 | 0.024 | 0 | 'CALML3 |
| 219809_at | 7.12 | 7 | 6.77 | 0.382 | 0.12 | 0.074 | 0.004 | 'WDR55 |
| 211734_s_at | 3.97 | 4.4 | 5.39 | 0.137 | 0.014 | 0.052 | 0.002 | 'FCER1A |
| 211004_s_at | 8.6 | 8.77 | 9.55 | 0.063 | 0.003 | 0.064 | 0.003 | 'ALDH3B1 |
| 213568_at | 4.47 | 4.11 | 5.21 | 0.088 | 0.006 | 0.292 | 0.07 | 'OSR2 |
| 202622_s_at | 6.37 | 6.79 | 7.21 | 0.199 | 0.03 | 0.067 | 0.003 | 'ATXN2 |
| 205997_at | 6.16 | 7.03 | 7.6 | 0.229 | 0.04 | 0.043 | 0.001 | 'ADAM28 |
| 202722_s_at | 7.84 | 8.1 | 7.31 | 0.029 | 0.001 | 0.115 | 0.011 | 'GFPT1 |
| 200058_s_at | 8.34 | 8.32 | 8.84 | 0.058 | 0.003 | 0.102 | 0.009 | 'ASCC3L1 |
| 205928_at | 5.62 | 5.56 | 6.39 | 0.104 | 0.008 | 0.2 | 0.033 | 'ZNF443 |
| 217890_s_at | 5.74 | 6.5 | 6.86 | 0.463 | 0.181 | 0.089 | 0.006 | 'PARVA |
| 202888_s_at | 4.82 | 4.6 | 5.84 | 0.066 | 0.003 | 0.094 | 0.007 | 'ANPEP |
| 204059_s_at | 6.4 | 6.81 | 5.22 | 0.006 | 0 | 0.038 | 0.001 | 'ME1 |
| 211986_at | 8.92 | 9.25 | 9.92 | 0.04 | 0.001 | 0.047 | 0.001 | 'AHNAK |
| 208653_s_at | 5.86 | 6.34 | 5.2 | 0.09 | 0.006 | 0.309 | 0.079 | 'CD164 |
| 209019_s_at | 7.15 | 7.26 | 7.94 | 0.045 | 0.002 | 0.08 | 0.005 | 'PINK1 |
| 201884_at | 10.68 | 11.31 | 8.38 | 0.002 | 0 | 0.014 | 0 | 'CEACAM5 |
| 210037_s_at | 5.4 | 5.63 | 6.75 | 0.143 | 0.015 | 0.083 | 0.005 | 'NOS2A |
| 202978_s_at | 5.16 | 5.71 | 5.81 | 0.783 | 0.57 | 0.082 | 0.005 | 'CREBZF |
| 218204_s_at | 6.29 | 6.62 | 7.03 | 0.037 | 0.001 | 0.096 | 0.008 | 'FYCO1 |
| 202356_s_at | 7.3 | 6.92 | 6.64 | 0.472 | 0.19 | 0.078 | 0.005 | 'GTF2F1 |
| 202775_s_at | 6.71 | 6.65 | 7.1 | 0.022 | 0 | 0.055 | 0.002 | 'SFRS8 |
| 205009_at | 7.93 | 7.73 | 6.52 | 0.026 | 0.001 | 0.029 | 0 | 'TFF1 |
| 205970_at | 6.47 | 6.22 | 7.15 | 0.044 | 0.002 | 0.115 | 0.011 | 'MT3 |
| 207266_x_at | 6.38 | 7.05 | 7.61 | 0.26 | 0.052 | 0.075 | 0.004 | 'RBMS1 |
| 208918_s_at | 7.22 | 7.1 | 6.42 | 0.007 | 0 | 0.009 | 0 | 'NADK |
| 206483_at | 7.51 | 7.63 | 8.01 | 0.037 | 0.001 | 0.085 | 0.006 | 'LRRC6 |
| 206798_x_at | 8.25 | 8.37 | 9.02 | 0.053 | 0.002 | 0.02 | 0 | 'DLEC1 |
| 209290_s_at | 7.63 | 7.48 | 8.18 | 0.038 | 0.001 | 0.124 | 0.013 | 'NFIB |
| 218103_at | 7.99 | 7.62 | 8.06 | 0.079 | 0.005 | 0.805 | 0.614 | 'FTSJ3 |
| 220192_x_at | 8.12 | 8.94 | 7.66 | 0.006 | 0 | 0.405 | 0.138 | 'SPDEF |
| 206492_at | 5.42 | 5.26 | 6.03 | 0.034 | 0.001 | 0.136 | 0.015 | 'FHIT |
| 202581_at | 8.04 | 7.74 | 6.99 | 0.343 | 0.094 | 0.101 | 0.008 | 'HSPA1B |
| 32137_at | 7.84 | 7.63 | 8.55 | 0.002 | 0 | 0.009 | 0 | 'JAG2 |
| 219275_at | 4.82 | 5.05 | 5.66 | 0.145 | 0.016 | 0.065 | 0.003 | 'PDCD5 |
| 201161_s_at | 7 | 7.84 | 8.34 | 0.483 | 0.2 | 0.071 | 0.004 | 'CSDA |
| 211052_s_at | 6.46 | 6.29 | 7.14 | 0.044 | 0.002 | 0.171 | 0.024 | 'TBCD |
| 207718_x_at | 7.24 | 7.13 | 7.81 | 0.066 | 0.003 | 0.126 | 0.013 | 'CYP2A6 |
| 201477_s_at | 7.86 | 7.54 | 8.19 | 0.028 | 0.001 | 0.253 | 0.053 | 'RRM1 |
| 200793_s_at | 7.4 | 7.79 | 8.47 | 0.067 | 0.003 | 0.051 | 0.002 | 'ACO2 |
| 200083_at | 8.45 | 8.37 | 8.71 | 0.067 | 0.003 | 0.212 | 0.037 | 'USP22 |
| 213397_x_at | 8.31 | 8.14 | 7.21 | 0.047 | 0.002 | 0.031 | 0 | 'RNASE4 |
| 208127_s_at | 5.23 | 5.27 | 4.42 | 0.072 | 0.004 | 0.079 | 0.005 | 'SOCS5 |
| 209439_s_at | 5.89 | 5.99 | 6.6 | 0.045 | 0.002 | 0.035 | 0.001 | 'PHKA2 |
| 219028_at | 5.88 | 6.14 | 6.71 | 0.235 | 0.042 | 0.089 | 0.006 | 'HIPK2 |
| 201431_s_at | 7.22 | 7.6 | 6.55 | 0.069 | 0.004 | 0.227 | 0.043 | 'DPYSL3 |
| 200820_at | 7.71 | 7.84 | 8.51 | 0.126 | 0.012 | 0.103 | 0.009 | 'PSMD8 |
| 205926_at | 5.6 | 5.53 | 6.15 | 0.05 | 0.002 | 0.092 | 0.007 | 'IL27RA |
| 215714_s_at | 6.97 | 7.19 | 7.89 | 0.098 | 0.007 | 0.142 | 0.016 | 'SMARCA4 |
| 219526_at | 7.3 | 6.95 | 6.79 | 0.582 | 0.298 | 0.067 | 0.003 | 'C14orf169 |
| 221619_s_at | 10.36 | 10.3 | 10.81 | 0.052 | 0.002 | 0.107 | 0.009 | 'MTCH1 |
| 221741_s_at | 7.77 | 7.94 | 8.43 | 0.07 | 0.004 | 0.031 | 0 | 'YTHDF1 |
| 212775_at | 4.88 | 5.02 | 5.67 | 0.13 | 0.013 | 0.094 | 0.007 | 'OBSL1 |
| 211529_x_at | 10.54 | 10.9 | 11.33 | 0.311 | 0.076 | 0.037 | 0.001 | 'HLA-G |
| 217952_x_at | 6.16 | 6.35 | 6.92 | 0.205 | 0.032 | 0.075 | 0.004 | 'PHF3 |
| 213288_at | 7.29 | 7.55 | 7.16 | 0.059 | 0.003 | 0.606 | 0.33 | 'MBOAT2 |
| 211858_x_at | 9.66 | 10.27 | 11.2 | 0.135 | 0.014 | 0.037 | 0.001 | 'GNAS |
| 204645_at | 5 | 5.35 | 5.61 | 0.477 | 0.194 | 0.082 | 0.005 | 'CCNT2 |
| 208935_s_at | 5.34 | 5.46 | 6.01 | 0.105 | 0.008 | 0.071 | 0.004 | 'LGALS8 |
| 217903_at | 6.25 | 6.35 | 6.81 | 0.065 | 0.003 | 0.031 | 0 | 'STRN4 |
| 201004_at | 10.6 | 10.5 | 10.06 | 0.075 | 0.004 | 0.041 | 0.001 | 'SSR4 |
| 207959_s_at | 7.52 | 7.2 | 7.96 | 0.045 | 0.002 | 0.29 | 0.069 | 'DNAH9 |
| 208674_x_at | 7.97 | 7.67 | 7.22 | 0.299 | 0.07 | 0.098 | 0.008 | 'DDOST |
| 201760_s_at | 6.68 | 6.91 | 5.93 | 0.077 | 0.004 | 0.165 | 0.022 | 'WSB2 |
| 203506_s_at | 5.48 | 5.9 | 6.71 | 0.039 | 0.001 | 0.043 | 0.001 | 'MED12 |
| 218476_at | 5.99 | 5.62 | 6.45 | 0.024 | 0 | 0.166 | 0.022 | 'POMT1 |
| 208774_at | 7.34 | 7.54 | 8.28 | 0.047 | 0.002 | 0.101 | 0.008 | 'CSNK1D |
| 221875_x_at | 10.53 | 10.93 | 11.19 | 0.529 | 0.241 | 0.083 | 0.005 | 'HLA-F |
| 219753_at | 5.64 | 5.78 | 6.42 | 0.1 | 0.007 | 0.058 | 0.002 | 'STAG3 |
| 208633_s_at | 5.7 | 6.04 | 6.56 | 0.26 | 0.052 | 0.091 | 0.007 | 'MACF1 |
| 200072_s_at | 8.31 | 8.56 | 9.48 | 0.066 | 0.003 | 0.046 | 0.001 | 'HNRNPM |
| 200810_s_at | 9.45 | 9.4 | 10.16 | 0.033 | 0.001 | 0.057 | 0.002 | 'CIRBP |
| 202634_at | 6.17 | 6.16 | 5.48 | 0.103 | 0.008 | 0.081 | 0.005 | 'POLR2K |
| 204755_x_at | 5.92 | 5.39 | 6.41 | 0.024 | 0 | 0.159 | 0.021 | 'HLF |
| 213572_s_at | 8.24 | 8.22 | 7.36 | 0.09 | 0.006 | 0.081 | 0.005 | 'SERPINB1 |
| 209067_s_at | 7.1 | 7.45 | 7.94 | 0.282 | 0.061 | 0.027 | 0 | 'HNRPDL |
| 218387_s_at | 8.84 | 8.36 | 8.16 | 0.481 | 0.198 | 0.054 | 0.002 | 'PGLS |
| 217187_at | 8.5 | 8.77 | 7.53 | 0.002 | 0 | 0.019 | 0 | 'MUC5AC |
| 205652_s_at | 5.86 | 5.84 | 6.53 | 0.078 | 0.005 | 0.145 | 0.017 | 'TTLL1 |
| 204249_s_at | 7.7 | 7.89 | 8.38 | 0.09 | 0.006 | 0.07 | 0.004 | 'LMO2 |
| 214404_x_at | 7.86 | 8.74 | 7.11 | 0.001 | 0 | 0.147 | 0.017 | 'SPDEF |
| 220302_at | 6.47 | 6.52 | 6.88 | 0.081 | 0.005 | 0.082 | 0.005 | 'MAK |
| 218152_at | 5.39 | 5.54 | 6.06 | 0.09 | 0.006 | 0.08 | 0.005 | 'HMG20A |
| 212014_x_at | 6.9 | 7.21 | 6.56 | 0.04 | 0.001 | 0.346 | 0.102 | 'CD44 |
| 201229_s_at | 4.84 | 5.1 | 6.22 | 0.082 | 0.005 | 0.071 | 0.004 | 'ARIH2 |
| 209863_s_at | 8.16 | 7.94 | 8.76 | 0.013 | 0 | 0.061 | 0.003 | 'TP63 |
| 211538_s_at | 6.56 | 6.33 | 7.53 | 0.004 | 0 | 0.025 | 0 | 'HSPA2 |
| 206855_s_at | 6.29 | 6.62 | 6.96 | 0.246 | 0.047 | 0.089 | 0.006 | 'HYAL2 |
| 208984_x_at | 7.28 | 7.03 | 7.56 | 0.076 | 0.004 | 0.526 | 0.244 | 'RBM10 |
| 204000_at | 6.42 | 6.15 | 6.75 | 0.078 | 0.005 | 0.281 | 0.065 | 'GNB5 |
| 219314_s_at | 5.65 | 5.78 | 6.76 | 0.09 | 0.006 | 0.065 | 0.003 | 'ZNF219 |
| 212736_at | 6.1 | 6.28 | 7.08 | 0.066 | 0.003 | 0.037 | 0.001 | 'C16orf45 |
| 63825_at | 8.19 | 8.03 | 7.1 | 0.102 | 0.008 | 0.055 | 0.002 | 'ABHD2 |
| 209366_x_at | 9.45 | 9.86 | 10.58 | 0.083 | 0.005 | 0.02 | 0 | 'CYB5A |
| 214696_at | 6.66 | 6.98 | 6.5 | 0.044 | 0.002 | 0.714 | 0.471 | 'C17orf91 |
| 202384_s_at | 5.16 | 4.99 | 5.99 | 0.042 | 0.001 | 0.05 | 0.002 | 'TCOF1 |
| 205566_at | 7.56 | 7.67 | 6.9 | 0.004 | 0 | 0.016 | 0 | 'ABHD2 |
| 200644_at | 7.54 | 7.71 | 6.93 | 0.049 | 0.002 | 0.091 | 0.007 | 'MARCKSL1 |
| 214093_s_at | 5.12 | 5.03 | 5.75 | 0.072 | 0.004 | 0.09 | 0.006 | 'FUBP1 |
| 207559_s_at | 7.65 | 7.48 | 7.98 | 0.03 | 0.001 | 0.146 | 0.017 | 'ZMYM3 |
| 201275_at | 7.57 | 7.83 | 8.04 | 0.29 | 0.065 | 0.076 | 0.004 | 'FDPS |
| 212902_at | 5.46 | 5.63 | 5.25 | 0.08 | 0.005 | 0.437 | 0.164 | 'SEC24A |
| 213080_x_at | 11.22 | 11.14 | 11.83 | 0.03 | 0.001 | 0.051 | 0.002 | 'RPL5 |
| 204231_s_at | 6.11 | 6.06 | 6.79 | 0.022 | 0 | 0.039 | 0.001 | 'FAAH |
| 214459_x_at | 12.07 | 12.24 | 12.96 | 0.042 | 0.001 | 0.027 | 0 | 'HLA-C |
| 200775_s_at | 9.14 | 9.45 | 9.69 | 0.35 | 0.098 | 0.064 | 0.003 | 'HNRNPK |
| 201557_at | 6.87 | 6.77 | 7.27 | 0.07 | 0.004 | 0.168 | 0.023 | 'VAMP2 |
| 217740_x_at | 12.22 | 12.21 | 12.87 | 0.052 | 0.002 | 0.057 | 0.002 | 'RPL7A |
| 215136_s_at | 5.64 | 5.53 | 6.35 | 0.053 | 0.002 | 0.085 | 0.006 | 'EXOSC8 |
| 206959_s_at | 5.78 | 5.78 | 6.54 | 0.059 | 0.003 | 0.054 | 0.002 | 'UPF3A |
| 219108_x_at | 8.49 | 7.95 | 7.79 | 0.715 | 0.466 | 0.082 | 0.005 | 'DDX27 |
| 209550_at | 7.31 | 6.89 | 7.44 | 0.042 | 0.001 | 0.627 | 0.356 | 'NDN |
| 201468_s_at | 9.94 | 10.15 | 8.61 | 0 | 0 | 0.006 | 0 | 'NQO1 |
| 218914_at | 6.19 | 6.02 | 6.6 | 0.096 | 0.007 | 0.213 | 0.038 | 'C1orf66 |
| 210514_x_at | 8.87 | 9.48 | 9.74 | 0.664 | 0.392 | 0.06 | 0.003 | 'HLA-G |
| 209310_s_at | 6.24 | 6.41 | 6.68 | 0.442 | 0.165 | 0.09 | 0.007 | 'CASP4 |
| 203804_s_at | 8.25 | 7.78 | 7.36 | 0.371 | 0.113 | 0.078 | 0.005 | 'CROP |
| 217122_s_at | 7.87 | 7.97 | 8.49 | 0.034 | 0.001 | 0.038 | 0.001 | 'RP11-345P4.4 |
| 204753_s_at | 5.24 | 5.47 | 6.29 | 0.09 | 0.006 | 0.087 | 0.006 | 'HLF |
| 203340_s_at | 6.21 | 6.2 | 6.83 | 0.13 | 0.013 | 0.09 | 0.006 | 'SLC25A12 |
| 219040_at | 6.38 | 6.12 | 6.85 | 0.046 | 0.002 | 0.308 | 0.078 | 'CORO7 |
| 208845_at | 10.62 | 10.19 | 10.1 | 0.786 | 0.576 | 0.084 | 0.006 | 'VDAC3 |
| 205059_s_at | 6.4 | 6.38 | 6.87 | 0.156 | 0.018 | 0.096 | 0.007 | 'IDUA |
| 213726_x_at | 12.16 | 12.31 | 12.65 | 0.203 | 0.032 | 0.081 | 0.005 | 'TUBB2C |
| 210723_x_at | 4.98 | 5.2 | 5.84 | 0.217 | 0.036 | 0.078 | 0.005 | 'MGC4771 |
| 213534_s_at | 6.36 | 6.54 | 6.93 | 0.266 | 0.054 | 0.097 | 0.008 | 'PASK |
| 200786_at | 8.96 | 8.85 | 8.63 | 0.259 | 0.051 | 0.087 | 0.006 | 'PSMB7 |
| 221476_s_at | 10.6 | 10.61 | 11.35 | 0.032 | 0.001 | 0.048 | 0.002 | 'RPL15 |
| 33323_r_at | 11.03 | 10.34 | 9.97 | 0.268 | 0.055 | 0.038 | 0.001 | 'SFN |
| 218546_at | 6.06 | 5.2 | 6.07 | 0.09 | 0.006 | 0.99 | 0.974 | 'C1orf115 |
| 210469_at | 5.53 | 5.57 | 6.79 | 0.025 | 0 | 0.026 | 0 | 'DLG5 |
| 209355_s_at | 5.12 | 5.45 | 6.36 | 0.129 | 0.012 | 0.064 | 0.003 | 'PPAP2B |
| 201447_at | 5.76 | 6.3 | 6.29 | 0.972 | 0.938 | 0.087 | 0.006 | 'TIA1 |
| 210011_s_at | 6.73 | 7.24 | 7.9 | 0.121 | 0.011 | 0.048 | 0.002 | 'EWSR1 |
| 213254_at | 5.9 | 6.46 | 6.95 | 0.115 | 0.01 | 0.032 | 0.001 | 'TNRC6B |
| 47553_at | 5.41 | 5.68 | 6.47 | 0.107 | 0.009 | 0.047 | 0.002 | 'DFNB31 |
| 209610_s_at | 7.18 | 6.96 | 6.71 | 0.388 | 0.124 | 0.098 | 0.008 | 'SLC1A4 |
| 211963_s_at | 7.94 | 8.24 | 7.52 | 0.066 | 0.003 | 0.314 | 0.082 | 'ARPC5 |
| 200736_s_at | 10.14 | 9.87 | 9.32 | 0.215 | 0.036 | 0.078 | 0.005 | 'GPX1 |
| 221742_at | 7.2 | 7.39 | 8.2 | 0.019 | 0 | 0.024 | 0 | 'CUGBP1 |
| 209619_at | 10.3 | 11.16 | 11.54 | 0.492 | 0.207 | 0.023 | 0 | 'CD74 |
| 209403_at | 5.07 | 5.41 | 6.68 | 0.018 | 0 | 0.002 | 0 | 'TBC1D3B |
| 209215_at | 6.55 | 6.81 | 7.82 | 0.04 | 0.001 | 0.035 | 0.001 | 'MFSD10 |
| 33778_at | 8.58 | 8.39 | 8.21 | 0.388 | 0.124 | 0.081 | 0.005 | 'TBC1D22A |
| 218091_at | 6.76 | 6.73 | 6.31 | 0.045 | 0.002 | 0.04 | 0.001 | 'HRB |
| 201980_s_at | 6.35 | 6.56 | 5.82 | 0.032 | 0.001 | 0.104 | 0.009 | 'RSU1 |
| 209436_at | 6.15 | 5.98 | 5.61 | 0.322 | 0.082 | 0.085 | 0.006 | 'SPON1 |
| 207319_s_at | 4.39 | 4.65 | 5.22 | 0.218 | 0.037 | 0.075 | 0.004 | 'CDC2L5 |
| 52164_at | 6.7 | 6.45 | 6.19 | 0.412 | 0.141 | 0.057 | 0.002 | 'C11orf24 |
| 203630_s_at | 7.14 | 6.91 | 6.5 | 0.366 | 0.109 | 0.049 | 0.002 | 'COG5 |
| 208823_s_at | 7.41 | 7.5 | 7.92 | 0.173 | 0.023 | 0.083 | 0.006 | 'PCTK1 |
| 214870_x_at | 8.88 | 9.47 | 10.17 | 0.09 | 0.006 | 0.044 | 0.001 | 'NPIP |
| 33760_at | 6.48 | 6.21 | 6.87 | 0.06 | 0.003 | 0.243 | 0.049 | 'PEX14 |
| 213059_at | 9.02 | 9.19 | 8.59 | 0.043 | 0.001 | 0.146 | 0.017 | 'CREB3L1 |
| 200630_x_at | 9.99 | 10.03 | 10.49 | 0.044 | 0.002 | 0.033 | 0.001 | 'SET |
| 203028_s_at | 9.34 | 9.27 | 8.83 | 0.145 | 0.016 | 0.09 | 0.006 | 'CYBA |
| 202829_s_at | 6.79 | 7.1 | 6.49 | 0.073 | 0.004 | 0.412 | 0.143 | 'VAMP7 |
| 209699_x_at | 11.11 | 11.23 | 9.74 | 0.002 | 0 | 0.014 | 0 | 'AKR1C2 |
| 218002_s_at | 6.24 | 6.42 | 4.9 | 0.103 | 0.008 | 0.146 | 0.017 | 'CXCL14 |
| 214773_x_at | 5.97 | 5.7 | 4.82 | 0.123 | 0.011 | 0.071 | 0.004 | 'TIPRL |
| 209063_x_at | 7.08 | 7.2 | 6.62 | 0.043 | 0.001 | 0.088 | 0.006 | 'PAIP1 |
| 202679_at | 6.29 | 6.48 | 6.7 | 0.41 | 0.139 | 0.104 | 0.009 | 'NPC1 |
| 220199_s_at | 4.79 | 5.22 | 4.49 | 0.063 | 0.003 | 0.667 | 0.407 | 'AIDA |
| 212542_s_at | 7.6 | 7.37 | 8.04 | 0.038 | 0.001 | 0.135 | 0.015 | 'PHIP |
| 209314_s_at | 4.3 | 3.56 | 2.59 | 0.296 | 0.068 | 0.075 | 0.004 | 'HBS1L |
| 218920_at | 7.09 | 7.26 | 7.86 | 0.033 | 0.001 | 0.038 | 0.001 | 'FLJ10404 |
| 205990_s_at | 6.07 | 6 | 5.33 | 0.124 | 0.011 | 0.093 | 0.007 | 'WNT5A |
| 212411_at | 7.85 | 7.66 | 8.13 | 0.081 | 0.005 | 0.246 | 0.05 | 'IMP4 |
| 203569_s_at | 8.91 | 8.64 | 9.36 | 0.007 | 0 | 0.115 | 0.011 | 'OFD1 |
| 221756_at | 7.55 | 7.44 | 7.92 | 0.083 | 0.005 | 0.215 | 0.039 | 'PIK3IP1 |
| 207222_at | 7.84 | 7.67 | 7.07 | 0.133 | 0.014 | 0.057 | 0.002 | 'PLA2G10 |
| 209309_at | 6.29 | 5.89 | 4.98 | 0.097 | 0.007 | 0.038 | 0.001 | 'AZGP1 |
| 208977_x_at | 12.06 | 12.24 | 12.62 | 0.237 | 0.043 | 0.084 | 0.006 | 'TUBB2C |
| 203906_at | 7.03 | 6.99 | 7.71 | 0.025 | 0 | 0.038 | 0.001 | 'IQSEC1 |
| 209118_s_at | 12.4 | 12 | 11.56 | 0.335 | 0.089 | 0.089 | 0.006 | 'TUBA1A |
| 201098_at | 8.29 | 8.45 | 7.9 | 0.013 | 0 | 0.082 | 0.005 | 'COPB2 |
| 217836_s_at | 6.64 | 6.76 | 7.34 | 0.029 | 0.001 | 0.116 | 0.011 | 'YY1AP1 |
| 205725_at | 12.01 | 12.02 | 14.33 | 0.061 | 0.003 | 0.059 | 0.003 | 'SCGB1A1 |
| 221887_s_at | 5.65 | 5.88 | 6.85 | 0.018 | 0 | 0.006 | 0 | 'DFNB31 |
| 208979_at | 7.08 | 7.18 | 7.56 | 0.129 | 0.012 | 0.049 | 0.002 | 'NCOA6 |
| 204383_at | 5.44 | 5.37 | 6.2 | 0.03 | 0.001 | 0.051 | 0.002 | 'DGCR14 |
| 200884_at | 8.95 | 8.56 | 9.67 | 0.007 | 0 | 0.075 | 0.004 | 'CKB |
| 212807_s_at | 7.04 | 6.86 | 6.52 | 0.077 | 0.004 | 0.014 | 0 | 'SORT1 |
| 202197_at | 7.65 | 7.72 | 7.92 | 0.217 | 0.036 | 0.102 | 0.008 | 'MTMR3 |
| 209234_at | 5.49 | 6.05 | 6.65 | 0.09 | 0.006 | 0.035 | 0.001 | 'KIF1B |
| 218312_s_at | 8.18 | 8.22 | 8.65 | 0.047 | 0.002 | 0.067 | 0.003 | 'ZSCAN18 |
| 32402_s_at | 6.08 | 5.5 | 6.24 | 0.104 | 0.008 | 0.76 | 0.54 | 'SYMPK |
| 211750_x_at | 12.09 | 12.19 | 12.45 | 0.235 | 0.042 | 0.101 | 0.008 | 'TUBA1C |
| 214327_x_at | 11.61 | 11.49 | 12.55 | 0.09 | 0.006 | 0.124 | 0.013 | 'TPT1 |
| 215222_x_at | 6.34 | 6.99 | 7.21 | 0.633 | 0.354 | 0.073 | 0.004 | 'MACF1 |
| 202842_s_at | 8.09 | 8.13 | 7.4 | 0.092 | 0.007 | 0.104 | 0.009 | 'DNAJB9 |
| 202796_at | 5.57 | 5.49 | 6.96 | 0.02 | 0 | 0.032 | 0.001 | 'SYNPO |
| 205267_at | 5.46 | 5.29 | 6.13 | 0.058 | 0.003 | 0.189 | 0.03 | 'POU2AF1 |
| 219747_at | 6.08 | 5.78 | 6.71 | 0.021 | 0 | 0.1 | 0.008 | 'C4orf31 |
| 208614_s_at | 8.04 | 8.19 | 8.99 | 0.002 | 0 | 0.025 | 0 | 'FLNB |
| 210466_s_at | 8.68 | 8.26 | 7.59 | 0.169 | 0.021 | 0.045 | 0.001 | 'SERBP1 |
| 213455_at | 8.86 | 9.02 | 8.3 | 0.022 | 0 | 0.086 | 0.006 | 'FAM114A1 |
| 212445_s_at | 6.99 | 7.18 | 7.38 | 0.29 | 0.065 | 0.096 | 0.008 | 'NEDD4L |
| 218972_at | 6.74 | 6.62 | 6.21 | 0.23 | 0.04 | 0.099 | 0.008 | 'TTC17 |
| 218651_s_at | 6.91 | 6.74 | 7.27 | 0.038 | 0.001 | 0.17 | 0.024 | 'LARP6 |
| 219041_s_at | 7.72 | 7.74 | 8.17 | 0.149 | 0.017 | 0.094 | 0.007 | 'REPIN1 |
| 213018_at | 6.08 | 6.36 | 6.64 | 0.339 | 0.091 | 0.07 | 0.004 | 'GATAD1 |
| 207543_s_at | 7.8 | 7.6 | 7.2 | 0.255 | 0.05 | 0.101 | 0.008 | 'P4HA1 |
| 207722_s_at | 5.38 | 5.23 | 6.59 | 0.043 | 0.001 | 0.096 | 0.008 | 'BTBD2 |
| 200982_s_at | 5.58 | 6.04 | 6.43 | 0.336 | 0.09 | 0.053 | 0.002 | 'ANXA6 |
| 200801_x_at | 11.95 | 12.22 | 12.5 | 0.358 | 0.104 | 0.041 | 0.001 | 'ACTB |
| 218175_at | 6.92 | 7.18 | 8.02 | 0.042 | 0.001 | 0.042 | 0.001 | 'CCDC92 |
| 201997_s_at | 7.42 | 7.46 | 7.93 | 0.059 | 0.003 | 0.048 | 0.002 | 'SPEN |
| 201775_s_at | 6.97 | 7.11 | 6.58 | 0.049 | 0.002 | 0.12 | 0.012 | 'KIAA0494 |
| 208692_at | 11.96 | 11.74 | 12.51 | 0.024 | 0 | 0.103 | 0.009 | 'RPS3 |
| 215346_at | 5.11 | 5.69 | 6.28 | 0.206 | 0.032 | 0.031 | 0 | 'CD40 |
| 200596_s_at | 5.62 | 6.31 | 7.55 | 0.178 | 0.024 | 0.049 | 0.002 | 'EIF3A |
| 89476_r_at | 5.92 | 6.09 | 7.07 | 0.087 | 0.006 | 0.051 | 0.002 | 'NPEPL1 |
| 35685_at | 8.46 | 8.35 | 8.74 | 0.033 | 0.001 | 0.179 | 0.026 | 'RING1 |
| 214585_s_at | 7.99 | 7.9 | 8.44 | 0.095 | 0.007 | 0.179 | 0.026 | 'VPS52 |
| 202290_at | 6.2 | 6.77 | 7.17 | 0.444 | 0.166 | 0.061 | 0.003 | 'PDAP1 |
| 208919_s_at | 7.34 | 7.76 | 7.26 | 0.042 | 0.001 | 0.828 | 0.656 | 'NADK |
| 78383_at | 6.56 | 6.54 | 6.91 | 0.081 | 0.005 | 0.136 | 0.015 | 'TOPORS |
| 211970_x_at | 12.12 | 12.25 | 12.63 | 0.127 | 0.012 | 0.099 | 0.008 | 'ACTG1 |
| 209386_at | 7.47 | 7.47 | 6.11 | 0.044 | 0.002 | 0.045 | 0.001 | 'TM4SF1 |
| 200655_s_at | 11.04 | 10.73 | 10.52 | 0.408 | 0.138 | 0.068 | 0.003 | 'CALM1 |
| 203113_s_at | 10.14 | 9.74 | 10.36 | 0.041 | 0.001 | 0.566 | 0.285 | 'EEF1D |
| 218424_s_at | 8.53 | 8.13 | 8.74 | 0.099 | 0.007 | 0.57 | 0.29 | 'STEAP3 |
| 217760_at | 7.56 | 7.69 | 8.05 | 0.058 | 0.003 | 0.025 | 0 | 'TRIM44 |
| 202029_x_at | 11.83 | 11.83 | 12.52 | 0.109 | 0.009 | 0.101 | 0.008 | 'RPL38 |
| 209688_s_at | 5.84 | 6.19 | 6.71 | 0.032 | 0.001 | 0.026 | 0 | 'CCDC93 |
| 218556_at | 8.83 | 8.77 | 8.34 | 0.178 | 0.024 | 0.103 | 0.009 | 'ORMDL2 |
| 220351_at | 4.9 | 4.97 | 6.12 | 0.024 | 0 | 0.024 | 0 | 'CCRL1 |
| 221546_at | 3.88 | 4.57 | 5.41 | 0.128 | 0.012 | 0.02 | 0 | 'PRPF18 |
| 201397_at | 6.44 | 6.59 | 7.14 | 0.046 | 0.002 | 0.028 | 0 | 'PHGDH |
| 203240_at | 8.73 | 8.76 | 11.29 | 0.004 | 0 | 0.006 | 0 | 'FCGBP |
| 201578_at | 7.06 | 7.22 | 7.7 | 0.142 | 0.015 | 0.064 | 0.003 | 'PODXL |
| 201463_s_at | 9.83 | 10.09 | 9.34 | 0.017 | 0 | 0.139 | 0.015 | 'TALDO1 |
| 205759_s_at | 6.65 | 6.48 | 6.98 | 0.05 | 0.002 | 0.234 | 0.046 | 'SULT2B1 |
| 201030_x_at | 10.58 | 10.54 | 11.33 | 0.007 | 0 | 0.018 | 0 | 'LDHB |
| 208712_at | 7.57 | 7.36 | 7.95 | 0.047 | 0.002 | 0.309 | 0.079 | 'CCND1 |
| 201820_at | 10.05 | 9.9 | 10.82 | 0.007 | 0 | 0.073 | 0.004 | 'KRT5 |
| 213796_at | 4.11 | 4.32 | 1.98 | 0.013 | 0 | 0.066 | 0.003 | 'SPRR1A |
| 216316_x_at | 4.88 | 5.1 | 3.36 | 0.038 | 0.001 | 0.066 | 0.003 | 'GK|GK3P |
| 201672_s_at | 7.98 | 8.04 | 7.73 | 0.097 | 0.007 | 0.14 | 0.016 | 'USP14 |
| 204542_at | 8.09 | 8.15 | 8.95 | 0.042 | 0.001 | 0.032 | 0.001 | 'ST6GALNAC2 |
| 200872_at | 9.13 | 9.49 | 8.69 | 0.017 | 0 | 0.124 | 0.013 | 'S100A10 |
| 216520_s_at | 11.79 | 11.99 | 12.68 | 0.316 | 0.079 | 0.062 | 0.003 | 'TPT1 |
| 212221_x_at | 8.35 | 8.17 | 7.18 | 0.16 | 0.019 | 0.1 | 0.008 | 'IDS |
| 203135_at | 5.89 | 6.07 | 6.78 | 0.106 | 0.008 | 0.053 | 0.002 | 'TBP |
| 212312_at | 8.2 | 7.82 | 7.71 | 0.753 | 0.523 | 0.081 | 0.005 | 'BCL2L1 |
| 203380_x_at | 8.84 | 8.84 | 9.25 | 0.018 | 0 | 0.047 | 0.002 | 'SFRS5 |
| 214882_s_at | 6.7 | 7.34 | 7.63 | 0.581 | 0.297 | 0.078 | 0.005 | 'SFRS2 |
| 203244_at | 7.29 | 7.26 | 7.66 | 0.072 | 0.004 | 0.093 | 0.007 | 'PEX5 |
| 221046_s_at | 8.28 | 7.8 | 7.6 | 0.586 | 0.302 | 0.057 | 0.002 | 'GTPBP8 |
| 201942_s_at | 4.04 | 5.11 | 3.35 | 0.041 | 0.001 | 0.468 | 0.189 | 'CPD |
| 211778_s_at | 7.8 | 7.41 | 7.75 | 0.042 | 0.001 | 0.839 | 0.675 | 'OVOL2 |
| 200675_at | 10.2 | 10.02 | 10.64 | 0.005 | 0 | 0.102 | 0.009 | 'CD81 |
| 217730_at | 7.39 | 7.68 | 7.96 | 0.39 | 0.125 | 0.064 | 0.003 | 'TMBIM1 |
| 204803_s_at | 8.36 | 8.44 | 9.25 | 0.045 | 0.002 | 0.078 | 0.005 | 'RRAD |
| 201910_at | 6.82 | 7.09 | 7.47 | 0.192 | 0.028 | 0.047 | 0.001 | 'FARP1 |
| 221607_x_at | 12.03 | 12.22 | 12.53 | 0.288 | 0.064 | 0.101 | 0.008 | 'ACTG1 |
| 38964_r_at | 8.35 | 7.85 | 7.75 | 0.813 | 0.62 | 0.086 | 0.006 | 'WAS |
| 204990_s_at | 7.72 | 7.94 | 8.8 | 0.018 | 0 | 0.06 | 0.003 | 'ITGB4 |
| 212156_at | 6.03 | 6.36 | 7.42 | 0.059 | 0.003 | 0.032 | 0.001 | 'VPS39 |
| 320_at | 6.71 | 6.44 | 7.15 | 0.071 | 0.004 | 0.267 | 0.059 | 'PEX6 |
| 204532_x_at | 8.22 | 8.44 | 7.8 | 0.021 | 0 | 0.187 | 0.029 | 'UGT1A4 |
| 204716_at | 6.19 | 5.94 | 5.28 | 0.155 | 0.018 | 0.043 | 0.001 | 'CCDC6 |
| 211653_x_at | 10.57 | 10.74 | 9.37 | 0.022 | 0 | 0.055 | 0.002 | 'AKR1C2 |
| 212059_s_at | 6.2 | 6.33 | 6.8 | 0.114 | 0.01 | 0.035 | 0.001 | 'TRPC4AP |
| 213792_s_at | 7.57 | 7.65 | 8.59 | 0.028 | 0.001 | 0.028 | 0 | 'INSR |
| 220166_at | 4.53 | 4.86 | 5.59 | 0.161 | 0.019 | 0.044 | 0.001 | 'CNNM1 |
| 222175_s_at | 6.45 | 7.02 | 7.54 | 0.109 | 0.009 | 0.021 | 0 | 'MED15 |
| 205640_at | 9.13 | 9.07 | 9.9 | 0.004 | 0 | 0.025 | 0 | 'ALDH3B1 |
| 219724_s_at | 4.38 | 4.13 | 5.39 | 0.09 | 0.006 | 0.164 | 0.022 | 'KIAA0748 |
| 217902_s_at | 5.64 | 5.9 | 6.25 | 0.141 | 0.015 | 0.043 | 0.001 | 'HERC2 |
| 218677_at | 8.43 | 8.47 | 7.95 | 0.032 | 0.001 | 0.062 | 0.003 | 'S100A14 |
| 213867_x_at | 12.05 | 12.39 | 12.67 | 0.31 | 0.076 | 0.041 | 0.001 | 'ACTB |
| 214909_s_at | 5.98 | 6.11 | 6.94 | 0.149 | 0.017 | 0.103 | 0.009 | 'DDAH2 |
| 211404_s_at | 7.39 | 7.6 | 6.93 | 0.021 | 0 | 0.131 | 0.014 | 'APLP2 |
| 200039_s_at | 8.84 | 8.89 | 8.6 | 0.026 | 0.001 | 0.131 | 0.014 | 'PSMB2 |
| 216232_s_at | 5.59 | 5.76 | 6.43 | 0.175 | 0.023 | 0.047 | 0.001 | 'GCN1L1 |
| 216594_x_at | 11.04 | 11.14 | 9.63 | 0.002 | 0 | 0.013 | 0 | 'AKR1C1 |
| 219080_s_at | 5.91 | 5.82 | 5.5 | 0.13 | 0.013 | 0.06 | 0.003 | 'CTPS2 |
| 200949_x_at | 11.19 | 11.31 | 11.95 | 0.08 | 0.005 | 0.045 | 0.001 | 'RPS20 |
| 209134_s_at | 12.12 | 11.75 | 12.33 | 0.074 | 0.004 | 0.589 | 0.312 | 'RPS6 |
| 212232_at | 6.58 | 7.09 | 7.33 | 0.401 | 0.132 | 0.023 | 0 | 'FNBP4 |
| 201588_at | 9.47 | 9.38 | 8.92 | 0.033 | 0.001 | 0.022 | 0 | 'TXNL1 |
| 213571_s_at | 7.61 | 7.37 | 6.81 | 0.086 | 0.006 | 0.054 | 0.002 | 'EIF4E2 |
| 213274_s_at | 8.19 | 8.2 | 7.81 | 0.026 | 0.001 | 0.046 | 0.001 | 'CTSB |
| 209719_x_at | 11.28 | 11.59 | 12.3 | 0.058 | 0.003 | 0.027 | 0 | 'SERPINB3 |
| 219627_at | 6.03 | 6.18 | 6.53 | 0.248 | 0.048 | 0.083 | 0.005 | 'ZNF767 |
| 201080_at | 6.62 | 6.62 | 6.99 | 0.118 | 0.01 | 0.099 | 0.008 | 'PIP4K2B |
| 202831_at | 9.35 | 9.69 | 8.02 | 0 | 0 | 0.003 | 0 | 'GPX2 |
| 209442_x_at | 7.32 | 7.61 | 8.08 | 0.204 | 0.032 | 0.058 | 0.002 | 'ANK3 |
| 200817_x_at | 12.58 | 12.45 | 12.93 | 0.093 | 0.007 | 0.202 | 0.034 | 'RPS10 |
| 204735_at | 7.2 | 6.92 | 7.49 | 0.013 | 0 | 0.202 | 0.034 | 'PDE4A |
| 211990_at | 9.57 | 9.92 | 10.54 | 0.182 | 0.026 | 0.026 | 0 | 'HLA-DPA1 |
| 200933_x_at | 12.06 | 12.08 | 12.74 | 0.04 | 0.001 | 0.039 | 0.001 | 'RPS4X |
| 214736_s_at | 7.51 | 7.7 | 8.56 | 0.007 | 0 | 0.006 | 0 | 'ADD1 |
| 212988_x_at | 11.96 | 12.1 | 12.49 | 0.177 | 0.024 | 0.094 | 0.007 | 'ACTG1 |
| 218845_at | 8.49 | 8.15 | 7.78 | 0.142 | 0.015 | 0.027 | 0 | 'DUSP22 |
| 205372_at | 5.41 | 4.53 | 5.92 | 0.029 | 0.001 | 0.348 | 0.102 | 'PLAG1 |
| 208714_at | 9.01 | 8.98 | 9.48 | 0.075 | 0.004 | 0.108 | 0.01 | 'NDUFV1 |
| 204326_x_at | 9.87 | 9.49 | 10.4 | 0.008 | 0 | 0.111 | 0.01 | 'MT1X |
| 218599_at | 7.99 | 8.15 | 8.45 | 0.075 | 0.004 | 0.045 | 0.001 | 'REC8 |
| 64942_at | 8.28 | 8.04 | 7.4 | 0.073 | 0.004 | 0.035 | 0.001 | 'GPR153 |
| 206825_at | 7.38 | 7.09 | 8.31 | 0.05 | 0.002 | 0.136 | 0.015 | 'OXTR |
| 214742_at | 5.03 | 5.05 | 6.01 | 0.107 | 0.008 | 0.072 | 0.004 | 'AZI1 |
| 221829_s_at | 10.43 | 9.75 | 9.35 | 0.407 | 0.138 | 0.046 | 0.001 | 'TNPO1 |
| 200663_at | 11.4 | 11.27 | 11.1 | 0.355 | 0.102 | 0.079 | 0.005 | 'CD63 |
| 209523_at | 6.17 | 6.25 | 5.67 | 0.052 | 0.002 | 0.08 | 0.005 | 'TAF2 |
| 41047_at | 8.82 | 8.33 | 7.99 | 0.17 | 0.022 | 0.009 | 0 | 'C9orf16 |
| 200953_s_at | 7.19 | 6.94 | 7.7 | 0.018 | 0 | 0.111 | 0.01 | 'CCND2 |
| 203655_at | 5.88 | 5.78 | 6.44 | 0.073 | 0.004 | 0.196 | 0.032 | 'XRCC1 |
| 203236_s_at | 6.77 | 7.19 | 7.48 | 0.443 | 0.166 | 0.08 | 0.005 | 'LGALS9 |
| 218346_s_at | 7.14 | 7.08 | 7.67 | 0.002 | 0 | 0.102 | 0.008 | 'SESN1 |
| 218868_at | 4.97 | 5.09 | 5.91 | 0.004 | 0 | 0.02 | 0 | 'ACTR3B |
| 213471_at | 6.14 | 6.19 | 6.66 | 0.011 | 0 | 0.071 | 0.004 | 'NPHP4 |
| 1598_g_at | 8.47 | 8.35 | 8.99 | 0.053 | 0.002 | 0.091 | 0.007 | 'GAS6 |
| 212931_at | 7.78 | 7.77 | 8.58 | 0.082 | 0.005 | 0.082 | 0.005 | 'TCF20 |
| 214548_x_at | 10.41 | 10.76 | 11.47 | 0.136 | 0.014 | 0.047 | 0.002 | 'GNAS |
| 44783_s_at | 10.05 | 9.32 | 9.86 | 0.09 | 0.006 | 0.646 | 0.382 | 'HEY1 |
| 210276_s_at | 6.77 | 7.05 | 7.44 | 0.296 | 0.068 | 0.047 | 0.001 | 'TRIOBP |
| 204287_at | 6.32 | 6.12 | 6.77 | 0.051 | 0.002 | 0.153 | 0.019 | 'SYNGR1 |
| 212361_s_at | 7.22 | 7.6 | 8.36 | 0.092 | 0.006 | 0.043 | 0.001 | 'ATP2A2 |
| 200809_x_at | 11.82 | 11.97 | 12.71 | 0.072 | 0.004 | 0.038 | 0.001 | 'RPL12 |
| 209789_at | 5.8 | 5.24 | 6.42 | 0.07 | 0.004 | 0.273 | 0.061 | 'CORO2B |
| 202308_at | 7.27 | 7.05 | 7.74 | 0.074 | 0.004 | 0.202 | 0.034 | 'SREBF1 |
| 205022_s_at | 6.6 | 6.73 | 7.58 | 0.01 | 0 | 0.09 | 0.007 | 'FOXN3 |
| 213359_at | 5.22 | 6.06 | 6.25 | 0.683 | 0.42 | 0.062 | 0.003 | 'HNRNPD |
| 212852_s_at | 8.17 | 8.32 | 8.68 | 0.14 | 0.015 | 0.098 | 0.008 | 'TROVE2 |
| 201331_s_at | 9.1 | 8.96 | 9.48 | 0.041 | 0.001 | 0.114 | 0.011 | 'STAT6 |
| 211991_s_at | 7.69 | 8.88 | 9.23 | 0.618 | 0.336 | 0.032 | 0.001 | 'HLA-DPA1 |
| 202746_at | 6 | 5.32 | 6.41 | 0.04 | 0.001 | 0.429 | 0.157 | 'ITM2A |
| 204295_at | 9.01 | 8.66 | 8.43 | 0.418 | 0.146 | 0.059 | 0.003 | 'SURF1 |
| 221850_x_at | 7.36 | 7.47 | 8.08 | 0.067 | 0.003 | 0.038 | 0.001 | 'CTGLF1 |
| 211975_at | 8.47 | 8.2 | 8.66 | 0.083 | 0.005 | 0.514 | 0.232 | 'ARFGAP2 |
| 201173_x_at | 9.89 | 9.68 | 10.12 | 0.03 | 0.001 | 0.332 | 0.093 | 'NUDC |
| 212537_x_at | 11.23 | 11.24 | 11.84 | 0.059 | 0.003 | 0.066 | 0.003 | 'RPL17 |
| 211208_s_at | 7.63 | 7.5 | 7.08 | 0.045 | 0.002 | 0.043 | 0.001 | 'CASK |
| 200754_x_at | 8.7 | 9.32 | 9.91 | 0.182 | 0.025 | 0.026 | 0 | 'SFRS2 |
| 218456_at | 6.38 | 6.17 | 6.88 | 0.067 | 0.003 | 0.18 | 0.026 | 'CAPRIN2 |
| 203278_s_at | 5.57 | 5.59 | 6.57 | 0.079 | 0.005 | 0.076 | 0.004 | 'PHF21A |
| 220417_s_at | 7.08 | 7.3 | 7.81 | 0.145 | 0.016 | 0.04 | 0.001 | 'THAP4|LOC728944 |
| 200612_s_at | 7.64 | 7.86 | 7.13 | 0.01 | 0 | 0.051 | 0.002 | 'AP2B1 |
| 219675_s_at | 6.3 | 6.68 | 6.14 | 0.08 | 0.005 | 0.707 | 0.46 | 'UXS1 |
| 202713_s_at | 8.01 | 7.71 | 7.41 | 0.313 | 0.077 | 0.037 | 0.001 | 'KIAA0391 |
| 211855_s_at | 4.7 | 5.34 | 5.78 | 0.304 | 0.072 | 0.064 | 0.003 | 'SLC25A14 |
| 200082_s_at | 10.37 | 10.49 | 11.21 | 0.118 | 0.01 | 0.065 | 0.003 | 'RPS7|LOC644315 |
| 201480_s_at | 7.5 | 7.46 | 8.03 | 0.098 | 0.007 | 0.123 | 0.013 | 'SUPT5H |
| 200732_s_at | 7.82 | 7.83 | 7.28 | 0.051 | 0.002 | 0.067 | 0.003 | 'PTP4A1 |
| 205870_at | 6.48 | 6.64 | 7.24 | 0.07 | 0.004 | 0.096 | 0.007 | 'BDKRB2 |
| 201125_s_at | 7.92 | 7.63 | 8.24 | 0.002 | 0 | 0.116 | 0.011 | 'ITGB5 |
| 203713_s_at | 6.89 | 6.87 | 7.47 | 0.038 | 0.001 | 0.047 | 0.002 | 'LLGL2 |
| 211996_s_at | 7.91 | 8.61 | 9.46 | 0.165 | 0.02 | 0.024 | 0 | 'LOC23117 |
| 210907_s_at | 8.58 | 8.52 | 7.75 | 0.078 | 0.005 | 0.057 | 0.002 | 'PDCD10 |
| 214924_s_at | 9.72 | 9.56 | 9.95 | 0.03 | 0.001 | 0.317 | 0.083 | 'TRAK1 |
| 209312_x_at | 10.54 | 11.26 | 11.6 | 0.551 | 0.266 | 0.059 | 0.002 | 'HLA-DRB1 |
| 210064_s_at | 8.73 | 8.99 | 7.74 | 0.023 | 0 | 0.063 | 0.003 | 'UPK1B |
| 212109_at | 7.87 | 7.45 | 8.05 | 0.045 | 0.002 | 0.536 | 0.255 | 'HN1L |
| 208718_at | 10.71 | 10.84 | 11.25 | 0.086 | 0.006 | 0.056 | 0.002 | 'DDX17 |
| 203757_s_at | 9.94 | 10.64 | 9.65 | 0.027 | 0.001 | 0.605 | 0.328 | 'CEACAM6 |
| 211799_x_at | 9.02 | 9.61 | 10.14 | 0.402 | 0.134 | 0.032 | 0.001 | 'HLA-C |
| 211564_s_at | 7.71 | 7.38 | 8.4 | 0.045 | 0.002 | 0.157 | 0.02 | 'PDLIM4 |
| 205807_s_at | 7.57 | 7.36 | 6.86 | 0.053 | 0.002 | 0.01 | 0 | 'TUFT1 |
| 211383_s_at | 7.18 | 6.98 | 7.36 | 0.059 | 0.003 | 0.291 | 0.069 | 'WDR37 |
| 218040_at | 5.1 | 5.05 | 5.82 | 0.074 | 0.004 | 0.107 | 0.009 | 'PRPF38B |
| 212716_s_at | 9.55 | 9.3 | 9.72 | 0.029 | 0.001 | 0.463 | 0.185 | 'EIF3K |
| 204740_at | 7.32 | 7.09 | 7.73 | 0.026 | 0.001 | 0.122 | 0.012 | 'CNKSR1 |
| 214580_x_at | 4.8 | 5.46 | 3.21 | 0.018 | 0 | 0.14 | 0.016 | 'KRT6C|KRT6A|KRT6B |
| 204970_s_at | 5.36 | 6.21 | 6.09 | 0.786 | 0.575 | 0.096 | 0.008 | 'MAFG|LOC644132 |
| 217739_s_at | 6.32 | 6.72 | 5.69 | 0.019 | 0 | 0.147 | 0.017 | 'NAMPT |
| 202262_x_at | 6.8 | 6.55 | 7.68 | 0.025 | 0 | 0.074 | 0.004 | 'DDAH2 |
| 201266_at | 8.8 | 9.3 | 8.28 | 0.04 | 0.001 | 0.285 | 0.067 | 'TXNRD1 |
| 200635_s_at | 8.01 | 7.96 | 8.55 | 0.092 | 0.006 | 0.124 | 0.013 | 'PTPRF |
| 208606_s_at | 5.2 | 4.86 | 6.07 | 0.029 | 0.001 | 0.06 | 0.003 | 'WNT4 |
| 35148_at | 7.76 | 7.62 | 8.37 | 0.001 | 0 | 0.043 | 0.001 | 'TJP3 |
| 208611_s_at | 7.76 | 7.8 | 8.53 | 0.055 | 0.002 | 0.065 | 0.003 | 'SPTAN1 |
| 213564_x_at | 10.95 | 10.73 | 11.18 | 0.011 | 0 | 0.345 | 0.101 | 'LDHB |
| 201467_s_at | 8.17 | 8.62 | 6.64 | 0.054 | 0.002 | 0.121 | 0.012 | 'NQO1 |
| 219357_at | 7.66 | 7.36 | 7.9 | 0.087 | 0.006 | 0.574 | 0.294 | 'GTPBP1 |
| 211956_s_at | 11.19 | 11.23 | 11.57 | 0.138 | 0.014 | 0.1 | 0.008 | 'EIF1 |
| 221970_s_at | 7.19 | 7.16 | 7.54 | 0.104 | 0.008 | 0.136 | 0.015 | 'NOL11 |
| 200094_s_at | 10.17 | 10.27 | 10.61 | 0.092 | 0.006 | 0.071 | 0.004 | 'EEF2 |
| 210212_x_at | 6.7 | 6.68 | 7.19 | 0.091 | 0.006 | 0.209 | 0.036 | 'MTCP1 |
| 200980_s_at | 7.81 | 7.93 | 8.28 | 0.103 | 0.008 | 0.04 | 0.001 | 'PDHA1 |
| 203900_at | 4.76 | 5.18 | 5.57 | 0.351 | 0.099 | 0.061 | 0.003 | 'KIAA0467 |
| 221241_s_at | 7.51 | 7.22 | 6.59 | 0.222 | 0.038 | 0.038 | 0.001 | 'BCL2L14 |
| 210982_s_at | 8.5 | 10.03 | 10.21 | 0.848 | 0.68 | 0.089 | 0.006 | 'HLA-DRA |
| 204690_at | 8.13 | 7.82 | 7.79 | 0.911 | 0.807 | 0.101 | 0.008 | 'STX8 |
| 215157_x_at | 10.56 | 10.72 | 11.32 | 0.206 | 0.033 | 0.086 | 0.006 | 'PABPC1 |
| 200789_at | 7.56 | 7.84 | 8.45 | 0.041 | 0.001 | 0.035 | 0.001 | 'ECH1 |
| 202116_at | 6.51 | 6.28 | 7.11 | 0.027 | 0.001 | 0.076 | 0.004 | 'DPF2 |
| 213075_at | 6.43 | 6.42 | 7.08 | 0.061 | 0.003 | 0.056 | 0.002 | 'OLFML2A |
| 211965_at | 6.48 | 6.04 | 7.01 | 0.069 | 0.004 | 0.189 | 0.029 | 'ZFP36L1 |
| 202449_s_at | 8.03 | 7.75 | 8.33 | 0.027 | 0.001 | 0.225 | 0.043 | 'RXRA |
| 823_at | 7.56 | 7.44 | 8.22 | 0.002 | 0 | 0.009 | 0 | 'CX3CL1 |
| 222146_s_at | 5.47 | 5.93 | 6.6 | 0.108 | 0.009 | 0.043 | 0.001 | 'TCF4 |
| 212365_at | 7.55 | 7.8 | 7.14 | 0.007 | 0 | 0.102 | 0.009 | 'MYO1B |
| 208894_at | 9.13 | 10.43 | 10.56 | 0.85 | 0.686 | 0.047 | 0.002 | 'HLA-DRA |
| 202135_s_at | 7.82 | 8.06 | 8.31 | 0.2 | 0.03 | 0.035 | 0.001 | 'ACTR1B |
| 203359_s_at | 8.68 | 8.41 | 7.8 | 0.236 | 0.042 | 0.096 | 0.007 | 'MYCBP |
| 201717_at | 8.86 | 8.84 | 9.11 | 0.096 | 0.007 | 0.208 | 0.036 | 'MRPL49 |
| 220798_x_at | 6.41 | 5.86 | 6.99 | 0.022 | 0 | 0.271 | 0.06 | 'PRG2 |
| 218193_s_at | 5.96 | 5.95 | 5.11 | 0.058 | 0.003 | 0.059 | 0.003 | 'GOLT1B |
| 209529_at | 8.55 | 8.85 | 9.4 | 0.135 | 0.014 | 0.045 | 0.001 | 'PPAP2C |
| 205384_at | 6.95 | 6.49 | 7.6 | 0.066 | 0.003 | 0.307 | 0.078 | 'FXYD1 |
| 209154_at | 9.12 | 8.76 | 8.08 | 0.239 | 0.043 | 0.075 | 0.004 | 'TAX1BP3 |
| 205244_s_at | 4.68 | 4.33 | 6.03 | 0.081 | 0.005 | 0.157 | 0.02 | 'SLC13A3 |
| 207180_s_at | 6.85 | 7.15 | 6.43 | 0.033 | 0.001 | 0.193 | 0.031 | 'HTATIP2 |
| 210076_x_at | 7.19 | 7.04 | 6.3 | 0.154 | 0.018 | 0.094 | 0.007 | 'SERBP1 |
| 200062_s_at | 11.88 | 11.94 | 12.61 | 0.067 | 0.003 | 0.07 | 0.004 | 'RPL30 |
| 203006_at | 7.16 | 6.91 | 6.73 | 0.465 | 0.184 | 0.023 | 0 | 'INPP5A |
| 213530_at | 5.44 | 5.67 | 6.15 | 0.106 | 0.008 | 0.075 | 0.004 | 'RAB3GAP1 |
| 219876_s_at | 5.76 | 5.65 | 6.73 | 0.026 | 0.001 | 0.035 | 0.001 | 'GOLGA2L1 |
| 220408_x_at | 6.73 | 7.09 | 7.1 | 0.961 | 0.91 | 0.096 | 0.007 | 'FAM48A |
| 201357_s_at | 6.53 | 6.63 | 7.02 | 0.163 | 0.02 | 0.078 | 0.005 | 'SF3A1 |
| 205043_at | 5.38 | 5.48 | 6.63 | 0.04 | 0.001 | 0.02 | 0 | 'CFTR |
| 220547_s_at | 8.25 | 7.92 | 7.77 | 0.631 | 0.351 | 0.081 | 0.005 | 'FAM35A |
| 200741_s_at | 12.11 | 12.04 | 12.62 | 0.018 | 0 | 0.04 | 0.001 | 'RPS27 |
| 207761_s_at | 9.61 | 9.64 | 10.48 | 0.002 | 0 | 0.018 | 0 | 'METTL7A |
| 210966_x_at | 8.33 | 8.39 | 8.83 | 0.068 | 0.003 | 0.04 | 0.001 | 'LARP1 |
| 204580_at | 5.26 | 5.63 | 4.87 | 0.101 | 0.008 | 0.481 | 0.199 | 'MMP12 |
| 214746_s_at | 6.52 | 6.87 | 6.17 | 0.097 | 0.007 | 0.448 | 0.173 | 'ZNF467 |
| 208807_s_at | 8.16 | 7.86 | 8.33 | 0.032 | 0.001 | 0.47 | 0.191 | 'CHD3 |
| 207842_s_at | 7.45 | 7.32 | 7.89 | 0.046 | 0.002 | 0.134 | 0.015 | 'CASC3 |
| 218394_at | 8.31 | 8.15 | 8.49 | 0.074 | 0.004 | 0.405 | 0.137 | 'ROGDI |
| 219956_at | 8.7 | 8.53 | 7.76 | 0.116 | 0.01 | 0.066 | 0.003 | 'GALNT6 |
| 205405_at | 6.98 | 6.53 | 7.39 | 0.023 | 0 | 0.188 | 0.029 | 'SEMA5A |
| 210951_x_at | 7.51 | 7.3 | 6.36 | 0.173 | 0.022 | 0.09 | 0.007 | 'RAB27A |
| 216526_x_at | 12.35 | 12.57 | 13.16 | 0.097 | 0.007 | 0.03 | 0 | 'HLA-C |
| 203688_at | 4.65 | 5 | 5.78 | 0.101 | 0.008 | 0.079 | 0.005 | 'PKD2 |
| 222251_s_at | 6.56 | 6.4 | 6.75 | 0.055 | 0.002 | 0.384 | 0.125 | 'GMEB2 |
| 200086_s_at | 9.85 | 9.51 | 9.47 | 0.854 | 0.692 | 0.047 | 0.001 | 'COX4I1 |
| 221964_at | 5.98 | 6.14 | 6.91 | 0.05 | 0.002 | 0.059 | 0.003 | 'TULP3 |
| 220562_at | 3.25 | 3.69 | 5 | 0.08 | 0.005 | 0.038 | 0.001 | 'CYP2W1 |
| 218505_at | 6.54 | 6.89 | 7.66 | 0.032 | 0.001 | 0.013 | 0 | 'WDR59 |
| 209732_at | 7.1 | 7.08 | 5.86 | 0.05 | 0.002 | 0.05 | 0.002 | 'CLEC2B |
| 217714_x_at | 5.21 | 5.26 | 6.1 | 0.083 | 0.005 | 0.065 | 0.003 | 'STMN1 |
| 200700_s_at | 8.46 | 8.66 | 7.66 | 0.032 | 0.001 | 0.074 | 0.004 | 'KDELR2 |
| 218819_at | 6.63 | 6.03 | 5.27 | 0.182 | 0.025 | 0.038 | 0.001 | 'INTS6 |
| 38487_at | 6.93 | 7.08 | 6.43 | 0.075 | 0.004 | 0.195 | 0.032 | 'STAB1 |
| 206249_at | 5.15 | 5.13 | 5.84 | 0.086 | 0.006 | 0.067 | 0.003 | 'MAP3K13 |
| 212104_s_at | 6.12 | 6.26 | 6.88 | 0.074 | 0.004 | 0.078 | 0.005 | 'RBM9 |
| 215193_x_at | 9.95 | 10.72 | 11 | 0.6 | 0.316 | 0.043 | 0.001 | 'HLA-DRB1 |
| 203907_s_at | 6.26 | 6.52 | 7.2 | 0.08 | 0.005 | 0.058 | 0.002 | 'IQSEC1 |
| 205750_at | 6.29 | 6.19 | 6.77 | 0.054 | 0.002 | 0.094 | 0.007 | 'BPHL |
| 203306_s_at | 7.17 | 7.07 | 6.42 | 0.138 | 0.014 | 0.081 | 0.005 | 'SLC35A1 |
| 207244_x_at | 6.24 | 5.87 | 7.61 | 0.03 | 0.001 | 0.073 | 0.004 | 'CYP2A6 |
| 218533_s_at | 5.77 | 5.97 | 6.77 | 0.037 | 0.001 | 0.056 | 0.002 | 'UCKL1 |
| 200041_s_at | 9.5 | 9.52 | 10.02 | 0.061 | 0.003 | 0.062 | 0.003 | 'BAT1 |
| 203535_at | 9.01 | 8.5 | 7.8 | 0.205 | 0.032 | 0.053 | 0.002 | 'S100A9 |
| 218225_at | 6.72 | 5.98 | 7.2 | 0.014 | 0 | 0.289 | 0.069 | 'ECSIT |
| 201373_at | 6.79 | 7.04 | 7.69 | 0.177 | 0.024 | 0.062 | 0.003 | 'PLEC1 |
| 201255_x_at | 8.73 | 8.74 | 9.29 | 0.023 | 0 | 0.04 | 0.001 | 'BAT3 |
| 201659_s_at | 7.33 | 7.67 | 7 | 0.062 | 0.003 | 0.482 | 0.201 | 'ARL1 |
| 202737_s_at | 8.52 | 8.03 | 7.63 | 0.11 | 0.009 | 0.003 | 0 | 'LSM4 |
| 200780_x_at | 10.76 | 11.15 | 11.76 | 0.151 | 0.017 | 0.037 | 0.001 | 'GNAS |
| 209080_x_at | 7.78 | 7.85 | 7.33 | 0.039 | 0.001 | 0.067 | 0.003 | 'GLRX3 |
| 209639_s_at | 5.11 | 5.16 | 6.45 | 0.032 | 0.001 | 0.032 | 0.001 | 'RGS12 |
| 202957_at | 7.95 | 7.97 | 7.2 | 0.029 | 0.001 | 0.038 | 0.001 | 'HCLS1 |
| 221841_s_at | 8.42 | 8.4 | 7.12 | 0.09 | 0.006 | 0.087 | 0.006 | 'KLF4 |
| 213693_s_at | 10.79 | 10.55 | 9.71 | 0.078 | 0.005 | 0.043 | 0.001 | 'MUC1 |
| 204058_at | 5.11 | 5.69 | 3.18 | 0.033 | 0.001 | 0.07 | 0.004 | 'ME1 |
| 200641_s_at | 7.79 | 8.12 | 7 | 0.028 | 0.001 | 0.111 | 0.01 | 'YWHAZ |
| 200989_at | 8.24 | 7.98 | 7.04 | 0.15 | 0.017 | 0.079 | 0.005 | 'HIF1A |
| 204151_x_at | 11.68 | 11.8 | 10.77 | 0.03 | 0.001 | 0.071 | 0.004 | 'AKR1C1 |
| 203256_at | 6.5 | 6.6 | 7.45 | 0.104 | 0.008 | 0.107 | 0.009 | 'CDH3 |
| 213923_at | 6.86 | 6.97 | 6.11 | 0.041 | 0.001 | 0.07 | 0.004 | 'RAP2B |
| 218313_s_at | 7.59 | 7.81 | 6.96 | 0.062 | 0.003 | 0.132 | 0.014 | 'GALNT7 |
| 219182_at | 9.06 | 9.24 | 9.89 | 0.1 | 0.007 | 0.091 | 0.007 | 'FLJ22167 |
| 215498_s_at | 8.35 | 7.93 | 7.52 | 0.287 | 0.064 | 0.04 | 0.001 | 'MAP2K3 |
| 212761_at | 8.58 | 8.35 | 7.83 | 0.248 | 0.048 | 0.096 | 0.007 | 'TCF7L2 |
| 221016_s_at | 6.92 | 6.83 | 7.38 | 0.03 | 0.001 | 0.039 | 0.001 | 'TCF7L1 |
| 208720_s_at | 7.98 | 8.57 | 8.85 | 0.316 | 0.079 | 0.05 | 0.002 | 'RBM39 |
| 222108_at | 5.54 | 5.54 | 6.08 | 0.093 | 0.007 | 0.234 | 0.046 | 'AMIGO2 |
| 202042_at | 7.7 | 7.59 | 8.21 | 0.106 | 0.008 | 0.081 | 0.005 | 'HARS |
| 209556_at | 6.28 | 5.9 | 6.76 | 0.074 | 0.004 | 0.273 | 0.061 | 'NCDN |
| 211043_s_at | 8.76 | 8.17 | 8 | 0.678 | 0.412 | 0.048 | 0.002 | 'CLTB |
| 203021_at | 12.77 | 12.84 | 13.88 | 0.128 | 0.012 | 0.103 | 0.009 | 'SLPI |
| 200088_x_at | 11.83 | 11.83 | 12.52 | 0.062 | 0.003 | 0.059 | 0.003 | 'RPL12 |
| 201361_at | 7.9 | 7.81 | 8.1 | 0.097 | 0.007 | 0.26 | 0.056 | 'TMEM109 |
| 203417_at | 5.4 | 5.43 | 6.59 | 0.003 | 0 | 0.014 | 0 | 'MFAP2 |
| 213096_at | 6.02 | 5.72 | 6.77 | 0.038 | 0.001 | 0.081 | 0.005 | 'TMCC2 |
| 221004_s_at | 5.65 | 6.06 | 7 | 0.187 | 0.027 | 0.078 | 0.005 | 'ITM2C |
| 221187_s_at | 7.21 | 7.09 | 7.48 | 0.064 | 0.003 | 0.216 | 0.039 | 'FUZ |
| 218069_at | 8.35 | 7.99 | 7.76 | 0.492 | 0.207 | 0.053 | 0.002 | 'XTP3TPA |
| 209736_at | 6.04 | 6.37 | 6.97 | 0.08 | 0.005 | 0.035 | 0.001 | 'SOX13 |
| 212032_s_at | 7.77 | 7.57 | 8.28 | 0.056 | 0.002 | 0.36 | 0.11 | 'PTOV1 |
| 213361_at | 6.89 | 7.16 | 7.31 | 0.482 | 0.198 | 0.051 | 0.002 | 'TDRD7 |
| 212980_at | 5.14 | 5.38 | 6.31 | 0.066 | 0.003 | 0.029 | 0 | 'USP34 |
| 45572_s_at | 9.96 | 9.72 | 10.22 | 0.018 | 0 | 0.229 | 0.044 | 'GGA1 |
| 207783_x_at | 12.84 | 13.09 | 13.91 | 0.104 | 0.008 | 0.046 | 0.001 | 'HUWE1 |
| 217825_s_at | 6.9 | 6.89 | 6.09 | 0.062 | 0.003 | 0.06 | 0.003 | 'UBE2J1 |
| 204076_at | 7.58 | 7.58 | 6.9 | 0.072 | 0.004 | 0.065 | 0.003 | 'ENTPD4 |
| 200943_at | 8.65 | 9.27 | 9.81 | 0.192 | 0.028 | 0.063 | 0.003 | 'HMGN1 |
| 209270_at | 7.83 | 7.8 | 8.59 | 0.107 | 0.008 | 0.101 | 0.008 | 'LAMB3 |
| 218262_at | 6.41 | 6.4 | 6.87 | 0.022 | 0 | 0.027 | 0 | 'RMND5B |
| 218809_at | 8.6 | 8.22 | 7.99 | 0.496 | 0.212 | 0.104 | 0.009 | 'PANK2 |
| 207801_s_at | 7.69 | 7.65 | 7.39 | 0.167 | 0.021 | 0.09 | 0.006 | 'RNF10 |
| 203136_at | 9.1 | 8.83 | 8.52 | 0.387 | 0.123 | 0.1 | 0.008 | 'RABAC1 |
| 214150_x_at | 10.17 | 9.84 | 9.63 | 0.447 | 0.17 | 0.09 | 0.006 | 'ATP6V0E1 |
| 217776_at | 6.78 | 6.6 | 5.94 | 0.228 | 0.039 | 0.102 | 0.009 | 'RDH11 |
| 201573_s_at | 7.59 | 7.41 | 6.85 | 0.117 | 0.01 | 0.048 | 0.002 | 'ETF1 |
| 200735_x_at | 10.63 | 10.85 | 11.43 | 0.084 | 0.005 | 0.074 | 0.004 | 'NACA |
| 205499_at | 6.74 | 7.15 | 6.27 | 0.087 | 0.006 | 0.411 | 0.142 | 'SRPX2 |
| 209273_s_at | 7.17 | 6.81 | 6.13 | 0.171 | 0.022 | 0.053 | 0.002 | 'ISCA1 |
| 203687_at | 6.82 | 6.78 | 7.9 | 0.004 | 0 | 0.01 | 0 | 'CX3CL1 |
| 219797_at | 4.66 | 4.66 | 3.77 | 0.048 | 0.002 | 0.04 | 0.001 | 'MGAT4A |
| 203728_at | 6.94 | 6.52 | 6.23 | 0.536 | 0.251 | 0.093 | 0.007 | 'BAK1 |
| 200941_at | 9.3 | 8.94 | 8.64 | 0.284 | 0.063 | 0.053 | 0.002 | 'HSBP1 |
| 200888_s_at | 10.68 | 10.81 | 11.34 | 0.099 | 0.007 | 0.063 | 0.003 | 'RPL23 |
| 209455_at | 6.17 | 6.61 | 6.75 | 0.469 | 0.188 | 0.033 | 0.001 | 'FBXW11 |
| 218298_s_at | 6.37 | 6.57 | 7.32 | 0.002 | 0 | 0.014 | 0 | 'C14orf159 |
| 218907_s_at | 6.55 | 6.59 | 7.69 | 0.035 | 0.001 | 0.047 | 0.001 | 'LRRC61 |
| 212914_at | 6.79 | 6.59 | 7.61 | 0.009 | 0 | 0.061 | 0.003 | 'CBX7 |
| 222068_s_at | 9.26 | 9.55 | 10.77 | 0.001 | 0 | 0.009 | 0 | 'LRRC50 |
| 201226_at | 8.68 | 8.66 | 9.25 | 0.071 | 0.004 | 0.208 | 0.036 | 'NDUFB8 |
| 203638_s_at | 6.58 | 6.73 | 7.74 | 0.053 | 0.002 | 0.073 | 0.004 | 'FGFR2 |
| 207126_x_at | 8.03 | 8.54 | 7.94 | 0.025 | 0 | 0.91 | 0.8 | 'UGT1A4 |
| 213377_x_at | 12.17 | 12.19 | 12.84 | 0.065 | 0.003 | 0.065 | 0.003 | 'RPS12 |
| 213029_at | 7.67 | 7.63 | 8.21 | 0.027 | 0.001 | 0.047 | 0.002 | 'NFIB |
| 206262_at | 7.56 | 8.06 | 8.52 | 0.399 | 0.13 | 0.102 | 0.009 | 'ADH1A|ADH1B|ADH1C |
| 203227_s_at | 8.6 | 7.87 | 7.45 | 0.515 | 0.228 | 0.059 | 0.002 | 'TSPAN31 |
| 211979_at | 6.29 | 6.61 | 6.97 | 0.223 | 0.038 | 0.096 | 0.008 | 'GPR107 |
| 211075_s_at | 7.69 | 8.06 | 7.6 | 0.091 | 0.006 | 0.803 | 0.61 | 'CD47 |
| 213784_at | 7.46 | 7.54 | 7.88 | 0.151 | 0.017 | 0.099 | 0.008 | 'RABL4 |
| 205206_at | 7 | 6.97 | 8.29 | 0.008 | 0 | 0.021 | 0 | 'KAL1 |
| 201550_x_at | 12.06 | 12.3 | 12.69 | 0.228 | 0.04 | 0.078 | 0.005 | 'ACTG1 |
| 204589_at | 6.5 | 6.03 | 6.88 | 0.004 | 0 | 0.311 | 0.08 | 'NUAK1 |
| 205260_s_at | 5.37 | 5.94 | 6.56 | 0.08 | 0.005 | 0.057 | 0.002 | 'ACYP1 |
| 202212_at | 6.99 | 6.4 | 7.27 | 0.036 | 0.001 | 0.522 | 0.241 | 'PES1 |
| 220426_at | 6.65 | 6.62 | 7.21 | 0.074 | 0.004 | 0.108 | 0.01 | 'C20orf195 |
| 202772_at | 8.52 | 8.32 | 8.6 | 0.092 | 0.006 | 0.682 | 0.427 | 'HMGCL |
| 212688_at | 6.86 | 6.8 | 6.14 | 0.134 | 0.014 | 0.104 | 0.009 | 'PIK3CB |
| 212783_at | 6.64 | 6.81 | 7.21 | 0.184 | 0.026 | 0.059 | 0.003 | 'RBBP6 |
| 213101_s_at | 10.4 | 10.19 | 9.25 | 0.084 | 0.005 | 0.044 | 0.001 | 'ACTR3 |
| 218741_at | 7.06 | 6.45 | 7.45 | 0.002 | 0 | 0.287 | 0.067 | 'CENPM |
| 200053_at | 8.93 | 8.84 | 9.33 | 0.017 | 0 | 0.101 | 0.008 | 'SPAG7 |
| 206302_s_at | 6.75 | 6.73 | 5.87 | 0.076 | 0.004 | 0.071 | 0.004 | 'NUDT4|NUDT4P1 |
| 211711_s_at | 5.74 | 5.43 | 5.17 | 0.464 | 0.183 | 0.059 | 0.003 | 'PTEN |
| 203462_x_at | 9.04 | 8.75 | 9.1 | 0.059 | 0.003 | 0.889 | 0.76 | 'EIF3B |
| 213639_s_at | 6.03 | 6.07 | 6.59 | 0.077 | 0.004 | 0.035 | 0.001 | 'ZNF500 |
| 204846_at | 8.32 | 8.8 | 7.97 | 0.103 | 0.008 | 0.509 | 0.226 | 'CP |
| 215364_s_at | 5.75 | 6.08 | 6.37 | 0.238 | 0.043 | 0.04 | 0.001 | 'KIAA0467 |
| 205485_at | 5.13 | 5.36 | 6 | 0.17 | 0.022 | 0.103 | 0.009 | 'RYR1 |
| 219206_x_at | 8.36 | 8.09 | 7.92 | 0.647 | 0.372 | 0.093 | 0.007 | 'TMBIM4 |
| 53968_at | 7.83 | 7.49 | 7.29 | 0.449 | 0.171 | 0.078 | 0.005 | 'INTS5 |
| 36084_at | 6.97 | 6.96 | 7.5 | 0.093 | 0.007 | 0.108 | 0.01 | 'CUL7 |
| 219416_at | 4.98 | 5.1 | 5.93 | 0.073 | 0.004 | 0.045 | 0.001 | 'SCARA3 |
| 200099_s_at | 11.4 | 11.43 | 12.02 | 0.063 | 0.003 | 0.066 | 0.003 | 'LOC439992|RPS3A |
| 40093_at | 8.18 | 7.67 | 9.14 | 0.008 | 0 | 0.074 | 0.004 | 'BCAM |
| 221501_x_at | 8.83 | 9.55 | 10.44 | 0.01 | 0 | 0.021 | 0 | 'LOC339047 |
| 33322_i_at | 11.92 | 11.22 | 10.98 | 0.571 | 0.286 | 0.071 | 0.004 | 'SFN |
| 219848_s_at | 7 | 6.6 | 6.22 | 0.398 | 0.13 | 0.099 | 0.008 | 'ZNF432 |
| 213583_x_at | 12.18 | 12.35 | 13.25 | 0.146 | 0.016 | 0.09 | 0.007 | 'EEF1A1 |
| 215407_s_at | 5.69 | 5.47 | 6.22 | 0.09 | 0.006 | 0.189 | 0.03 | 'ASTN2 |
| 212376_s_at | 6.59 | 6.72 | 7.3 | 0.076 | 0.004 | 0.103 | 0.009 | 'EP400 |
| 201522_x_at | 9.31 | 9.34 | 10.04 | 0.023 | 0 | 0.026 | 0 | 'SNRPN|SNURF |
| 213980_s_at | 7.21 | 7.37 | 8 | 0.05 | 0.002 | 0.162 | 0.021 | 'CTBP1 |
| 204517_at | 8 | 7.75 | 7.08 | 0.129 | 0.013 | 0.048 | 0.002 | 'PPIC |
| 201221_s_at | 8.98 | 8.87 | 9.48 | 0.03 | 0.001 | 0.091 | 0.007 | 'SNRP70 |
| 218671_s_at | 8.99 | 8.91 | 9.48 | 0.05 | 0.002 | 0.121 | 0.012 | 'ATPIF1 |
| 212715_s_at | 5.24 | 4.9 | 6.35 | 0 | 0 | 0.014 | 0 | 'MICAL3 |
| 210413_x_at | 9.82 | 10.19 | 10.83 | 0.115 | 0.01 | 0.026 | 0 | 'SERPINB4 |
| 219670_at | 6.66 | 6.46 | 7 | 0.09 | 0.006 | 0.205 | 0.035 | 'C1orf165 |
| 206453_s_at | 9.22 | 9.14 | 9.51 | 0.022 | 0 | 0.191 | 0.03 | 'NDRG2 |
| 219246_s_at | 4.92 | 4.93 | 5.9 | 0.029 | 0.001 | 0.032 | 0.001 | 'OGFOD2 |
| 213979_s_at | 6.65 | 6.92 | 7.31 | 0.208 | 0.033 | 0.041 | 0.001 | 'CTBP1 |
| 212152_x_at | 8.53 | 8.57 | 9.12 | 0.053 | 0.002 | 0.046 | 0.001 | 'ARID1A |
| 219051_x_at | 7.24 | 6.91 | 7.83 | 0.066 | 0.003 | 0.338 | 0.096 | 'METRN |
| 200705_s_at | 10.25 | 10.17 | 10.54 | 0.018 | 0 | 0.186 | 0.029 | 'EEF1B2|EEF1B4 |
| 207843_x_at | 9.36 | 9.8 | 10.49 | 0.117 | 0.01 | 0.024 | 0 | 'CYB5A |
| 205221_at | 5.25 | 5.84 | 4.14 | 0.035 | 0.001 | 0.15 | 0.018 | 'HGD |
| 205278_at | 4.17 | 4.76 | 3 | 0.04 | 0.001 | 0.166 | 0.022 | 'GAD1 |
| 213135_at | 5.79 | 6.11 | 5.57 | 0.077 | 0.004 | 0.477 | 0.196 | 'TIAM1 |
| 205376_at | 6.21 | 5.78 | 5.25 | 0.241 | 0.044 | 0.04 | 0.001 | 'INPP4B |
| 209794_at | 8.11 | 7.79 | 8.59 | 0.003 | 0 | 0.083 | 0.006 | 'SRGAP3 |
| 202071_at | 10.43 | 10.25 | 10.6 | 0.08 | 0.005 | 0.524 | 0.242 | 'SDC4 |
| 207265_s_at | 7.16 | 7.27 | 6.57 | 0.053 | 0.002 | 0.088 | 0.006 | 'KDELR3 |
| 208817_at | 7.63 | 7.76 | 8.12 | 0.209 | 0.033 | 0.078 | 0.005 | 'COMT |
| 203781_at | 9.01 | 8.72 | 8.35 | 0.386 | 0.122 | 0.104 | 0.009 | 'MRPL33 |
| 202000_at | 7.15 | 7.29 | 7.55 | 0.26 | 0.052 | 0.096 | 0.007 | 'NDUFA6 |
| 200034_s_at | 10.08 | 10.35 | 10.81 | 0.103 | 0.008 | 0.08 | 0.005 | 'RPL6 |
| 203981_s_at | 5.8 | 5.48 | 6.25 | 0.025 | 0 | 0.165 | 0.022 | 'ABCD4 |
| 58916_at | 6.61 | 6.38 | 5.84 | 0.149 | 0.017 | 0.043 | 0.001 | 'KCTD14 |
| 208883_at | 5.27 | 5.4 | 6.08 | 0.022 | 0 | 0.009 | 0 | 'UBR5 |
| 219916_s_at | 7.53 | 7.43 | 6.52 | 0.05 | 0.002 | 0.035 | 0.001 | 'RNF39 |
| 202868_s_at | 7.94 | 7.72 | 7.68 | 0.848 | 0.68 | 0.096 | 0.007 | 'POP4 |
| 46665_at | 7.54 | 7.43 | 8.42 | 0.006 | 0 | 0.022 | 0 | 'SEMA4C |
| 201001_s_at | 5.83 | 6.28 | 5.56 | 0.097 | 0.007 | 0.548 | 0.268 | 'TMEM189-UBE2V1|UBE2V1 |
| 214946_x_at | 7.51 | 7.83 | 8.3 | 0.066 | 0.003 | 0.007 | 0 | 'FAM21C |
| 218976_at | 6.72 | 6.75 | 5.63 | 0.017 | 0 | 0.03 | 0 | 'DNAJC12 |
| 200081_s_at | 11.07 | 11.12 | 11.85 | 0.132 | 0.013 | 0.095 | 0.007 | 'RPS6 |
| 220625_s_at | 5.83 | 5.62 | 6.82 | 0.003 | 0 | 0.009 | 0 | 'ELF5 |
| 212522_at | 5.33 | 5.51 | 6.03 | 0.212 | 0.035 | 0.079 | 0.005 | 'PDE8A |
| 208811_s_at | 5.29 | 5.97 | 6.4 | 0.413 | 0.142 | 0.041 | 0.001 | 'DNAJB6 |
| 203522_at | 5.76 | 5.28 | 6.38 | 0.074 | 0.004 | 0.347 | 0.102 | 'CCS |
| 221768_at | 5.6 | 6.06 | 6.41 | 0.444 | 0.166 | 0.103 | 0.009 | 'SFPQ |
| 217756_x_at | 10.49 | 10.61 | 10.23 | 0.068 | 0.004 | 0.34 | 0.097 | 'SERF2 |
| 211999_at | 9.79 | 10.03 | 10.58 | 0.072 | 0.004 | 0.048 | 0.002 | 'H3F3B |
| 210397_at | 7.67 | 7.27 | 6.01 | 0.033 | 0.001 | 0.017 | 0 | 'DEFB1 |
| 214088_s_at | 7.95 | 8.08 | 7.36 | 0.003 | 0 | 0.053 | 0.002 | 'FUT3 |
| 214173_x_at | 7.23 | 7.23 | 7.6 | 0.248 | 0.047 | 0.095 | 0.007 | 'C19orf2 |
| 218302_at | 9.2 | 9.19 | 8.48 | 0.113 | 0.009 | 0.09 | 0.007 | 'PSENEN |
| 221475_s_at | 10.08 | 10.35 | 10.95 | 0.189 | 0.027 | 0.043 | 0.001 | 'RPL15 |
| 220030_at | 5.43 | 5 | 3.81 | 0.114 | 0.009 | 0.052 | 0.002 | 'STYK1 |
| 218249_at | 6.97 | 7.15 | 6.71 | 0.083 | 0.005 | 0.327 | 0.089 | 'ZDHHC6 |
| 211710_x_at | 11.45 | 11.43 | 12.2 | 0.069 | 0.004 | 0.073 | 0.004 | 'RPL4 |
| 219664_s_at | 6.58 | 6.64 | 5.74 | 0.058 | 0.003 | 0.081 | 0.005 | 'DECR2 |
| 204734_at | 8.8 | 8.57 | 10.17 | 0.015 | 0 | 0.035 | 0.001 | 'KRT15 |
| 218412_s_at | 7.37 | 7.45 | 7.8 | 0.05 | 0.002 | 0.066 | 0.003 | 'GTF2IRD1 |
| 219940_s_at | 6.44 | 6.71 | 7.19 | 0.074 | 0.004 | 0.04 | 0.001 | 'PCID2 |
| 219164_s_at | 4.46 | 4.62 | 5.3 | 0.067 | 0.003 | 0.051 | 0.002 | 'ATG2B |
| 203239_s_at | 5.66 | 5.75 | 6.33 | 0.066 | 0.003 | 0.059 | 0.003 | 'CNOT3 |
| 205259_at | 5.86 | 5.59 | 6.46 | 0.025 | 0 | 0.06 | 0.003 | 'NR3C2 |
| 38918_at | 5.89 | 6.03 | 6.82 | 0.052 | 0.002 | 0.035 | 0.001 | 'SOX13 |
| 219489_s_at | 7.98 | 7.34 | 8.08 | 0.028 | 0.001 | 0.779 | 0.572 | 'NXN |
| 204806_x_at | 9.71 | 10.36 | 10.71 | 0.411 | 0.14 | 0.03 | 0 | 'HLA-F |
| 214073_at | 5.94 | 5.32 | 4.5 | 0.161 | 0.02 | 0.025 | 0 | 'CTTN |
| 210791_s_at | 5.72 | 5.99 | 6.59 | 0.295 | 0.068 | 0.082 | 0.005 | 'RICS |
| 204204_at | 5.95 | 6.11 | 5.37 | 0.032 | 0.001 | 0.075 | 0.004 | 'SLC31A2 |
| 204341_at | 7.51 | 7.73 | 6.83 | 0.021 | 0 | 0.066 | 0.003 | 'TRIM16|TRIM16L |
| 220917_s_at | 7.65 | 7.77 | 8.32 | 0.092 | 0.006 | 0.111 | 0.01 | 'WDR19 |
| 218023_s_at | 7.45 | 7.32 | 7.63 | 0.06 | 0.003 | 0.419 | 0.148 | 'FAM53C |
| 217734_s_at | 6.88 | 7.13 | 7.79 | 0.177 | 0.024 | 0.073 | 0.004 | 'WDR6 |
| 212655_at | 5.48 | 5.84 | 6.63 | 0.04 | 0.001 | 0.039 | 0.001 | 'ZCCHC14 |
| 220998_s_at | 7.06 | 6.94 | 7.96 | 0.006 | 0 | 0.018 | 0 | 'UNC93B1 |
| 218524_at | 5.68 | 5.57 | 6.35 | 0.099 | 0.007 | 0.17 | 0.024 | 'E4F1 |
| 217991_x_at | 6.69 | 6.73 | 7.28 | 0.025 | 0 | 0.021 | 0 | 'SSBP3 |
| 204060_s_at | 6.85 | 7.1 | 7.57 | 0.161 | 0.019 | 0.1 | 0.008 | 'PRKX|PRKY |
| 210166_at | 7.02 | 6.94 | 7.95 | 0.003 | 0 | 0.017 | 0 | 'TLR5 |
| 217910_x_at | 7.16 | 7.26 | 7.68 | 0.089 | 0.006 | 0.049 | 0.002 | 'MLX |
| 210224_at | 6.65 | 6.52 | 6.25 | 0.131 | 0.013 | 0.081 | 0.005 | 'MR1 |
| 208812_x_at | 12.33 | 12.5 | 13.19 | 0.095 | 0.007 | 0.041 | 0.001 | 'HLA-C |
| 210582_s_at | 8.32 | 8.49 | 8.87 | 0.055 | 0.002 | 0.014 | 0 | 'LIMK2 |
| 202090_s_at | 10.28 | 9.92 | 9.59 | 0.333 | 0.088 | 0.059 | 0.003 | 'UQCR|TMEM54 |
| 207191_s_at | 5.55 | 5.36 | 6.31 | 0.076 | 0.004 | 0.168 | 0.023 | 'ISLR |
| 218030_at | 7.09 | 6.89 | 7.72 | 0.074 | 0.004 | 0.157 | 0.02 | 'GIT1 |
| 213169_at | 7.01 | 6.62 | 7.6 | 0.023 | 0 | 0.024 | 0 | 'SEMA5A |
| 203683_s_at | 5.16 | 4.97 | 6.34 | 0.021 | 0 | 0.043 | 0.001 | 'VEGFB |
| 212859_x_at | 9.99 | 10.09 | 10.98 | 0.047 | 0.002 | 0.043 | 0.001 | 'MT1E |
| 221708_s_at | 6.91 | 6.53 | 7.09 | 0.042 | 0.001 | 0.32 | 0.086 | 'UNC45A |
| 204017_at | 7.34 | 7.29 | 6.31 | 0.052 | 0.002 | 0.047 | 0.002 | 'KDELR3 |
| 204508_s_at | 4.21 | 4.34 | 2.18 | 0.029 | 0.001 | 0.042 | 0.001 | 'CA12 |
| 213347_x_at | 11.94 | 11.74 | 12.46 | 0.017 | 0 | 0.067 | 0.003 | 'RPS4X |
| 220800_s_at | 3.53 | 3.45 | 1.97 | 0.103 | 0.008 | 0.094 | 0.007 | 'TMOD3 |
| 200687_s_at | 6.88 | 6.95 | 7.41 | 0.005 | 0 | 0.081 | 0.005 | 'SF3B3 |
| 204718_at | 5.51 | 5.6 | 6.53 | 0.081 | 0.005 | 0.06 | 0.003 | 'EPHB6 |
| 200623_s_at | 7.49 | 7.77 | 8.48 | 0.095 | 0.007 | 0.046 | 0.001 | 'CALM3 |
| 214164_x_at | 7.87 | 7.38 | 5.89 | 0.041 | 0.001 | 0.017 | 0 | 'CA12 |
| 212969_x_at | 6.82 | 6.66 | 7.52 | 0.011 | 0 | 0.032 | 0.001 | 'EML3 |
| 202272_s_at | 6.2 | 6.4 | 6.04 | 0.036 | 0.001 | 0.481 | 0.199 | 'FBXO28 |
| 218208_at | 8.44 | 7.78 | 8.35 | 0.069 | 0.004 | 0.829 | 0.656 | 'PQLC1 |
| 204216_s_at | 5.9 | 6.21 | 6.74 | 0.282 | 0.061 | 0.081 | 0.005 | 'ZC3H14 |
| 200747_s_at | 7.43 | 7.53 | 8.52 | 0.008 | 0 | 0.014 | 0 | 'NUMA1 |
| 216347_s_at | 6.32 | 6.49 | 6.84 | 0.245 | 0.046 | 0.08 | 0.005 | 'PPP1R13B |
| 200931_s_at | 7.82 | 8.03 | 7.44 | 0.103 | 0.008 | 0.306 | 0.077 | 'VCL |
| 221216_s_at | 6.7 | 6.72 | 7.43 | 0.094 | 0.007 | 0.079 | 0.005 | 'SCMH1 |
| 205844_at | 3.92 | 5.04 | 5.19 | 0.754 | 0.527 | 0.059 | 0.003 | 'VNN1 |
| 202059_s_at | 7.3 | 7.18 | 6.92 | 0.279 | 0.06 | 0.078 | 0.005 | 'KPNA1 |
| 218379_at | 6.68 | 6.55 | 6.15 | 0.374 | 0.115 | 0.097 | 0.008 | 'RBM7 |
| 206042_x_at | 6.86 | 7.08 | 7.62 | 0.132 | 0.013 | 0.067 | 0.003 | 'SNRPN|SNURF |
| 219597_s_at | 8.69 | 8.65 | 9.1 | 0.063 | 0.003 | 0.124 | 0.013 | 'DUOX1 |
| 213441_x_at | 8.62 | 9.19 | 8.22 | 0.026 | 0.001 | 0.338 | 0.096 | 'SPDEF |
| 202856_s_at | 7.69 | 7.46 | 7.14 | 0.296 | 0.068 | 0.088 | 0.006 | 'SLC16A3 |
| 200990_at | 8.21 | 8.28 | 8.61 | 0.052 | 0.002 | 0.379 | 0.121 | 'TRIM28 |
| 200763_s_at | 12.38 | 12.28 | 13.06 | 0.084 | 0.005 | 0.123 | 0.013 | 'RPLP1 |
| 209379_s_at | 6.52 | 6.06 | 5.66 | 0.407 | 0.137 | 0.075 | 0.004 | 'KIAA1128 |
| 205073_at | 6.63 | 7.06 | 7.73 | 0.159 | 0.019 | 0.083 | 0.005 | 'CYP2J2 |
| 216996_s_at | 6.91 | 6.88 | 6.32 | 0.103 | 0.008 | 0.087 | 0.006 | 'FASTKD2 |
| 50376_at | 8.03 | 7.82 | 8.3 | 0.071 | 0.004 | 0.396 | 0.132 | 'ZNF444 |
| 200926_at | 12 | 12.12 | 12.89 | 0.078 | 0.005 | 0.055 | 0.002 | 'RPS23 |
| 222125_s_at | 9.53 | 9.55 | 9.9 | 0.04 | 0.001 | 0.14 | 0.016 | 'PH-4 |
| 37424_at | 7.02 | 6.84 | 7.59 | 0.101 | 0.008 | 0.214 | 0.038 | 'CCHCR1 |
| 212904_at | 7.92 | 7.92 | 8.32 | 0.062 | 0.003 | 0.204 | 0.034 | 'LRRC47 |
| 205530_at | 4.97 | 5.16 | 5.55 | 0.224 | 0.039 | 0.104 | 0.009 | 'ETFDH |
| 203733_at | 6.64 | 6.44 | 7.25 | 0.018 | 0 | 0.136 | 0.015 | 'DEXI |
| 204131_s_at | 8.21 | 8.23 | 8.83 | 0.021 | 0 | 0.026 | 0 | 'FOXO3 |
| 211797_s_at | 3.47 | 4.26 | 4.84 | 0.441 | 0.163 | 0.091 | 0.007 | 'NFYC |
| 221738_at | 6.1 | 6.06 | 5.54 | 0.058 | 0.003 | 0.096 | 0.008 | 'KIAA1219 |
| 204892_x_at | 12.8 | 13.19 | 14.36 | 0.165 | 0.02 | 0.074 | 0.004 | 'EEF1A1 |
| 200963_x_at | 12.01 | 12.04 | 12.58 | 0.066 | 0.003 | 0.045 | 0.001 | 'RPL31 |
| 217871_s_at | 9.83 | 9.55 | 9.25 | 0.359 | 0.104 | 0.076 | 0.004 | 'MIF |
| 218244_at | 5.25 | 5.29 | 5.92 | 0.09 | 0.006 | 0.194 | 0.031 | 'NOL8 |
| 213951_s_at | 4.15 | 4.24 | 5.06 | 0.109 | 0.009 | 0.074 | 0.004 | 'PSMC3IP |
| 200022_at | 11.23 | 11.21 | 11.86 | 0.014 | 0 | 0.032 | 0.001 | 'RPL18 |
| 202216_x_at | 6.29 | 6.6 | 7 | 0.314 | 0.078 | 0.083 | 0.005 | 'NFYC |
| 218380_at | 8.18 | 8.22 | 8.59 | 0.078 | 0.005 | 0.216 | 0.039 | 'NLRP1 |
| 213601_at | 7.28 | 7.04 | 7.8 | 0.004 | 0 | 0.04 | 0.001 | 'SLIT1 |
| 205482_x_at | 5.83 | 5.95 | 6.6 | 0.159 | 0.019 | 0.068 | 0.003 | 'SNX15 |
| 212871_at | 4.81 | 5.34 | 5.76 | 0.147 | 0.016 | 0.046 | 0.001 | 'MAPKAPK5 |
| 212039_x_at | 12.81 | 12.6 | 13.17 | 0.09 | 0.006 | 0.294 | 0.071 | 'RPL3 |
| 40225_at | 9.25 | 9.04 | 8.69 | 0.243 | 0.045 | 0.089 | 0.006 | 'GAK |
| 204179_at | 8.87 | 8.55 | 9.26 | 0.061 | 0.003 | 0.326 | 0.088 | 'MB |
| 213084_x_at | 12.97 | 12.98 | 13.55 | 0.112 | 0.009 | 0.1 | 0.008 | 'RPL23A |
| 211911_x_at | 11.21 | 11.7 | 12.31 | 0.173 | 0.022 | 0.014 | 0 | 'HLA-B |
| 218025_s_at | 6.36 | 5.84 | 7 | 0.002 | 0 | 0.047 | 0.001 | 'PECI |
| 220403_s_at | 4.34 | 3.88 | 4.93 | 0.081 | 0.005 | 0.343 | 0.099 | 'P53AIP1 |
| 200956_s_at | 6.16 | 6.28 | 7.05 | 0.174 | 0.023 | 0.084 | 0.006 | 'SSRP1 |
| 200935_at | 4.89 | 5.68 | 6.08 | 0.491 | 0.206 | 0.07 | 0.004 | 'CALR |
| 221731_x_at | 6.93 | 6.71 | 6.03 | 0.102 | 0.008 | 0.038 | 0.001 | 'VCAN |
| 219094_at | 3.42 | 3.45 | 4.69 | 0.044 | 0.002 | 0.076 | 0.004 | 'ARMC8 |
| 217165_x_at | 8.1 | 8.03 | 8.84 | 0.029 | 0.001 | 0.166 | 0.022 | 'MT1F |
| 40837_at | 7.24 | 7.28 | 8 | 0.017 | 0 | 0.017 | 0 | 'TLE2 |
| 201837_s_at | 6.26 | 6.6 | 6.79 | 0.485 | 0.202 | 0.082 | 0.005 | 'SUPT7L |
| 202840_at | 6.26 | 6.73 | 7.31 | 0.316 | 0.079 | 0.085 | 0.006 | 'TAF15 |
| 202299_s_at | 8.6 | 8.78 | 8.99 | 0.382 | 0.119 | 0.097 | 0.008 | 'HBXIP |
| 216071_x_at | 6.29 | 6.4 | 7.05 | 0.036 | 0.001 | 0.123 | 0.012 | 'MED12 |
| 212693_at | 6.45 | 6.56 | 6.94 | 0.106 | 0.008 | 0.099 | 0.008 | 'MDN1 |
| 217994_x_at | 8.07 | 7.93 | 8.4 | 0.099 | 0.007 | 0.256 | 0.054 | 'CPSF3L |
| 201970_s_at | 5.67 | 5.95 | 7 | 0.036 | 0.001 | 0.051 | 0.002 | 'NASP |
| 216032_s_at | 9.72 | 9.75 | 10.33 | 0.056 | 0.002 | 0.085 | 0.006 | 'ERGIC3 |
| 219401_at | 5.75 | 5.36 | 6.24 | 0.086 | 0.006 | 0.308 | 0.078 | 'XYLT2 |
| 46323_at | 8.08 | 7.75 | 7.34 | 0.24 | 0.044 | 0.047 | 0.001 | 'CANT1 |
| 217769_s_at | 9.94 | 9.8 | 9.34 | 0.178 | 0.024 | 0.083 | 0.005 | 'POMP |
| 204524_at | 5.34 | 5.85 | 6.63 | 0.161 | 0.019 | 0.041 | 0.001 | 'PDPK1 |
| 216231_s_at | 11.79 | 12.23 | 12.7 | 0.388 | 0.124 | 0.027 | 0 | 'B2M |
| 218885_s_at | 7.28 | 7.76 | 6.85 | 0.055 | 0.002 | 0.545 | 0.264 | 'GALNT12 |
| 203710_at | 5.15 | 5.73 | 5.99 | 0.531 | 0.244 | 0.085 | 0.006 | 'ITPR1 |
| 213505_s_at | 5.86 | 5.86 | 7.23 | 0.002 | 0 | 0.009 | 0 | 'SFRS14 |
| 200937_s_at | 9.78 | 9.94 | 10.79 | 0.025 | 0 | 0.037 | 0.001 | 'RPL5 |
| 218839_at | 7.58 | 7.4 | 8.14 | 0.023 | 0 | 0.22 | 0.041 | 'HEY1 |
| 204731_at | 5.23 | 5.36 | 5.94 | 0.09 | 0.006 | 0.043 | 0.001 | 'TGFBR3 |
| 218751_s_at | 5.45 | 5.52 | 6.27 | 0.119 | 0.01 | 0.085 | 0.006 | 'FBXW7 |
| 200730_s_at | 5.66 | 5.91 | 4.71 | 0.08 | 0.005 | 0.166 | 0.022 | 'PTP4A1 |
| 202725_at | 8.01 | 7.9 | 8.35 | 0.061 | 0.003 | 0.194 | 0.032 | 'POLR2A |
| 208619_at | 9.33 | 9.04 | 8.85 | 0.363 | 0.107 | 0.035 | 0.001 | 'DDB1 |
| 219872_at | 1.54 | 2.01 | 3.64 | 0.19 | 0.027 | 0.094 | 0.007 | 'C4orf18 |
| 209117_at | 8.06 | 7.98 | 8.55 | 0.048 | 0.002 | 0.112 | 0.01 | 'WBP2 |
| 213932_x_at | 12.3 | 12.47 | 12.93 | 0.124 | 0.011 | 0.06 | 0.003 | 'HLA-A |
| 208921_s_at | 10.14 | 10.08 | 10.58 | 0.017 | 0 | 0.122 | 0.012 | 'SRI |
| 220344_at | 7.85 | 7.72 | 8.36 | 0.034 | 0.001 | 0.176 | 0.025 | 'C11orf16 |
| 209784_s_at | 7.13 | 7.02 | 8.19 | 0 | 0 | 0.002 | 0 | 'JAG2 |
| 212399_s_at | 8.16 | 7.89 | 8.32 | 0.051 | 0.002 | 0.532 | 0.25 | 'VGLL4 |
| 213179_at | 7.44 | 7.49 | 7.76 | 0.125 | 0.012 | 0.065 | 0.003 | 'RQCD1 |
| 203826_s_at | 7.58 | 7.38 | 7.79 | 0.087 | 0.006 | 0.462 | 0.184 | 'PITPNM1 |
| 217992_s_at | 7.63 | 7.94 | 7.39 | 0.018 | 0 | 0.389 | 0.128 | 'EFHD2 |
| 201459_at | 9.63 | 9.54 | 10.09 | 0.003 | 0 | 0.09 | 0.006 | 'RUVBL2 |
| 212017_at | 7.38 | 7.66 | 8.04 | 0.177 | 0.024 | 0.094 | 0.007 | 'FAM168B |
| 210345_s_at | 8.11 | 8.15 | 8.67 | 0.09 | 0.006 | 0.09 | 0.006 | 'DNAH9 |
| 204064_at | 6.62 | 6.68 | 7.16 | 0.064 | 0.003 | 0.06 | 0.003 | 'THOC1 |
| 210527_x_at | 6.68 | 6.93 | 7.49 | 0.145 | 0.016 | 0.047 | 0.002 | 'TUBA3C |
| 201049_s_at | 12.53 | 12.52 | 13.26 | 0.088 | 0.006 | 0.083 | 0.006 | 'RPS18|RP5-1033B10.18 |
| 209160_at | 9.38 | 9.5 | 8.44 | 0.02 | 0 | 0.072 | 0.004 | 'AKR1C3 |
| 211729_x_at | 8.74 | 8.49 | 8.3 | 0.394 | 0.127 | 0.061 | 0.003 | 'BLVRA |
| 221746_at | 6.5 | 6.42 | 7.16 | 0.028 | 0.001 | 0.059 | 0.003 | 'UBL4A |
| 212523_s_at | 4.18 | 4.81 | 5.44 | 0.2 | 0.03 | 0.047 | 0.002 | 'KIAA0146 |
| 209447_at | 6.24 | 6.53 | 7.8 | 0.009 | 0 | 0.024 | 0 | 'SYNE1 |
| 212179_at | 6.35 | 6.41 | 7.05 | 0.017 | 0 | 0.026 | 0 | 'SFRS18 |
| 200878_at | 9.87 | 9.97 | 11.16 | 0.005 | 0 | 0.009 | 0 | 'EPAS1 |
| 209061_at | 5.84 | 6.39 | 7.03 | 0.165 | 0.02 | 0.029 | 0 | 'NCOA3 |
| 208792_s_at | 9.2 | 9.41 | 10.06 | 0.199 | 0.03 | 0.096 | 0.007 | 'CLU |
| 216323_x_at | 6.55 | 6.82 | 7.39 | 0.238 | 0.043 | 0.081 | 0.005 | 'TUBA3D |
| 213477_x_at | 12.67 | 12.87 | 13.66 | 0.173 | 0.023 | 0.091 | 0.007 | 'EEF1A1 |
| 214271_x_at | 11.03 | 11.06 | 11.68 | 0.092 | 0.006 | 0.073 | 0.004 | 'RPL12 |
| 208153_s_at | 6.05 | 5.92 | 6.96 | 0.012 | 0 | 0.047 | 0.002 | 'FAT2 |
| 219749_at | 6.78 | 6.89 | 6.34 | 0 | 0 | 0.024 | 0 | 'SH2D4A |
| 213488_at | 6.2 | 6.16 | 6.93 | 0.006 | 0 | 0.011 | 0 | 'SNED1 |
| 201530_x_at | 10.94 | 10.81 | 10.44 | 0.117 | 0.01 | 0.034 | 0.001 | 'EIF4A1 |
| 209074_s_at | 4.42 | 4.25 | 7.19 | 0.001 | 0 | 0.006 | 0 | 'FAM107A |
| 217904_s_at | 5.09 | 5.19 | 5.95 | 0.036 | 0.001 | 0.018 | 0 | 'BACE1 |
| 204262_s_at | 5.78 | 5.68 | 6.15 | 0.042 | 0.001 | 0.157 | 0.02 | 'PSEN2 |
| 213590_at | 6.44 | 6.66 | 6.97 | 0.089 | 0.006 | 0.019 | 0 | 'SLC16A5 |
| 215071_s_at | 7.52 | 7.57 | 6.96 | 0.074 | 0.004 | 0.145 | 0.017 | 'HIST1H2AC |
| 201455_s_at | 6.58 | 7.1 | 7.26 | 0.652 | 0.377 | 0.079 | 0.005 | 'NPEPPS |
| 201322_at | 10.06 | 10.27 | 10.61 | 0.174 | 0.023 | 0.066 | 0.003 | 'ATP5B |
| 207064_s_at | 5.43 | 5.18 | 5.71 | 0.103 | 0.008 | 0.576 | 0.298 | 'AOC2 |
| 204620_s_at | 6.62 | 6.6 | 6.06 | 0.104 | 0.008 | 0.166 | 0.022 | 'VCAN |
| 209702_at | 7.84 | 7.93 | 8.8 | 0.129 | 0.012 | 0.1 | 0.008 | 'FTO |
| 209125_at | 6.51 | 6.59 | 4.38 | 0.095 | 0.007 | 0.1 | 0.008 | 'KRT6C|KRT6A |
| 202624_s_at | 6.81 | 7.11 | 7.85 | 0.041 | 0.001 | 0.013 | 0 | 'CABIN1 |
| 204802_at | 9.11 | 8.38 | 9.39 | 0.092 | 0.006 | 0.683 | 0.428 | 'RRAD |
| 203871_at | 6.03 | 6.3 | 6.67 | 0.352 | 0.099 | 0.092 | 0.007 | 'SENP3 |
| 209921_at | 5.37 | 6.28 | 4.78 | 0.022 | 0 | 0.411 | 0.142 | 'SLC7A11 |
| 200089_s_at | 10.82 | 10.53 | 11.02 | 0.066 | 0.003 | 0.475 | 0.195 | 'RPL4 |
| 201066_at | 7.67 | 7.7 | 8.19 | 0.099 | 0.007 | 0.068 | 0.003 | 'CYC1 |
| 214016_s_at | 6.69 | 6.75 | 7.42 | 0.015 | 0 | 0.042 | 0.001 | 'SFPQ |
| 210006_at | 7.14 | 7.07 | 7.34 | 0.096 | 0.007 | 0.231 | 0.044 | 'ABHD14A |
| 204379_s_at | 8.54 | 8.25 | 9.34 | 0.006 | 0 | 0.04 | 0.001 | 'FGFR3 |
| 211889_x_at | 5.18 | 5.38 | 4.37 | 0.079 | 0.005 | 0.151 | 0.018 | 'CEACAM1 |
| 203058_s_at | 5.38 | 5.02 | 5.68 | 0.085 | 0.006 | 0.399 | 0.134 | 'PAPSS2 |
| 221780_s_at | 6.16 | 5.84 | 6.82 | 0.023 | 0 | 0.079 | 0.005 | 'DDX27 |
| 33850_at | 5.7 | 5.51 | 6.35 | 0.051 | 0.002 | 0.116 | 0.011 | 'MAP4 |
| 201272_at | 8.49 | 8.49 | 7.99 | 0.103 | 0.008 | 0.109 | 0.01 | 'AKR1B1 |
| 212153_at | 6.47 | 7.27 | 8.06 | 0.023 | 0 | 0.009 | 0 | 'POGZ |
| 208928_at | 8.26 | 8.19 | 8.71 | 0.068 | 0.003 | 0.157 | 0.02 | 'POR |
| 220269_at | 8.04 | 8.03 | 8.69 | 0.079 | 0.005 | 0.094 | 0.007 | 'ZBBX |
| 215735_s_at | 5.8 | 6.01 | 7.01 | 0.022 | 0 | 0.045 | 0.001 | 'TSC2 |
| 200959_at | 7.67 | 7.69 | 8.2 | 0.081 | 0.005 | 0.068 | 0.003 | 'FUS |
| 204351_at | 12.3 | 12.02 | 11.29 | 0.05 | 0.002 | 0.011 | 0 | 'S100P |
| 40016_g_at | 8.3 | 8.19 | 8.73 | 0.056 | 0.002 | 0.152 | 0.019 | 'MAST4 |
| 204480_s_at | 8.84 | 8.58 | 8.29 | 0.349 | 0.098 | 0.076 | 0.004 | 'C9orf16 |
| 47530_at | 6.85 | 6.63 | 6.23 | 0.161 | 0.019 | 0.042 | 0.001 | 'C9orf156 |
| 200715_x_at | 11.34 | 11.12 | 11.59 | 0.018 | 0 | 0.273 | 0.061 | 'RPL13A |
| 221833_at | 4.58 | 5.2 | 5.8 | 0.399 | 0.13 | 0.104 | 0.009 | 'SIAH1 |
| 209950_s_at | 8.23 | 7.96 | 8.59 | 0.051 | 0.002 | 0.346 | 0.101 | 'VILL |
| 203442_x_at | 7.11 | 6.84 | 7.46 | 0.072 | 0.004 | 0.28 | 0.065 | 'EML3 |
| 206561_s_at | 7.25 | 7.73 | 4.52 | 0.011 | 0 | 0.031 | 0 | 'AKR1B10 |
| 64440_at | 7.64 | 7.6 | 7.2 | 0.078 | 0.005 | 0.072 | 0.004 | 'IL17RC |
| 218753_at | 8.05 | 8.19 | 8.39 | 0.401 | 0.132 | 0.103 | 0.009 | 'XKR8 |
| 200909_s_at | 11.43 | 11.36 | 12.16 | 0.103 | 0.008 | 0.125 | 0.013 | 'RPLP2 |
| 200783_s_at | 5.62 | 5.39 | 6.22 | 0.08 | 0.005 | 0.215 | 0.039 | 'STMN1 |
| 204404_at | 7.44 | 7.53 | 7.07 | 0.07 | 0.004 | 0.182 | 0.027 | 'SLC12A2 |
| 200064_at | 10.82 | 11.03 | 11.82 | 0.049 | 0.002 | 0.038 | 0.001 | 'HSP90AB1 |
| 203492_x_at | 5.75 | 6.04 | 6.37 | 0.301 | 0.071 | 0.061 | 0.003 | 'CEP57 |
| 211928_at | 8.17 | 8.17 | 8.76 | 0.041 | 0.001 | 0.077 | 0.004 | 'DYNC1H1 |
| 205109_s_at | 6.52 | 6.33 | 7.14 | 0.031 | 0.001 | 0.081 | 0.005 | 'ARHGEF4 |
| 209389_x_at | 9.24 | 9.38 | 9.74 | 0.163 | 0.02 | 0.088 | 0.006 | 'DBI |
| 204041_at | 5.88 | 5.73 | 7.44 | 0.021 | 0 | 0.047 | 0.001 | 'MAOB |
| 207159_x_at | 5.49 | 5.61 | 6.43 | 0.117 | 0.01 | 0.078 | 0.005 | 'CRTC1 |
| 216336_x_at | 8.76 | 8.69 | 9.37 | 0.04 | 0.001 | 0.08 | 0.005 | 'MT1M |
| 211927_x_at | 12.21 | 12.13 | 12.68 | 0.102 | 0.008 | 0.145 | 0.017 | 'EEF1G|LOC729998 |
| 218763_at | 7.52 | 7.9 | 7.58 | 0.052 | 0.002 | 0.865 | 0.719 | 'STX18 |
| 201425_at | 9.4 | 9.4 | 9.89 | 0.05 | 0.002 | 0.102 | 0.008 | 'ALDH2 |
| 217807_s_at | 11.71 | 11.27 | 12.12 | 0.077 | 0.004 | 0.438 | 0.164 | 'GLTSCR2 |
| 203547_at | 6.72 | 6.89 | 7.38 | 0.172 | 0.022 | 0.103 | 0.009 | 'CD4 |
| 209875_s_at | 5.54 | 5.83 | 4.75 | 0.027 | 0.001 | 0.215 | 0.039 | 'SPP1 |
| 201204_s_at | 9.38 | 8.83 | 8.68 | 0.665 | 0.395 | 0.037 | 0.001 | 'RRBP1 |
| 209107_x_at | 7.72 | 8.03 | 8.31 | 0.186 | 0.026 | 0.057 | 0.002 | 'NCOA1 |
| 202251_at | 5.42 | 5.6 | 6.55 | 0.017 | 0 | 0.025 | 0 | 'PRPF3 |
| 203107_x_at | 12.19 | 12.23 | 12.76 | 0.097 | 0.007 | 0.051 | 0.002 | 'RPS2 |
| 202436_s_at | 6.86 | 8.75 | 5.44 | 0 | 0 | 0.059 | 0.003 | 'CYP1B1 |
| 204538_x_at | 8.71 | 9.32 | 10.26 | 0.022 | 0 | 0.014 | 0 | 'NPIP |
| 210065_s_at | 9.04 | 9.09 | 7.65 | 0.075 | 0.004 | 0.081 | 0.005 | 'UPK1B |
| 211026_s_at | 9.11 | 9 | 9.53 | 0.05 | 0.002 | 0.114 | 0.01 | 'MGLL |
| 210501_x_at | 9.97 | 9.82 | 10.22 | 0.036 | 0.001 | 0.26 | 0.055 | 'EIF3K |
| 214203_s_at | 7.54 | 7.09 | 7.94 | 0.041 | 0.001 | 0.438 | 0.164 | 'PRODH |
| 201096_s_at | 8.29 | 8.52 | 7.72 | 0.058 | 0.003 | 0.157 | 0.02 | 'ARF4 |
| 209998_at | 6.89 | 6.77 | 7.09 | 0.081 | 0.005 | 0.317 | 0.084 | 'PIGO |
| 211530_x_at | 8.48 | 9.16 | 9.13 | 0.958 | 0.904 | 0.102 | 0.008 | 'HLA-G |
| 221952_x_at | 9.58 | 9.68 | 10.04 | 0.055 | 0.002 | 0.091 | 0.007 | 'TRMT5 |
| 51200_at | 8.2 | 7.66 | 7.5 | 0.661 | 0.388 | 0.101 | 0.008 | 'C19orf60 |
| 209514_s_at | 7.22 | 7.34 | 6.7 | 0.054 | 0.002 | 0.085 | 0.006 | 'RAB27A |
| 212910_at | 6.23 | 6.26 | 6.9 | 0.042 | 0.001 | 0.057 | 0.002 | 'THAP11 |
| 210293_s_at | 6.94 | 6.64 | 6.12 | 0.231 | 0.04 | 0.054 | 0.002 | 'SEC23B |
| 200000_s_at | 8.38 | 8.54 | 9.09 | 0.017 | 0 | 0.026 | 0 | 'PRPF8 |
| 203842_s_at | 5.12 | 5.26 | 6.25 | 0.08 | 0.005 | 0.038 | 0.001 | 'MAPRE3 |
| 203110_at | 5.99 | 6.36 | 6.89 | 0.162 | 0.02 | 0.025 | 0 | 'PTK2B |
| 39249_at | 8.96 | 9.54 | 10.04 | 0.4 | 0.131 | 0.087 | 0.006 | 'AQP3 |
| 218149_s_at | 7.67 | 7.51 | 8.34 | 0.013 | 0 | 0.039 | 0.001 | 'ZNF395 |
| 216264_s_at | 6.59 | 6.45 | 7.16 | 0.052 | 0.002 | 0.119 | 0.012 | 'LAMB2 |
| 202917_s_at | 9.02 | 8.67 | 7 | 0.025 | 0 | 0.026 | 0 | 'S100A8 |
| 201395_at | 7.9 | 7.99 | 8.7 | 0.02 | 0 | 0.057 | 0.002 | 'RBM5 |
| 207856_s_at | 6.38 | 6.5 | 7.04 | 0.119 | 0.01 | 0.04 | 0.001 | 'FLJ41352|SMPD4 |
| 220661_s_at | 6.77 | 6.81 | 7.39 | 0.128 | 0.012 | 0.096 | 0.007 | 'ZNF692 |
| 214100_x_at | 6.23 | 6.39 | 7.13 | 0.128 | 0.012 | 0.09 | 0.006 | 'NSUN5B |
| 204193_at | 7.74 | 7.79 | 8.25 | 0.103 | 0.008 | 0.082 | 0.005 | 'CHKB|CPT1B |
| 214749_s_at | 8.73 | 8.56 | 8.27 | 0.244 | 0.045 | 0.053 | 0.002 | 'ARMCX6 |
| 212900_at | 6.83 | 6.87 | 6.42 | 0.08 | 0.005 | 0.112 | 0.01 | 'SEC24A |
| 210396_s_at | 6.94 | 6.43 | 5.52 | 0.27 | 0.056 | 0.09 | 0.006 | 'BOLA2|LOC595101 |
| 213976_at | 3.98 | 4.21 | 5.07 | 0.13 | 0.013 | 0.07 | 0.004 | 'CIZ1 |
| 212363_x_at | 11.89 | 12.12 | 12.49 | 0.334 | 0.088 | 0.096 | 0.008 | 'ACTG1 |
| 200845_s_at | 8.79 | 8.65 | 9.23 | 0.02 | 0 | 0.067 | 0.003 | 'PRDX6 |
| 202757_at | 7.67 | 7.22 | 7.64 | 0.011 | 0 | 0.939 | 0.857 | 'COBRA1 |
| 218321_x_at | 8.04 | 7.93 | 8.3 | 0.096 | 0.007 | 0.355 | 0.106 | 'STYXL1 |
| 220125_at | 8.61 | 8.66 | 9.26 | 0.018 | 0 | 0.047 | 0.001 | 'DNAI1 |
| 214246_x_at | 8.71 | 8.52 | 9.3 | 0.03 | 0.001 | 0.087 | 0.006 | 'MINK1 |
| 218804_at | 3.17 | 2.54 | 4.27 | 0.042 | 0.001 | 0.15 | 0.018 | 'TMEM16A |
| 41113_at | 5.33 | 5.61 | 6.06 | 0.284 | 0.063 | 0.101 | 0.008 | 'ZNF500 |
| 220744_s_at | 7.5 | 7.44 | 8.2 | 0.042 | 0.001 | 0.218 | 0.04 | 'IFT122 |
| 214620_x_at | 6.59 | 6.23 | 5.7 | 0.2 | 0.03 | 0.037 | 0.001 | 'PAM |
| 212433_x_at | 11.11 | 11.14 | 11.63 | 0.055 | 0.002 | 0.043 | 0.001 | 'RPS2 |
| 211657_at | 10.87 | 11.01 | 10.08 | 0.026 | 0.001 | 0.055 | 0.002 | 'CEACAM6 |
| 212270_x_at | 11.19 | 11.23 | 11.85 | 0.072 | 0.004 | 0.061 | 0.003 | 'RPL17 |
| 209249_s_at | 9.21 | 9.33 | 8.61 | 0.025 | 0 | 0.053 | 0.002 | 'GHITM |
| 206498_at | 5.73 | 5.74 | 4.16 | 0.074 | 0.004 | 0.074 | 0.004 | 'OCA2 |
| 203007_x_at | 6.6 | 6.78 | 5.88 | 0.063 | 0.003 | 0.146 | 0.017 | 'LYPLA1 |
| 215313_x_at | 12.31 | 12.45 | 12.93 | 0.09 | 0.006 | 0.048 | 0.002 | 'HLA-A |
| 202726_at | 6.46 | 6.17 | 6.89 | 0.031 | 0.001 | 0.074 | 0.004 | 'LIG1 |
| 206094_x_at | 7.78 | 8.08 | 7.68 | 0.1 | 0.007 | 0.822 | 0.643 | 'UGT1A6 |
| 214280_x_at | 8.08 | 8.11 | 8.44 | 0.398 | 0.129 | 0.036 | 0.001 | 'HNRNPA1 |
| 220760_x_at | 6.38 | 6.68 | 7.07 | 0.053 | 0.002 | 0.013 | 0 | 'ZNF665 |
| 213713_s_at | 8.69 | 8.6 | 9.22 | 0.098 | 0.007 | 0.154 | 0.019 | 'GLB1L2 |
| 203754_s_at | 5.38 | 5.51 | 6.48 | 0.092 | 0.007 | 0.053 | 0.002 | 'BRF1 |
| 214790_at | 3.23 | 3.4 | 4.24 | 0.047 | 0.002 | 0.023 | 0 | 'SENP6 |
| 208700_s_at | 7.76 | 8.16 | 7.53 | 0.074 | 0.004 | 0.504 | 0.223 | 'TKT |
| 211528_x_at | 10.53 | 10.91 | 11.18 | 0.482 | 0.199 | 0.053 | 0.002 | 'HLA-G |
| 214433_s_at | 9.57 | 9.57 | 10.18 | 0.026 | 0.001 | 0.031 | 0 | 'SELENBP1 |
| 203249_at | 5.74 | 5.21 | 6.74 | 0.004 | 0 | 0.047 | 0.001 | 'EZH1 |
| 212299_at | 6.84 | 6.81 | 7.47 | 0.028 | 0.001 | 0.029 | 0 | 'NEK9 |
| 214220_s_at | 7.05 | 6.5 | 6.54 | 0.901 | 0.785 | 0.07 | 0.004 | 'ALMS1 |
| 219564_at | 6.22 | 6.04 | 7.11 | 0.051 | 0.002 | 0.091 | 0.007 | 'KCNJ16 |
| 210445_at | 7.53 | 7.04 | 9.38 | 0.003 | 0 | 0.024 | 0 | 'FABP6 |
| 205437_at | 5.49 | 5.57 | 6.21 | 0.066 | 0.003 | 0.07 | 0.004 | 'ZNF211 |
| 209147_s_at | 5.5 | 5.54 | 6.19 | 0.036 | 0.001 | 0.064 | 0.003 | 'PPAP2A |
| 209971_x_at | 8.31 | 8.12 | 7.89 | 0.365 | 0.108 | 0.094 | 0.007 | 'JTV1 |
| 205596_s_at | 6.7 | 6.58 | 6.16 | 0.127 | 0.012 | 0.056 | 0.002 | 'SMURF2 |
| 53071_s_at | 7.44 | 6.71 | 6.3 | 0.408 | 0.138 | 0.043 | 0.001 | 'FLJ22222 |
| 206245_s_at | 7.44 | 7.63 | 7.19 | 0.095 | 0.007 | 0.346 | 0.101 | 'IVNS1ABP |
| 210208_x_at | 9.01 | 9.14 | 9.57 | 0.133 | 0.013 | 0.076 | 0.004 | 'BAT3 |
| 203117_s_at | 4.86 | 5.21 | 6.37 | 0.079 | 0.005 | 0.033 | 0.001 | 'PAN2 |
| 203518_at | 5.02 | 5.42 | 5.81 | 0.427 | 0.152 | 0.084 | 0.006 | 'LYST |
| 204441_s_at | 5.59 | 5.11 | 6.62 | 0.003 | 0 | 0.039 | 0.001 | 'POLA2 |
| 218248_at | 7.82 | 7.82 | 8.24 | 0.04 | 0.001 | 0.068 | 0.003 | 'FAM111A |
| 202361_at | 7.76 | 7.58 | 8.23 | 0.099 | 0.007 | 0.216 | 0.039 | 'SEC24C |
| 201891_s_at | 12.05 | 12.08 | 12.52 | 0.157 | 0.019 | 0.055 | 0.002 | 'B2M |
| 213944_x_at | 7.75 | 7.81 | 8.31 | 0.012 | 0 | 0.006 | 0 | 'GNA11 |
| 212391_x_at | 12.63 | 12.59 | 13.4 | 0.088 | 0.006 | 0.097 | 0.008 | 'RPS3A |
| 217226_s_at | 7.05 | 7 | 7.66 | 0.005 | 0 | 0.053 | 0.002 | 'SFXN3 |
| 209069_s_at | 9.78 | 10.07 | 10.62 | 0.241 | 0.044 | 0.065 | 0.003 | 'H3F3B |
| 221494_x_at | 10.05 | 9.72 | 10.17 | 0.025 | 0 | 0.591 | 0.314 | 'EIF3K |
| 219117_s_at | 8.09 | 8.18 | 7.35 | 0.022 | 0 | 0.043 | 0.001 | 'FKBP11 |
| 212273_x_at | 10.56 | 11.05 | 11.62 | 0.201 | 0.031 | 0.039 | 0.001 | 'GNAS |
| 210150_s_at | 7.26 | 7.23 | 8.15 | 0.024 | 0 | 0.032 | 0.001 | 'LAMA5 |
| 202528_at | 7.24 | 7.46 | 7.02 | 0.079 | 0.005 | 0.384 | 0.125 | 'GALE |
| 218284_at | 7.66 | 7.68 | 8 | 0.104 | 0.008 | 0.091 | 0.007 | 'SMAD3 |
| 206949_s_at | 8.4 | 8.35 | 8.07 | 0.142 | 0.015 | 0.059 | 0.003 | 'RUSC1 |
| 212848_s_at | 7.02 | 7.17 | 7.56 | 0.213 | 0.035 | 0.102 | 0.008 | 'C9orf3 |
| 212276_at | 6.16 | 6.56 | 7.1 | 0.039 | 0.001 | 0.006 | 0 | 'LPIN1 |
| 212448_at | 5.61 | 6.06 | 6.62 | 0.232 | 0.041 | 0.062 | 0.003 | 'NEDD4L |
| 212120_at | 7.6 | 7.43 | 6.88 | 0.212 | 0.035 | 0.103 | 0.009 | 'RHOQ |
| 214435_x_at | 6.5 | 6.07 | 5.26 | 0.078 | 0.005 | 0.009 | 0 | 'RALA |
| 211959_at | 8.21 | 8.18 | 9.12 | 0.04 | 0.001 | 0.121 | 0.012 | 'IGFBP5 |
| 205130_at | 8.27 | 8.19 | 8.76 | 0.005 | 0 | 0.024 | 0 | 'RAGE |
| 200986_at | 6.18 | 6.61 | 6.94 | 0.48 | 0.197 | 0.071 | 0.004 | 'SERPING1 |
| 221263_s_at | 9.68 | 9.44 | 9.15 | 0.315 | 0.078 | 0.075 | 0.004 | 'SF3B5 |
| 205489_at | 9.02 | 8.96 | 9.47 | 0.025 | 0 | 0.045 | 0.001 | 'CRYM |
| 206274_s_at | 7.28 | 7.14 | 8.42 | 0.005 | 0 | 0.027 | 0 | 'CROCC |
| 213229_at | 7.92 | 7.73 | 7.07 | 0.162 | 0.02 | 0.059 | 0.003 | 'DICER1 |
| 203221_at | 6.9 | 6.86 | 6.44 | 0.071 | 0.004 | 0.081 | 0.005 | 'TLE1 |
| 202949_s_at | 7.09 | 7.28 | 6.56 | 0.047 | 0.002 | 0.207 | 0.036 | 'FHL2 |
| 219212_at | 5.34 | 6.08 | 6.36 | 0.432 | 0.155 | 0.081 | 0.005 | 'HSPA14 |
| 213513_x_at | 9.3 | 8.95 | 8.48 | 0.215 | 0.036 | 0.047 | 0.001 | 'ARPC2 |
| 212745_s_at | 4.99 | 5.58 | 5.93 | 0.485 | 0.202 | 0.075 | 0.004 | 'BBS4 |
| 216836_s_at | 8.69 | 8.8 | 9.19 | 0.136 | 0.014 | 0.065 | 0.003 | 'ERBB2 |
| 211656_x_at | 7.43 | 8.19 | 8.44 | 0.631 | 0.351 | 0.091 | 0.007 | 'HLA-DQB1 |
| 202027_at | 7.21 | 7.08 | 6.74 | 0.19 | 0.027 | 0.081 | 0.005 | 'TMEM184B |
| 218052_s_at | 6.91 | 7.05 | 7.72 | 0.116 | 0.01 | 0.079 | 0.005 | 'ATP13A1 |
| 210148_at | 3.72 | 3.99 | 3.04 | 0.104 | 0.008 | 0.306 | 0.077 | 'HIPK3 |
| 213940_s_at | 5.26 | 5.35 | 5.99 | 0.119 | 0.01 | 0.071 | 0.004 | 'FNBP1 |
| 39248_at | 11.14 | 11.34 | 11.86 | 0.088 | 0.006 | 0.06 | 0.003 | 'AQP3 |
| 218371_s_at | 6 | 6.1 | 6.34 | 0.124 | 0.011 | 0.04 | 0.001 | 'PSPC1 |
| 208863_s_at | 6.01 | 6.77 | 6.94 | 0.728 | 0.485 | 0.094 | 0.007 | 'SFRS1 |
| 218855_at | 6.3 | 5.99 | 6.85 | 0.066 | 0.003 | 0.231 | 0.045 | 'GPR175 |
| 213195_at | 6.62 | 6.62 | 7.31 | 0.045 | 0.002 | 0.047 | 0.001 | 'LOC201229 |
| 202836_s_at | 8.95 | 8.67 | 8.51 | 0.527 | 0.239 | 0.087 | 0.006 | 'TXNL4A |
| 221041_s_at | 7.07 | 6.96 | 5.67 | 0.091 | 0.006 | 0.074 | 0.004 | 'SLC17A5 |
| 213125_at | 4.73 | 5.04 | 5.74 | 0.183 | 0.025 | 0.063 | 0.003 | 'OLFML2B |
| 203925_at | 7.16 | 7.74 | 6.7 | 0.044 | 0.002 | 0.427 | 0.155 | 'GCLM |
| 200976_s_at | 9.81 | 10.16 | 10.51 | 0.103 | 0.008 | 0.024 | 0 | 'TAX1BP1 |
| 219590_x_at | 8.25 | 7.9 | 7.87 | 0.911 | 0.808 | 0.07 | 0.004 | 'DPH5 |
| 212185_x_at | 10.76 | 10.74 | 11.36 | 0.09 | 0.006 | 0.085 | 0.006 | 'MT2A |
| 202474_s_at | 7.02 | 7.18 | 7.76 | 0.02 | 0 | 0.018 | 0 | 'HCFC1 |
| 207583_at | 4 | 3.95 | 4.93 | 0.047 | 0.002 | 0.041 | 0.001 | 'ABCD2 |
| 200755_s_at | 4.87 | 5.4 | 4.26 | 0.075 | 0.004 | 0.358 | 0.109 | 'CALU |
| 200981_x_at | 10.61 | 11.09 | 11.67 | 0.222 | 0.038 | 0.044 | 0.001 | 'GNAS |
| 207547_s_at | 6.04 | 5.87 | 7.22 | 0.001 | 0 | 0.003 | 0 | 'FAM107A |
| 212998_x_at | 6.6 | 7.42 | 8.1 | 0.402 | 0.133 | 0.091 | 0.007 | 'HLA-DQB1 |
| 200695_at | 8.1 | 8.36 | 8.6 | 0.239 | 0.043 | 0.037 | 0.001 | 'PPP2R1A |
| 209897_s_at | 6.46 | 5.75 | 6.85 | 0.008 | 0 | 0.33 | 0.091 | 'SLIT2 |
| 202599_s_at | 5.99 | 6.02 | 5.31 | 0.103 | 0.008 | 0.114 | 0.011 | 'NRIP1 |
| 209207_s_at | 6.91 | 6.89 | 6.13 | 0.057 | 0.002 | 0.039 | 0.001 | 'SEC22B |
| 213329_at | 5.45 | 5.44 | 6.5 | 0.008 | 0 | 0.01 | 0 | 'SRGAP2 |
| 215684_s_at | 6.11 | 6.48 | 6.99 | 0.216 | 0.036 | 0.057 | 0.002 | 'ASCC2 |
| 209206_at | 6.64 | 6.55 | 5.86 | 0.142 | 0.015 | 0.1 | 0.008 | 'SEC22B |
| 203973_s_at | 9.06 | 9.13 | 9.63 | 0.153 | 0.018 | 0.098 | 0.008 | 'CEBPD |
| 202437_s_at | 5.33 | 7.41 | 3.31 | 0.014 | 0 | 0.183 | 0.027 | 'CYP1B1 |
| 217841_s_at | 7.03 | 7.1 | 7.67 | 0.084 | 0.005 | 0.078 | 0.005 | 'PPME1 |
| 218255_s_at | 6.51 | 6.53 | 7.11 | 0.053 | 0.002 | 0.041 | 0.001 | 'FBRS |
| 220761_s_at | 4.77 | 5.31 | 5.66 | 0.378 | 0.117 | 0.082 | 0.005 | 'TAOK3 |
| 204034_at | 7.77 | 7.69 | 7.02 | 0.011 | 0 | 0.018 | 0 | 'ETHE1 |
| 222231_s_at | 8.17 | 8.37 | 7.85 | 0.038 | 0.001 | 0.271 | 0.06 | 'LRRC59 |
| 201923_at | 8.8 | 8.65 | 8.04 | 0.039 | 0.001 | 0.02 | 0 | 'PRDX4 |
| 37201_at | 5.65 | 5.61 | 6.31 | 0.098 | 0.007 | 0.104 | 0.009 | 'ITIH4 |
| 209806_at | 8.09 | 8.17 | 7.33 | 0.061 | 0.003 | 0.096 | 0.008 | 'HIST1H2BK |
| 219299_at | 6.85 | 6.71 | 6.35 | 0.092 | 0.006 | 0.024 | 0 | 'TRMT12 |
| 218444_at | 4.27 | 4.35 | 5.46 | 0.064 | 0.003 | 0.055 | 0.002 | 'ALG12 |
| 201214_s_at | 8.18 | 8.06 | 8.56 | 0.072 | 0.004 | 0.201 | 0.033 | 'PPP1R7 |
| 206815_at | 5.62 | 5.36 | 6.46 | 0.074 | 0.004 | 0.116 | 0.011 | 'SPAG8 |
| 214635_at | 5.7 | 5.45 | 6.34 | 0.097 | 0.007 | 0.224 | 0.042 | 'CLDN9 |
| 201697_s_at | 6.25 | 6.41 | 7.2 | 0.052 | 0.002 | 0.096 | 0.008 | 'DNMT1 |
| 44120_at | 6.95 | 6.69 | 7.12 | 0.071 | 0.004 | 0.513 | 0.231 | 'ADCK2 |
| 213839_at | 6.79 | 7.19 | 7.36 | 0.491 | 0.207 | 0.081 | 0.005 | 'KIAA0500 |
| 205363_at | 5.02 | 5.71 | 6.03 | 0.295 | 0.068 | 0.071 | 0.004 | 'BBOX1 |
| 220954_s_at | 5.47 | 5.77 | 6.68 | 0.053 | 0.002 | 0.014 | 0 | 'PILRB |
| 49452_at | 6.64 | 6.39 | 6.98 | 0.089 | 0.006 | 0.367 | 0.114 | 'ACACB |
| 202324_s_at | 7.23 | 7.34 | 6.71 | 0.081 | 0.005 | 0.15 | 0.018 | 'ACBD3 |
| 202079_s_at | 6.46 | 6.69 | 6.92 | 0.33 | 0.086 | 0.098 | 0.008 | 'TRAK1 |
| 214222_at | 7.36 | 7.41 | 8.13 | 0.024 | 0 | 0.042 | 0.001 | 'DNAH7 |
| 208398_s_at | 6.8 | 6.8 | 6.33 | 0.051 | 0.002 | 0.055 | 0.002 | 'TBPL1 |
| 205292_s_at | 8.61 | 9.03 | 9.46 | 0.289 | 0.065 | 0.06 | 0.003 | 'HNRNPA2B1 |
| 202587_s_at | 8.57 | 8.06 | 8.72 | 0.097 | 0.007 | 0.752 | 0.528 | 'AK1 |
| 217845_x_at | 8.48 | 8.66 | 7.88 | 0.071 | 0.004 | 0.153 | 0.019 | 'HIGD1A |
| 205288_at | 4.34 | 3.94 | 5.18 | 0.026 | 0.001 | 0.087 | 0.006 | 'CDC14A |
| 210058_at | 7.15 | 7.05 | 6.69 | 0.129 | 0.012 | 0.026 | 0 | 'MAPK13 |
| 209042_s_at | 6.01 | 6.03 | 6.62 | 0.029 | 0.001 | 0.051 | 0.002 | 'UBE2G2 |
| 204294_at | 7.22 | 7.04 | 7.83 | 0.033 | 0.001 | 0.044 | 0.001 | 'AMT |
| 212540_at | 7.05 | 6.67 | 7.03 | 0.081 | 0.005 | 0.956 | 0.9 | 'CDC34 |
| 209616_s_at | 10.79 | 10.27 | 11.43 | 0.075 | 0.004 | 0.189 | 0.029 | 'CES1 |
| 205322_s_at | 6.17 | 6.4 | 7.36 | 0.16 | 0.019 | 0.087 | 0.006 | 'MTF1 |
| 214169_at | 6.02 | 5.53 | 5.29 | 0.562 | 0.277 | 0.047 | 0.001 | 'UNC84A |
| 91816_f_at | 4.9 | 5.42 | 4.78 | 0.033 | 0.001 | 0.729 | 0.493 | 'MEX3D |
| 211943_x_at | 12.02 | 12.23 | 12.82 | 0.128 | 0.012 | 0.043 | 0.001 | 'TPT1 |
| 209693_at | 4.47 | 4.44 | 5.89 | 0.018 | 0 | 0.027 | 0 | 'ASTN2 |
| 201502_s_at | 9.04 | 8.91 | 9.8 | 0.037 | 0.001 | 0.079 | 0.005 | 'NFKBIA |
| 218808_at | 5.14 | 5.07 | 6.13 | 0.022 | 0 | 0.048 | 0.002 | 'DALRD3 |
| 208922_s_at | 7.48 | 7.6 | 8.2 | 0.041 | 0.001 | 0.043 | 0.001 | 'NXF1 |
| 221215_s_at | 8.65 | 8.52 | 9.06 | 0.005 | 0 | 0.053 | 0.002 | 'RIPK4 |
| 217938_s_at | 8.99 | 8.79 | 8.58 | 0.238 | 0.043 | 0.043 | 0.001 | 'KCMF1 |
| 221646_s_at | 5.38 | 6.25 | 7.84 | 0.051 | 0.002 | 0.01 | 0 | 'ZDHHC11 |
| 215085_x_at | 8.07 | 8.38 | 9.01 | 0.123 | 0.011 | 0.047 | 0.002 | 'DLEC1 |
| 210858_x_at | 6.53 | 6.36 | 5.94 | 0.271 | 0.056 | 0.091 | 0.007 | 'ATM |
| 219044_at | 6.72 | 6.17 | 7.13 | 0.043 | 0.001 | 0.199 | 0.033 | 'THNSL2 |
| 201369_s_at | 6.02 | 6.08 | 7.09 | 0.09 | 0.006 | 0.082 | 0.005 | 'ZFP36L2 |
| 218898_at | 7.52 | 7.07 | 6.82 | 0.291 | 0.066 | 0.011 | 0 | 'FAM57A |
| 203944_x_at | 7.03 | 7.37 | 7.73 | 0.16 | 0.019 | 0.023 | 0 | 'BTN2A1 |
| 210094_s_at | 7.32 | 6.9 | 7.73 | 0.03 | 0.001 | 0.261 | 0.057 | 'PARD3 |
| 219922_s_at | 5.83 | 5.59 | 7.08 | 0.004 | 0 | 0.035 | 0.001 | 'LTBP3 |
| 218831_s_at | 7.54 | 7.44 | 8.33 | 0.059 | 0.003 | 0.157 | 0.02 | 'FCGRT |
| 208300_at | 6.03 | 6.33 | 4.88 | 0.089 | 0.006 | 0.155 | 0.019 | 'PTPRH |
| 205345_at | 4.71 | 5 | 6.35 | 0.005 | 0 | 0.007 | 0 | 'BARD1 |
| 60528_at | 8.39 | 8.13 | 8.52 | 0.087 | 0.006 | 0.656 | 0.395 | 'LOC100137047-PLA2G4B |
| 212557_at | 5.9 | 6.19 | 6.59 | 0.265 | 0.054 | 0.075 | 0.004 | 'ZNF451 |
| 201289_at | 6.31 | 5.4 | 4.43 | 0.366 | 0.109 | 0.104 | 0.009 | 'CYR61 |
| 221747_at | 6.58 | 6.44 | 7.26 | 0.053 | 0.002 | 0.088 | 0.006 | 'TNS1 |
| 208950_s_at | 5.59 | 5.95 | 6.66 | 0.145 | 0.016 | 0.09 | 0.007 | 'ALDH7A1 |
| 202442_at | 8.27 | 8.22 | 7.85 | 0.285 | 0.063 | 0.057 | 0.002 | 'AP3S1 |
| 215031_x_at | 6.29 | 6.31 | 6.98 | 0.033 | 0.001 | 0.026 | 0 | 'RNF126 |
| 218095_s_at | 5.93 | 6.39 | 5.41 | 0.078 | 0.005 | 0.415 | 0.145 | 'TMEM165 |
| 208117_s_at | 6.99 | 6.33 | 7.36 | 0.079 | 0.005 | 0.534 | 0.252 | 'LAS1L |
| 218360_at | 6.35 | 6.58 | 6.18 | 0.09 | 0.006 | 0.534 | 0.252 | 'RAB22A |
| 213476_x_at | 10.52 | 10.73 | 11.21 | 0.195 | 0.029 | 0.075 | 0.004 | 'TUBB3|MC1R |
| 219248_at | 5.83 | 6.04 | 6.21 | 0.375 | 0.115 | 0.065 | 0.003 | 'THUMPD2 |
| 215892_at | 4.46 | 4.64 | 5.45 | 0.184 | 0.026 | 0.095 | 0.007 | 'ZNF440 |
| 211569_s_at | 6.63 | 6.79 | 7.25 | 0.004 | 0 | 0.057 | 0.002 | 'HADH |
| 202859_x_at | 8.92 | 8.98 | 7.7 | 0.089 | 0.006 | 0.114 | 0.011 | 'IL8 |
| 218411_s_at | 4.45 | 4.65 | 3.79 | 0.097 | 0.007 | 0.189 | 0.029 | 'MBIP |
| 201772_at | 7.03 | 7.38 | 6.68 | 0.056 | 0.002 | 0.426 | 0.154 | 'AZIN1 |
| 200038_s_at | 10.96 | 10.99 | 11.51 | 0.078 | 0.005 | 0.096 | 0.008 | 'RPL17 |
| 201358_s_at | 9.78 | 9.55 | 9.13 | 0.2 | 0.03 | 0.05 | 0.002 | 'COPB1 |
| 201318_s_at | 9.75 | 9.97 | 10.74 | 0.201 | 0.031 | 0.09 | 0.006 | 'MRLC2|MRCL3 |
| 218176_at | 6.4 | 6.74 | 7.33 | 0.144 | 0.016 | 0.08 | 0.005 | 'MAGEF1 |
| 216955_at | 6.19 | 5.3 | 6.48 | 0.028 | 0.001 | 0.585 | 0.308 | 'TAF1 |
| 219241_x_at | 7.34 | 7.33 | 7.84 | 0.002 | 0 | 0.041 | 0.001 | 'SSH3 |
| 213030_s_at | 6.65 | 6.4 | 7.18 | 0.021 | 0 | 0.045 | 0.001 | 'PLXNA2 |
| 204021_s_at | 5.91 | 6.17 | 6.95 | 0.197 | 0.029 | 0.087 | 0.006 | 'PURA |
| 202826_at | 7.91 | 8.14 | 8.7 | 0.128 | 0.012 | 0.062 | 0.003 | 'SPINT1 |
| 209106_at | 4.99 | 5.62 | 6.2 | 0.16 | 0.019 | 0.053 | 0.002 | 'NCOA1 |
| 218274_s_at | 6.78 | 6.55 | 7.19 | 0.042 | 0.001 | 0.347 | 0.102 | 'ANKZF1 |
| 216346_at | 2.54 | 2.68 | 6.27 | 0.014 | 0 | 0.017 | 0 | 'SEC14L3 |
| 219635_at | 6.65 | 6.61 | 7.14 | 0.04 | 0.001 | 0.055 | 0.002 | 'ZNF606 |
| 214175_x_at | 8.21 | 7.61 | 8.38 | 0.037 | 0.001 | 0.703 | 0.456 | 'PDLIM4 |
| 218012_at | 7.45 | 7.13 | 7.77 | 0.045 | 0.002 | 0.275 | 0.062 | 'TSPYL2 |
| 202435_s_at | 5.53 | 6.85 | 4.07 | 0.009 | 0 | 0.124 | 0.013 | 'CYP1B1 |
| 212285_s_at | 8.27 | 8.23 | 8.84 | 0.026 | 0.001 | 0.046 | 0.001 | 'AGRN |
| 211320_s_at | 7.57 | 7.58 | 8.52 | 0.032 | 0.001 | 0.037 | 0.001 | 'PTPRU |
| 205452_at | 6.21 | 6.16 | 5.7 | 0.123 | 0.011 | 0.087 | 0.006 | 'PIGB |
| 212001_at | 6.9 | 7.19 | 7.65 | 0.066 | 0.003 | 0.017 | 0 | 'SFRS14 |
| 217975_at | 7.19 | 7.19 | 6.21 | 0.103 | 0.008 | 0.096 | 0.008 | 'WBP5 |
| 210505_at | 7.4 | 8.06 | 6.63 | 0.022 | 0 | 0.18 | 0.026 | 'ADH7 |
| 201439_at | 7.04 | 7.13 | 7.5 | 0.124 | 0.011 | 0.064 | 0.003 | 'GBF1 |
| 209541_at | 4.32 | 4.78 | 3.55 | 0.104 | 0.008 | 0.301 | 0.073 | 'IGF1 |
| 201786_s_at | 8.87 | 9.17 | 9.67 | 0.07 | 0.004 | 0.014 | 0 | 'ADAR |
| 204033_at | 8.28 | 8.38 | 8.95 | 0.025 | 0 | 0.024 | 0 | 'TRIP13 |
| 215418_at | 6.87 | 7.01 | 7.68 | 0.075 | 0.004 | 0.04 | 0.001 | 'PARVA |
| 220390_at | 8.12 | 8.35 | 8.95 | 0.025 | 0 | 0.05 | 0.002 | 'AGBL2 |
| 212954_at | 5.93 | 6.07 | 6.88 | 0.036 | 0.001 | 0.031 | 0 | 'DYRK4 |
| 209149_s_at | 7.29 | 7.16 | 6.82 | 0.113 | 0.009 | 0.032 | 0.001 | 'TM9SF1 |
| 202167_s_at | 6.63 | 6.69 | 7.22 | 0.093 | 0.007 | 0.087 | 0.006 | 'MMS19 |
| 220218_at | 5 | 4.61 | 5.59 | 0.059 | 0.003 | 0.217 | 0.04 | 'C9orf68 |
| 212639_x_at | 12.3 | 12.45 | 12.84 | 0.228 | 0.04 | 0.083 | 0.006 | 'TUBA1B |
| 201904_s_at | 6.71 | 6.31 | 7.16 | 0.005 | 0 | 0.157 | 0.02 | 'CTDSPL |
| 220419_s_at | 7.19 | 7.13 | 6.87 | 0.157 | 0.018 | 0.091 | 0.007 | 'USP25 |
| 219511_s_at | 6.57 | 6.32 | 7.1 | 0.031 | 0.001 | 0.169 | 0.023 | 'SNCAIP |
| 208956_x_at | 8.82 | 8.42 | 8.42 | 0.978 | 0.946 | 0.096 | 0.008 | 'DUT |
| 212121_at | 7.62 | 7.76 | 8 | 0.08 | 0.005 | 0.096 | 0.008 | 'TCTN3 |
| 208898_at | 9.25 | 8.97 | 8.54 | 0.148 | 0.017 | 0.034 | 0.001 | 'ATP6V1D |
| 50314_i_at | 4.78 | 4.99 | 5.97 | 0.125 | 0.012 | 0.07 | 0.004 | 'C20orf27 |
| 210129_s_at | 6.37 | 6.65 | 7.55 | 0.004 | 0 | 0.017 | 0 | 'TTLL3 |
| 221743_at | 8.37 | 8.46 | 8.95 | 0.043 | 0.001 | 0.026 | 0 | 'CUGBP1 |
| 212796_s_at | 7.47 | 6.96 | 7.54 | 0.04 | 0.001 | 0.801 | 0.607 | 'TBC1D2B |
| 207367_at | 7.92 | 8.38 | 8.72 | 0.372 | 0.113 | 0.066 | 0.003 | 'ATP12A |
| 218254_s_at | 7.06 | 6.94 | 6.23 | 0.216 | 0.036 | 0.048 | 0.002 | 'SAR1B |
| 204447_at | 7.26 | 7.12 | 7.74 | 0.01 | 0 | 0.055 | 0.002 | 'ProSAPiP1 |
| 212901_s_at | 5.22 | 5.52 | 6.29 | 0.129 | 0.012 | 0.051 | 0.002 | 'CSTF2T |
| 209538_at | 6.63 | 6.47 | 7.06 | 0.074 | 0.004 | 0.05 | 0.002 | 'ZNF32 |
| 202257_s_at | 7.48 | 7.29 | 7.73 | 0.031 | 0.001 | 0.318 | 0.085 | 'CD2BP2 |
| 200781_s_at | 11.37 | 11.53 | 12.12 | 0.141 | 0.015 | 0.073 | 0.004 | 'RPS15A |
| 213460_x_at | 6.56 | 6.66 | 7.43 | 0.066 | 0.003 | 0.078 | 0.005 | 'NSUN5C |
| 201724_s_at | 7.4 | 7.31 | 6.73 | 0.128 | 0.012 | 0.079 | 0.005 | 'GALNT1 |
| 212042_x_at | 11.19 | 10.94 | 11.42 | 0.047 | 0.002 | 0.49 | 0.209 | 'RPL7 |
| 211719_x_at | 3.68 | 5.12 | 3.29 | 0.093 | 0.007 | 0.745 | 0.516 | 'FN1 |
| 200844_s_at | 9.13 | 9.11 | 9.69 | 0.079 | 0.005 | 0.169 | 0.023 | 'PRDX6 |
| 209012_at | 4.53 | 4.92 | 5.43 | 0.201 | 0.031 | 0.034 | 0.001 | 'TRIO |
| 217842_at | 5.24 | 5.11 | 5.94 | 0.052 | 0.002 | 0.108 | 0.01 | 'LUC7L2 |
| 64438_at | 6.44 | 6.11 | 5.84 | 0.375 | 0.115 | 0.069 | 0.004 | 'FLJ22222 |
| 212572_at | 6.92 | 6.65 | 6.11 | 0.357 | 0.102 | 0.086 | 0.006 | 'STK38L |
| 205812_s_at | 9.85 | 9.62 | 9.36 | 0.33 | 0.086 | 0.09 | 0.007 | 'TMED9 |
| 200019_s_at | 11.37 | 11.29 | 11.91 | 0.078 | 0.005 | 0.126 | 0.013 | 'FAU |
| 200916_at | 10.34 | 9.94 | 9.3 | 0.234 | 0.041 | 0.069 | 0.004 | 'TAGLN2 |
| 218051_s_at | 5.44 | 5.49 | 6.2 | 0.028 | 0.001 | 0.048 | 0.002 | 'NT5DC2 |
| 212646_at | 6.98 | 6.92 | 7.71 | 0.055 | 0.002 | 0.075 | 0.004 | 'RFTN1 |
| 208581_x_at | 10.07 | 10.01 | 11.02 | 0.007 | 0 | 0.018 | 0 | 'MT1X |
| 202296_s_at | 8.24 | 8.44 | 8.01 | 0.076 | 0.004 | 0.41 | 0.141 | 'RER1 |
| 203455_s_at | 10.45 | 10.26 | 9.72 | 0.142 | 0.015 | 0.081 | 0.005 | 'SAT1 |
| 209276_s_at | 7.41 | 7.95 | 7.91 | 0.914 | 0.812 | 0.081 | 0.005 | 'GLRX |
| 201118_at | 7.38 | 8.04 | 6.93 | 0.042 | 0.001 | 0.51 | 0.227 | 'PGD |
| 202245_at | 7.56 | 7.58 | 8.01 | 0.096 | 0.007 | 0.082 | 0.005 | 'LSS |
| 205258_at | 7.62 | 7.35 | 8.56 | 0.007 | 0 | 0.027 | 0 | 'INHBB |
| 201394_s_at | 7.42 | 7.7 | 8.34 | 0.058 | 0.003 | 0.024 | 0 | 'RBM5 |
| 222089_s_at | 6.02 | 6.02 | 6.8 | 0.065 | 0.003 | 0.194 | 0.031 | 'C16orf71 |
| 219461_at | 6.22 | 5.97 | 6.62 | 0.032 | 0.001 | 0.325 | 0.088 | 'PAK6 |
| 221923_s_at | 9.14 | 8.91 | 8.53 | 0.219 | 0.037 | 0.055 | 0.002 | 'NPM1 |
| 207435_s_at | 8.12 | 8.25 | 8.67 | 0.182 | 0.025 | 0.091 | 0.007 | 'SRRM2 |
| 50277_at | 9.96 | 9.81 | 10.21 | 0.07 | 0.004 | 0.308 | 0.078 | 'GGA1 |
| 218607_s_at | 7.42 | 7.3 | 6.98 | 0.233 | 0.041 | 0.104 | 0.009 | 'SDAD1 |
| 222122_s_at | 6.24 | 6.32 | 6.76 | 0.142 | 0.015 | 0.087 | 0.006 | 'THOC2 |
| 217133_x_at | 4.9 | 5.43 | 6.01 | 0.292 | 0.066 | 0.056 | 0.002 | 'CYP2B6 |
| 219583_s_at | 5.6 | 4.65 | 5.65 | 0.079 | 0.005 | 0.935 | 0.85 | 'SPATA7 |
| 212757_s_at | 6.23 | 6.43 | 6.92 | 0.173 | 0.022 | 0.054 | 0.002 | 'CAMK2G |
| 205406_s_at | 9.62 | 9.4 | 8.97 | 0.267 | 0.054 | 0.094 | 0.007 | 'SPA17 |
| 209578_s_at | 6.62 | 6.9 | 7.22 | 0.226 | 0.039 | 0.035 | 0.001 | 'POFUT2 |
| 213721_at | 7.99 | 8.22 | 8.62 | 0.246 | 0.047 | 0.07 | 0.004 | 'SOX2 |
| 222220_s_at | 8.56 | 8.34 | 8.7 | 0.1 | 0.007 | 0.607 | 0.332 | 'TSNAXIP1 |
| 59999_at | 6.53 | 6.3 | 6.73 | 0.098 | 0.007 | 0.452 | 0.176 | 'HIF1AN |
| 212573_at | 7.32 | 7.15 | 7.01 | 0.549 | 0.264 | 0.082 | 0.005 | 'ENDOD1 |
| 203954_x_at | 8.22 | 8.34 | 9.02 | 0.118 | 0.01 | 0.075 | 0.004 | 'CLDN3 |
| 201906_s_at | 7.63 | 7.17 | 7.58 | 0.033 | 0.001 | 0.868 | 0.723 | 'CTDSPL |
| 207808_s_at | 7.23 | 7.16 | 8.65 | 0.013 | 0 | 0.079 | 0.005 | 'PROS1 |
| 212936_at | 7.06 | 6.78 | 6.62 | 0.731 | 0.491 | 0.101 | 0.008 | 'FAM172A |
| 210519_s_at | 10.66 | 10.99 | 9.61 | 0.002 | 0 | 0.018 | 0 | 'NQO1 |
| 200759_x_at | 9.49 | 9.19 | 8.87 | 0.398 | 0.129 | 0.102 | 0.008 | 'NFE2L1 |
| 208306_x_at | 10.72 | 11.43 | 11.73 | 0.52 | 0.233 | 0.038 | 0.001 | 'HLA-DRB1 |
| 201298_s_at | 6.86 | 7.12 | 6.44 | 0.058 | 0.003 | 0.181 | 0.027 | 'MOBKL1B |
| 209987_s_at | 5 | 4.5 | 6.09 | 0.011 | 0 | 0.059 | 0.003 | 'ASCL1 |
| 219542_at | 7.98 | 7.91 | 8.3 | 0.049 | 0.002 | 0.172 | 0.024 | 'NEK11 |
| 218414_s_at | 8.05 | 7.85 | 8.37 | 0.047 | 0.002 | 0.33 | 0.091 | 'NDE1 |
| 220755_s_at | 7.89 | 8.08 | 8.79 | 0.055 | 0.002 | 0.058 | 0.002 | 'C6orf48 |
| 200731_s_at | 5.97 | 6.34 | 5.65 | 0.078 | 0.005 | 0.455 | 0.178 | 'PTP4A1 |
| 204177_s_at | 6.24 | 6.31 | 6.57 | 0.308 | 0.075 | 0.082 | 0.005 | 'KLHL20 |
| 220647_s_at | 6.31 | 6.64 | 7.02 | 0.194 | 0.028 | 0.027 | 0 | 'CHCHD8 |
| 204028_s_at | 8.24 | 8.08 | 8.42 | 0.055 | 0.002 | 0.271 | 0.061 | 'RABGAP1 |
| 215693_x_at | 8.53 | 8.28 | 7.95 | 0.308 | 0.075 | 0.082 | 0.005 | 'DDX27 |
| 212094_at | 2.75 | 4.03 | 4.89 | 0.149 | 0.017 | 0.006 | 0 | 'PEG10 |
| 214058_at | 7.58 | 7.14 | 7.66 | 0.026 | 0.001 | 0.72 | 0.481 | 'MYCL1 |
| 201561_s_at | 8.94 | 8.95 | 9.4 | 0.103 | 0.008 | 0.13 | 0.014 | 'CLSTN1 |
| 216396_s_at | 7.9 | 7.84 | 7.07 | 0.045 | 0.002 | 0.031 | 0 | 'EI24 |
| 212885_at | 5.65 | 5.9 | 6.52 | 0.14 | 0.015 | 0.078 | 0.005 | 'MPHOSPH10 |
| 201115_at | 7.5 | 7.48 | 7.94 | 0.097 | 0.007 | 0.109 | 0.01 | 'POLD2 |
| 219960_s_at | 6.18 | 6.36 | 5.75 | 0.066 | 0.003 | 0.261 | 0.056 | 'UCHL5 |
| 220202_s_at | 5.81 | 6.36 | 5.87 | 0.09 | 0.006 | 0.887 | 0.757 | 'RC3H2 |
| 208729_x_at | 10.77 | 11.37 | 12.09 | 0.129 | 0.012 | 0.009 | 0 | 'HLA-B |
| 217853_at | 5.36 | 5.45 | 6.63 | 0.005 | 0 | 0.033 | 0.001 | 'TNS3 |
| 203824_at | 10.25 | 10.5 | 9.9 | 0.08 | 0.005 | 0.387 | 0.126 | 'TSPAN8 |
| 55705_at | 10.24 | 9.94 | 9.61 | 0.202 | 0.031 | 0.059 | 0.003 | 'C19orf22 |
| 221290_s_at | 4.95 | 4.56 | 5.47 | 0.05 | 0.002 | 0.204 | 0.035 | 'MUM1 |
| 216268_s_at | 7.2 | 6.57 | 6.27 | 0.658 | 0.385 | 0.096 | 0.007 | 'JAG1 |
| 202501_at | 6.8 | 6.95 | 6.3 | 0.066 | 0.003 | 0.16 | 0.021 | 'MAPRE2 |
| 213266_at | 6.92 | 6.96 | 7.41 | 0.061 | 0.003 | 0.047 | 0.002 | 'TUBGCP4 |
| 91952_at | 5.87 | 5.86 | 6.61 | 0.098 | 0.007 | 0.099 | 0.008 | 'LOC90379 |
| 218139_s_at | 6.85 | 6.69 | 6.22 | 0.201 | 0.031 | 0.087 | 0.006 | 'C14orf108 |
| 202399_s_at | 8.23 | 7.98 | 7.79 | 0.491 | 0.207 | 0.096 | 0.008 | 'AP3S2 |
| 213185_at | 8.57 | 8.75 | 9.06 | 0.073 | 0.004 | 0.009 | 0 | 'KIAA0556 |
| 205012_s_at | 6.56 | 6.66 | 7.45 | 0.065 | 0.003 | 0.048 | 0.002 | 'HAGH |
| 221951_at | 5.51 | 5.59 | 6.27 | 0.061 | 0.003 | 0.067 | 0.003 | 'TMEM80 |
| 209489_at | 8.24 | 8.22 | 8.65 | 0.006 | 0 | 0.02 | 0 | 'CUGBP1 |
| 201597_at | 10.69 | 10.52 | 9.9 | 0.077 | 0.004 | 0.044 | 0.001 | 'COX7A2 |
| 213988_s_at | 9.8 | 10.06 | 8.8 | 0.078 | 0.005 | 0.077 | 0.005 | 'SAT1 |
| 208861_s_at | 7.69 | 7.5 | 7.19 | 0.355 | 0.101 | 0.103 | 0.009 | 'ATRX|LOC728849 |
| 209380_s_at | 8.17 | 8.21 | 8.66 | 0.025 | 0 | 0.009 | 0 | 'ABCC5 |
| 201844_s_at | 7.76 | 7.48 | 6.94 | 0.255 | 0.05 | 0.091 | 0.007 | 'RYBP |
| 212201_at | 5.44 | 5.78 | 6.38 | 0.113 | 0.009 | 0.024 | 0 | 'ANKLE2 |
| 206559_x_at | 12.94 | 13.18 | 14.02 | 0.146 | 0.016 | 0.07 | 0.004 | 'EEF1A1 |
| 200940_s_at | 7.65 | 7.95 | 8.31 | 0.091 | 0.006 | 0.163 | 0.022 | 'RERE |
| 203622_s_at | 6.58 | 6.6 | 6.16 | 0.079 | 0.005 | 0.131 | 0.014 | 'PNO1 |
| 205441_at | 7.2 | 7.17 | 7.61 | 0.072 | 0.004 | 0.17 | 0.024 | 'OCEL1 |
| 205513_at | 9.25 | 9.17 | 7.26 | 0.003 | 0 | 0.003 | 0 | 'TCN1 |
| 203407_at | 8.19 | 8.19 | 8.77 | 0.078 | 0.005 | 0.276 | 0.063 | 'PPL |
| 218097_s_at | 8.27 | 8.05 | 8.45 | 0.084 | 0.005 | 0.423 | 0.153 | 'CUEDC2 |
| 203140_at | 8.68 | 8.7 | 9.23 | 0.092 | 0.007 | 0.101 | 0.008 | 'BCL6 |
| 207760_s_at | 8.4 | 8.13 | 8.98 | 0.002 | 0 | 0.029 | 0 | 'NCOR2 |
| 201921_at | 7.87 | 7.44 | 7.05 | 0.28 | 0.06 | 0.037 | 0.001 | 'GNG10|LOC552891 |
| 218552_at | 7.7 | 7.77 | 8.59 | 0.003 | 0 | 0.005 | 0 | 'ECHDC2 |
| 218921_at | 6.65 | 6.67 | 7.53 | 0.015 | 0 | 0.051 | 0.002 | 'SIGIRR |
| 211732_x_at | 4.74 | 5.06 | 5.71 | 0.229 | 0.04 | 0.091 | 0.007 | 'HNMT |
| 219517_at | 7.51 | 7.21 | 6.35 | 0.075 | 0.004 | 0.032 | 0.001 | 'ELL3 |
| 205349_at | 7.12 | 7.17 | 6.59 | 0.067 | 0.003 | 0.078 | 0.005 | 'GNA15 |
| 201217_x_at | 12.67 | 12.58 | 13.2 | 0.07 | 0.004 | 0.111 | 0.01 | 'RPL3 |
| 214198_s_at | 6.96 | 6.58 | 7.27 | 0.03 | 0.001 | 0.326 | 0.088 | 'DGCR2 |
| 205417_s_at | 8.18 | 8.14 | 8.54 | 0.077 | 0.004 | 0.091 | 0.007 | 'DAG1 |
| 218383_at | 7.29 | 7.47 | 7.92 | 0.009 | 0 | 0.041 | 0.001 | 'C14orf94 |
| 207177_at | 6.1 | 5.28 | 6.68 | 0.026 | 0.001 | 0.394 | 0.131 | 'PTGFR |
| 212869_x_at | 13.12 | 13.29 | 13.98 | 0.153 | 0.018 | 0.077 | 0.005 | 'TPT1 |
| 201525_at | 7.6 | 7.52 | 8.72 | 0.011 | 0 | 0.025 | 0 | 'APOD |
| 203464_s_at | 5.96 | 5.84 | 6.89 | 0.014 | 0 | 0.032 | 0.001 | 'EPN2 |
| 218138_at | 6.51 | 6.72 | 7.28 | 0.063 | 0.003 | 0.078 | 0.005 | 'MKKS |
| 205623_at | 10.64 | 11.69 | 9.9 | 0.017 | 0 | 0.36 | 0.11 | 'ALDH3A1 |
| 202336_s_at | 8.79 | 8.68 | 7.93 | 0.083 | 0.005 | 0.076 | 0.004 | 'PAM |
| 202054_s_at | 8.37 | 8.42 | 9.15 | 0.034 | 0.001 | 0.091 | 0.007 | 'ALDH3A2 |
| 205790_at | 7.22 | 7.13 | 7.62 | 0.07 | 0.004 | 0.167 | 0.022 | 'SKAP1 |
| 212335_at | 6.63 | 6.39 | 5.87 | 0.322 | 0.082 | 0.071 | 0.004 | 'GNS |
| 204453_at | 5.13 | 5.38 | 5.93 | 0.218 | 0.036 | 0.089 | 0.006 | 'ZNF84 |
| 217804_s_at | 7.86 | 7.87 | 8.37 | 0.023 | 0 | 0.029 | 0 | 'ILF3 |
| 221577_x_at | 7.38 | 7.45 | 6.44 | 0.102 | 0.008 | 0.114 | 0.011 | 'GDF15 |
| 205328_at | 8.43 | 9.24 | 7.48 | 0.001 | 0 | 0.082 | 0.005 | 'CLDN10 |
| 204174_at | 6.92 | 7.53 | 6.65 | 0.043 | 0.001 | 0.512 | 0.23 | 'ALOX5AP |
| 219454_at | 6.71 | 6.61 | 5.98 | 0.123 | 0.011 | 0.087 | 0.006 | 'EGFL6 |
| 211034_s_at | 6.89 | 6.87 | 7.41 | 0.002 | 0 | 0.019 | 0 | 'C12orf51 |
| 201928_at | 6.97 | 6.87 | 7.47 | 0.001 | 0 | 0.005 | 0 | 'PKP4 |
| 209205_s_at | 7.87 | 8.27 | 8.82 | 0.28 | 0.06 | 0.08 | 0.005 | 'LMO4 |
| 208798_x_at | 6.66 | 7.17 | 7.83 | 0.078 | 0.005 | 0.009 | 0 | 'GOLGA8A |
| 203830_at | 3.51 | 3.59 | 4.54 | 0.123 | 0.011 | 0.087 | 0.006 | 'C17orf75 |
| 212401_s_at | 7.11 | 7.09 | 7.55 | 0.059 | 0.003 | 0.075 | 0.004 | 'CDC2L2 |
| 203694_s_at | 7.32 | 7.46 | 7.74 | 0.17 | 0.022 | 0.041 | 0.001 | 'DHX16 |
| 213629_x_at | 8.83 | 8.53 | 9.39 | 0.018 | 0 | 0.096 | 0.008 | 'MT1F |
| 54037_at | 3.78 | 4.18 | 5.25 | 0.069 | 0.004 | 0.025 | 0 | 'HPS4 |
| 211995_x_at | 11.81 | 12.22 | 12.69 | 0.32 | 0.081 | 0.055 | 0.002 | 'ACTG1 |
| 212828_at | 6.18 | 6 | 5.58 | 0.356 | 0.102 | 0.06 | 0.003 | 'SYNJ2 |
| 209129_at | 7.44 | 7.45 | 8.31 | 0.019 | 0 | 0.032 | 0.001 | 'TRIP6 |
| 203802_x_at | 6.87 | 6.91 | 7.52 | 0.053 | 0.002 | 0.182 | 0.027 | 'NSUN5 |
| 201154_x_at | 11.45 | 11.36 | 12.12 | 0.09 | 0.006 | 0.12 | 0.012 | 'RPL4 |
| 201000_at | 8.72 | 8.74 | 9.07 | 0.049 | 0.002 | 0.04 | 0.001 | 'AARS |
| 202813_at | 6.25 | 6.18 | 6.9 | 0.026 | 0.001 | 0.09 | 0.006 | 'TARBP1 |
| 212443_at | 5.44 | 5.47 | 6.35 | 0.083 | 0.005 | 0.088 | 0.006 | 'NBEAL2 |
| 217838_s_at | 6.64 | 6.81 | 7.59 | 0.003 | 0 | 0 | 0 | 'EVL |
| 205202_at | 7.38 | 7.12 | 6.55 | 0.238 | 0.043 | 0.095 | 0.007 | 'PCMT1 |
| 220911_s_at | 6.84 | 6.83 | 7.86 | 0.001 | 0 | 0.032 | 0.001 | 'KIAA1305 |
| 213217_at | 7.57 | 7.2 | 8.06 | 0.03 | 0.001 | 0.203 | 0.034 | 'ADCY2 |
| 218487_at | 4.76 | 4.89 | 5.88 | 0.036 | 0.001 | 0.061 | 0.003 | 'ALAD |
| 200798_x_at | 6.88 | 7.2 | 6.07 | 0.004 | 0 | 0.039 | 0.001 | 'MCL1 |
| 217388_s_at | 5.43 | 6.35 | 5.44 | 0.09 | 0.006 | 0.988 | 0.969 | 'KYNU |
| 209140_x_at | 12.26 | 12.47 | 12.78 | 0.336 | 0.09 | 0.059 | 0.003 | 'HLA-B |
| 218993_at | 6.66 | 6.52 | 7.06 | 0.065 | 0.003 | 0.148 | 0.018 | 'RNMTL1 |
| 216593_s_at | 4.9 | 5.44 | 5.94 | 0.308 | 0.075 | 0.055 | 0.002 | 'PIGC |
| 200631_s_at | 8.88 | 9.08 | 9.55 | 0.099 | 0.007 | 0.056 | 0.002 | 'SET |
| 200885_at | 8.55 | 8.46 | 7.96 | 0.157 | 0.018 | 0.095 | 0.007 | 'RHOC |
| 213296_at | 7.45 | 7.31 | 7.89 | 0.018 | 0 | 0.067 | 0.003 | 'RER1 |
| 218974_at | 7.75 | 7.25 | 8.05 | 0.005 | 0 | 0.271 | 0.06 | 'SOBP |
| 212266_s_at | 8.9 | 9.07 | 9.3 | 0.235 | 0.042 | 0.074 | 0.004 | 'SFRS5 |
| 209706_at | 8.89 | 8.49 | 7.87 | 0.235 | 0.042 | 0.068 | 0.003 | 'NKX3-1 |
| 219928_s_at | 6.51 | 6.77 | 5.83 | 0.079 | 0.005 | 0.192 | 0.031 | 'CABYR |
| 203901_at | 4.55 | 4.5 | 5.58 | 0.069 | 0.004 | 0.087 | 0.006 | 'MAP3K7IP1 |
| 203467_at | 7.37 | 7.02 | 8.01 | 0.017 | 0 | 0.114 | 0.011 | 'PMM1 |
| 217758_s_at | 8.33 | 8.31 | 7.48 | 0.083 | 0.005 | 0.075 | 0.004 | 'TM9SF3 |
| 214705_at | 3.76 | 4.32 | 4.92 | 0.18 | 0.025 | 0.037 | 0.001 | 'INADL |
| 200061_s_at | 11.85 | 11.82 | 12.45 | 0.092 | 0.006 | 0.089 | 0.006 | 'RPS24 |
| 206765_at | 7.05 | 7.14 | 7.62 | 0.083 | 0.005 | 0.166 | 0.022 | 'KCNJ2 |
| 219063_at | 6 | 6.2 | 6.69 | 0.21 | 0.034 | 0.086 | 0.006 | 'C1orf35 |
| 218539_at | 7.91 | 8.27 | 7.72 | 0.083 | 0.005 | 0.548 | 0.268 | 'FBXO34 |
| 205740_s_at | 7.52 | 7.35 | 7.99 | 0.041 | 0.001 | 0.136 | 0.015 | 'RBM42 |
| 212971_at | 11.17 | 10.69 | 10.41 | 0.315 | 0.079 | 0.04 | 0.001 | 'CARS |
| 221246_x_at | 6.18 | 6.13 | 6.83 | 0.057 | 0.003 | 0.068 | 0.003 | 'TNS1 |
| 219429_at | 7.43 | 7.36 | 6.85 | 0.103 | 0.008 | 0.07 | 0.004 | 'FA2H |
| 200031_s_at | 12.04 | 11.94 | 12.41 | 0.088 | 0.006 | 0.114 | 0.011 | 'RPS11 |
| 209484_s_at | 7.71 | 7.35 | 7.26 | 0.755 | 0.529 | 0.096 | 0.007 | 'NSL1 |
| 220262_s_at | 6.79 | 6.58 | 7.85 | 0.004 | 0 | 0.017 | 0 | 'DLK2 |
| 213934_s_at | 3.49 | 3.97 | 5 | 0.025 | 0.001 | 0.014 | 0 | 'ZNF19|ZNF23 |
| 212992_at | 8.6 | 8.05 | 8.79 | 0.051 | 0.002 | 0.651 | 0.387 | 'AHNAK2 |
| 219067_s_at | 6.04 | 6.29 | 6.91 | 0.047 | 0.002 | 0.026 | 0 | 'NSMCE4A |
| 213842_x_at | 8.24 | 8 | 8.79 | 0.079 | 0.005 | 0.304 | 0.075 | 'NSUN5C |
| 208270_s_at | 8.39 | 8.57 | 9.03 | 0.085 | 0.005 | 0.04 | 0.001 | 'RNPEP |
| 222030_at | 6.53 | 6.38 | 6.98 | 0.055 | 0.002 | 0.147 | 0.017 | 'SIVA1 |
| 211666_x_at | 11.28 | 11.37 | 12.03 | 0.073 | 0.004 | 0.071 | 0.004 | 'RPL3 |
| 217835_x_at | 9.62 | 9.65 | 9.24 | 0.108 | 0.009 | 0.085 | 0.006 | 'C20orf24 |
| 200026_at | 11.78 | 11.71 | 12.21 | 0.074 | 0.004 | 0.108 | 0.01 | 'RPL34 |
| 213680_at | 6.96 | 6.66 | 5.95 | 0.095 | 0.007 | 0.017 | 0 | 'KRT6B |
| 201591_s_at | 8.69 | 8.45 | 8.88 | 0.022 | 0 | 0.306 | 0.077 | 'NISCH |
| 205097_at | 7.41 | 7.4 | 6.19 | 0.023 | 0 | 0.018 | 0 | 'SLC26A2 |
| 213540_at | 7.78 | 7.53 | 8.02 | 0.07 | 0.004 | 0.379 | 0.121 | 'HSD17B8 |
| 214239_x_at | 6.52 | 6.28 | 6.93 | 0.015 | 0 | 0.214 | 0.038 | 'PCGF2 |
| 219906_at | 4.5 | 4.85 | 5.88 | 0.03 | 0.001 | 0.038 | 0.001 | 'FLJ10213 |
| 204478_s_at | 7 | 6.88 | 6.4 | 0.113 | 0.009 | 0.045 | 0.001 | 'RABIF |
| 206104_at | 7.06 | 6.5 | 7.12 | 0.052 | 0.002 | 0.883 | 0.75 | 'ISL1 |
| 221748_s_at | 7.84 | 7.39 | 7.95 | 0.049 | 0.002 | 0.723 | 0.484 | 'TNS1 |
| 201894_s_at | 6.8 | 7.38 | 6.92 | 0.09 | 0.006 | 0.827 | 0.654 | 'SSR1 |

Table S4. Gene Ontology Analysis on the over-expressed genes in smokers without cancer using high-throughput GoMiner web interface (http://discover.nci.nih.gov/gominer/), developed by National Cancer Institute, Bethesda, Maryland. The GoMiner employs two-sided Fisher's exact to compare the list of interest genes and all genes in human genome to find the GO terms that are over-represented. The input files for GoMiner include the list of our interest genes and the list of all genes in human genome. All GO evidence codes were selected. The false discovery rate (FDR) threshold was set to 0.1, and the number of randomizations was set to 1000. There were 15 GO terms were enriched, and all of them were typical molecular function terms for antioxidant response genes.

| GO term | TOTAL GENES | CHANGED GENES | ENRICHMENT | FDR q |
| --- | --- | --- | --- | --- |
| GO:0016491 oxidoreductase activity | 251 | 28 | 3.06 | 0.000 |
| GO:0016616 oxidoreductase activity acting on the CH-OH group of donors NAD or NADP as acceptor | 48 | 10 | 5.71 | 0.000 |
| GO:0004033 aldo-keto reductase activity | 10 | 5 | 13.70 | 0.001 |
| GO:0016614 oxidoreductase activity acting on CH-OH group of donors | 53 | 10 | 5.17 | 0.001 |
| GO:0016209 antioxidant activity | 15 | 5 | 9.13 | 0.007 |
| GO:0004602 glutathione peroxidase activity | 5 | 3 | 16.44 | 0.023 |
| GO:0004601 peroxidase activity | 12 | 4 | 9.13 | 0.025 |
| GO:0016684 oxidoreductase activity acting on peroxide as acceptor | 12 | 4 | 9.13 | 0.025 |
| GO:0003824 catalytic activity | 1640 | 81 | 1.35 | 0.026 |
| GO:0009055 electron carrier activity | 70 | 9 | 3.52 | 0.027 |
| GO:0004357 glutamate-cysteine ligase activity | 2 | 2 | 27.40 | 0.055 |
| GO:0031406 carboxylic acid binding | 2 | 2 | 27.40 | 0.055 |
| GO:0032052 bile acid binding | 2 | 2 | 27.40 | 0.055 |
| GO:0047115 trans-1,2-dihydrobenzene-1 2-diol dehydrogenase activity | 2 | 2 | 27.40 | 0.055 |
| GO:0016628 oxidoreductase activity acting on the CH-CH group of donors NAD or NADP as acceptor | 8 | 3 | 10.28 | 0.070 |

Table S5. Selected SNPs and genes for this study. The PWM1 and PWM2 are scores from prediction of antioxidant response element using a Position-weight Matrix model developed by our group (Wang, X. et al. Identification of polymorphic antioxidant response elements in the human genome. Hum Mol Genet, *16:* 1188-1200, 2007.). The “genTrain” score (threshold = 0.25) is a quality measure of Illumina genotyping assay; the higher the better. Tag SNPs were selected based on Hapmap genotyping data on 60 unrelated CEU individuals (U.S. Utah residents with ancestry from northern and western Europe), using the Tagger software implemented in Haploview software with r^2^ threshold of 0.8, minor allele frequency threshold of 0.1 option.

| class | rs# | alleles | chr | chrpos | symbol | allele1 | PWM1 | allele2 | PWM2 | genTrain |
| --- | --- | --- | --- | --- | --- | --- | --- | --- | --- | --- |
| ARE_SNP | rs2854470 | C/G | 10 | 4994630 | AKR1C1 | G | 7.3 | C | 3 | 0.80 |
| ARE_SNP | rs4881378 | G/T | 10 | 5054340 | AKR1C2 | C | 6.6 | A | 6.2 | 0.36 |
| ARE_SNP | rs4121280 | A/G | 10 | 5053645 | AKR1C2 | A | 12 | G | 7 | 0.67 |
| ARE_SNP | rs1937888 | A/G | 10 | 5050633 | AKR1C2 | C | 8.6 | T | 8.3 | 0.58 |
| ARE_SNP | rs10795222 | A/T | 10 | 5053145 | AKR1C2 | A | 6.2 | T | 1.3 | 0.64 |
| ARE_SNP | rs35531692 | A/T | 10 | 5124728 | AKR1C3 | A | 5.7 | T | 0.8 | 0.82 |
| ARE_SNP | rs4880708 | C/T | 10 | 5122275 | AKR1C3 | A | 6.4 | G | 1.5 | 0.76 |
| ARE_SNP | rs4645900 | A/G | 19 | 54156175 | FTL | A | 5.7 | G | 3.4 | 0.84 |
| ARE_SNP | rs11553248 | G/T | 19 | 54160519 | FTL | A | 5.6 | C | 0.7 | 0.87 |
| ARE_SNP | rs6933870 | C/G | 6 | 53505929 | GCLC | C | 6.1 | G | 5.2 | 0.77 |
| ARE_SNP | rs17885448 | A/T | 6 | 53515930 | GCLC | A | 7.8 | T | 2.9 | 0.82 |
| ARE_SNP | rs34314570 | G/T | 6 | 53522374 | GCLC | - | 5.4 | A | -5.1 | 0.83 |
| ARE_SNP | rs5755711 | C/G | 22 | 34103941 | HMOX1 | C | 10 | G | 9.6 | 0.88 |
| ARE_SNP | rs3761438 | A/C | 22 | 34104398 | HMOX1 | A | 8.9 | C | 8.3 | 0.84 |
| ARE_SNP | rs17880230 | C/T | 22 | 34104921 | HMOX1 | A | 5.5 | G | 2.1 | 0.79 |
| ARE_SNP | rs17883018 | G/T | 22 | 34108000 | HMOX1 | G | 6 | T | 1 | 0.78 |
| ARE_SNP | rs6721961 | G/T | 2 | 177838283 | NFE2L2 |  |  |  |  | 0.82 |
| ARE_SNP | rs2917667 | A/G | 16 | 68321279 | NQO1 | G | 5.2 | A | 4.6 | 0.59 |
| ARE_SNP | rs17881711 | A/G | 21 | 31952671 | SOD1 | A | 5.6 | G | 0.6 | 0.92 |
| ARE_SNP | rs202447 | A/C | 21 | 31951245 | SOD1 | C | 7.5 | A | 6.7 | 0.67 |
| ARE_SNP | rs5746086 | C/T | 6 | 160035116 | SOD2 | A | 10.3 | G | 5.4 | 0.86 |
| ARE_SNP | rs17881545 | A/G | 17 | 7529913 | TP53 | C | 9.7 | T | 4.8 | 0.82 |
| ARE_SNP | rs17885780 | A/T | 17 | 7531707 | TP53 | A | 12.4 | T | 7.5 | 0.82 |
| ARE_SNP | rs4135156 | A/G | 9 | 112058905 | TXN | A | 5.1 | G | 4.6 | 0.40 |
| ARE_SNP | rs10735393 | A/C | 12 | 103204791 | TXNRD1 | A | 6.8 | C | 4 | 0.68 |
| ARE_SNP | rs35456434 | G/T | 12 | 103206222 | TXNRD1 | C | 5 | A | 1.3 | 0.88 |
| ARE_SNP | rs504348 | C/G | 16 | 15950675 | ABCC1 | C | 5.5 | G | 1.2 | 0.64 |
| ARE_SNP | rs13011321 | A/G | 2 | 96145994 | ADRA2B | A | 5.9 | G | 2.5 | 0.85 |
| ARE_SNP | rs12532832 | A/G | 7 | 133795618 | AKR1B1 | G | 5 | A | 4.4 | 0.85 |
| ARE_SNP | rs11216157 | C/T | 11 | 116216390 | APOA1 | A | 5 | G | 0.1 | 0.79 |
| ARE_SNP | rs10896066 | A/G | 11 | 65427200 | FOSL1 | A | 5.6 | G | 4.1 | 0.80 |
| ARE_SNP | rs11554870 | A/C | 11 | 61491575 | FTH1 | A | 6.1 | C | 3.3 | 0.78 |
| ARE_SNP | rs35882418 | C/T | 22 | 23308080 | GGT1 | A | 5.1 | - | -15.5 | 0.88 |
| ARE_SNP | rs517029 | C/T | 6 | 52884810 | GSTA3 | A | 5 | G | 0 | 0.87 |
| ARE_SNP | rs11553893 | G/T | 11 | 67108594 | GSTP1 | A | 5.2 | C | 3.6 | 0.83 |
| ARE_SNP | rs34527846 | A/C | 11 | 5204608 | HBB | C | 7.5 | A | 4.2 | 0.84 |
| ARE_SNP | rs7112844 | A/T | 11 | 5208760 | HBB | A | 5.8 | T | 5.8 | 0.87 |
| ARE_SNP | rs3759067 | C/T | 11 | 5248768 | HBE1 | A | 6.4 | G | 5.2 | 0.82 |
| ARE_SNP | rs7130110 | C/G | 11 | 5252680 | HBE1 | G | 5.6 | C | 0.7 | 0.81 |
| ARE_SNP | rs1800894 | C/T | 1 | 205013289 | IL10 | A | 5.1 | G | 2.8 | 0.77 |
| ARE_SNP | rs2984914 | C/T | 1 | 59025822 | JUN | A | 13.5 | G | 11.2 | 0.72 |
| ARE_SNP | rs713358 | C/G | 1 | 45760854 | PRDX1 | C | 8 | G | 3 | 0.66 |
| ARE_SNP | rs12073047 | G/T | 1 | 45763588 | PRDX1 | C | 7.8 | A | 2.8 | 0.96 |
| ARE_SNP | rs35794739 | C/T | 1 | 171713637 | PRDX6 | C | 5.4 | T | 0.5 | 0.85 |
| ARE_SNP | rs4144740 | A/C | 14 | 57780638 | PSMA3 | A | 10.6 | C | 8 | 0.84 |
| ARE_SNP | rs689462 | A/C | 1 | 184917706 | PTGS2 | G | 5.9 | T | 1 | 0.95 |
| ARE_SNP | rs2480696 | A/G | 1 | 151773389 | S100A6 | A | 5.2 | G | 0.3 | 0.80 |
| ARE_SNP | rs28451322 | C/T | 1 | 151775387 | S100A6 | C | 12.4 | T | 12.2 | 0.79 |
| ARE_SNP | rs7682251 | A/C | 4 | 139386812 | SLC7A11 | A | 8.2 | C | 3.2 | 0.38 |
| ARE_SNP | rs11586270 | C/T | 1 | 156927456 | SPTA1 | C | 5 | T | 4.9 | 0.88 |
| ARE_SNP | rs431295 | C/T | 1 | 156922062 | SPTA1 | C | 7.3 | T | 5.3 | 0.79 |
| ARE_SNP | rs6753569 | A/C | 2 | 234260556 | UGT1A6 | G | 13.5 | T | 11.8 | 0.78 |
| ARE_SNP | rs2399888 | A/G | 5 | 10408924 | MARCH1 | A | 5 | G | 1.6 | 0.83 |
| ARE_SNP | rs1364277 | A/G | 16 | 6005575 | A2BP1 | A | 6.8 | G | 5.5 | 0.72 |
| ARE_SNP | rs11133439 | A/G | 4 | 56949092 | AASDH | C | 8.6 | T | 4.8 | 0.80 |
| ARE_SNP | rs1251078 | C/G | 1 | 75962076 | ACADM | G | 5 | C | 0 | 0.80 |
| ARE_SNP | rs6050287 | C/T | 20 | 24984327 | ACSS1 | A | 7.6 | G | 7.1 | 0.30 |
| ARE_SNP | rs8129315 | A/G | 21 | 45323291 | ADARB1 | A | 8 | G | 3.1 | 0.84 |
| ARE_SNP | rs34774688 | A/T | 4 | 100493487 | ADH1C | A | 5.9 | T | 0.9 | 0.36 |
| ARE_SNP | rs11387394 | A/G | 1 | 19512407 | AKR7A2 | - | 5.9 | C | -10.2 | 0.79 |
| ARE_SNP | rs34334833 | C/T | 1 | 19512408 | AKR7A2 | - | 5.9 | C | -10.2 | 0.62 |
| ARE_SNP | rs12122605 | C/T | 1 | 11170607 | ANGPTL7 | A | 8.4 | G | 7.2 | 0.89 |
| ARE_SNP | rs35408448 | C/G | 15 | 88151623 | ANPEP | C | 6.3 | G | 1.3 | 0.65 |
| ARE_SNP | rs34272846 | C/T | 4 | 79690985 | ANXA3 | G | 9.6 | - | 7 | 0.85 |
| ARE_SNP | rs6533825 | C/G | 4 | 79752038 | ANXA3 | C | 6.4 | G | 6.2 | 0.71 |
| ARE_SNP | rs12821083 | A/C | 12 | 97555182 | APAF1 | A | 17.4 | C | 16.9 | 0.76 |
| ARE_SNP | rs7299536 | A/G | 12 | 97625541 | APAF1 | C | 8.2 | T | 8 | 0.82 |
| ARE_SNP | rs4293461 | C/T | 18 | 22702242 | AQP4 | G | 8.3 | A | 3.4 | 0.64 |
| ARE_SNP | rs2749935 | A/T | 6 | 131931574 | ARG1 | A | 5 | T | 0 | 0.90 |
| ARE_SNP | rs4898462 | C/T | X | 152846342 | ARHGAP4 | C | 7.1 | T | 2.2 | 0.88 |
| ARE_SNP | rs4681898 | A/G | 3 | 56811780 | ARHGEF3 | G | 10 | A | 8 | 0.74 |
| ARE_SNP | rs28362548 | C/T | 3 | 132095521 | ATP2C1 | C | 11.8 | T | 11.5 | 0.79 |
| ARE_SNP | rs35555554 | C/G | 17 | 70556552 | ATP5H | G | 7.4 | C | 3.1 | 0.86 |
| ARE_SNP | rs12832882 | C/G | X | 40338168 | ATP6AP2 | G | 8.8 | C | 8.3 | 0.77 |
| ARE_SNP | rs175716 | A/G | 14 | 75055136 | BATF | A | 8.1 | G | 6.2 | 0.82 |
| ARE_SNP | rs10219508 | A/T | 12 | 24996084 | BCAT1 | A | 5.2 | T | 0.2 | 0.83 |
| ARE_SNP | rs10842436 | C/G | 12 | 24995338 | BCAT1 | G | 5.9 | C | 1 | 0.54 |
| ARE_SNP | rs2282439 | C/T | 10 | 127519125 | BCCIP | C | 6.3 | T | 5 | 0.81 |
| ARE_SNP | rs1799722 | A/G | 14 | 95740892 | BDKRB2 | A | 9.7 | G | 4.8 | 0.82 |
| ARE_SNP | rs11090141 | A/T | 22 | 41834934 | BIK | A | 5.3 | T | 0.3 | 0.84 |
| ARE_SNP | rs7822109 | C/T | 8 | 11386556 | BLK | G | 8.1 | A | 4.4 | 0.82 |
| ARE_SNP | rs230278 | C/T | 1 | 40028160 | BMP8B | C | 10.3 | T | 10.3 | 0.58 |
| ARE_SNP | rs9358933 | A/G | 6 | 26471667 | BTN3A2 | G | 5.1 | A | 4.9 | 0.83 |
| ARE_SNP | rs17880932 | A/T | 1 | 22836179 | C1QA | A | 6.6 | T | 5.8 | 0.83 |
| ARE_SNP | rs9200 | A/G | 5 | 41178363 | C6 | A | 6.8 | G | 1.9 | 0.56 |
| ARE_SNP | rs4400166 | A/G | 5 | 41224048 | C6 | C | 7.2 | T | 2.3 | 0.59 |
| ARE_SNP | rs34950983 | A/G | 7 | 93044411 | CALCR | A | 6.1 | - | 3.5 | 0.86 |
| ARE_SNP | rs12668387 | C/T | 7 | 93046331 | CALCR | G | 7.9 | A | 6.4 | 0.78 |
| ARE_SNP | rs34644384 | A/G | 5 | 149652333 | CAMK2A | C | 7 | - | 6.2 | 0.57 |
| ARE_SNP | rs8134350 | A/G | 21 | 36360511 | CBR1 | A | 5.2 | G | 4 | 0.83 |
| ARE_SNP | rs223892 | C/T | 16 | 55993654 | CCL17 | A | 5.3 | G | 4.8 | 0.94 |
| ARE_SNP | rs11861641 | A/G | 16 | 55992589 | CCL17 | C | 6.3 | T | 1.3 | 0.83 |
| ARE_SNP | rs2233872 | C/T | 9 | 34681556 | CCL19 | G | 5.7 | A | 4.3 | 0.91 |
| ARE_SNP | rs2812365 | A/G | 9 | 34653138 | CCL27 | G | 5.2 | A | 1.5 | 0.37 |
| ARE_SNP | rs1407620 | C/T | 6 | 109806052 | CD164 | C | 9.5 | T | 8 | 0.80 |
| ARE_SNP | rs16989724 | C/T | 19 | 482115 | CDC34 | A | 5.9 | G | 1 | 0.39 |
| ARE_SNP | rs10416668 | G/T | 19 | 46950976 | CEACAM6 | C | 6.2 | A | 5.1 | 0.86 |
| ARE_SNP | rs28547510 | A/G | 19 | 46947588 | CEACAM6 | C | 6 | T | 4.5 | 0.85 |
| ARE_SNP | rs12973096 | A/G | 19 | 46950754 | CEACAM6 | A | 8.7 | G | 7.6 | 0.87 |
| ARE_SNP | rs6091109 | A/G | 20 | 48239396 | CEBPB | A | 6 | G | 1 | 0.87 |
| ARE_SNP | rs6020349 | C/G | 20 | 48238975 | CEBPB | C | 8.4 | G | 3.4 | 0.82 |
| ARE_SNP | rs9859822 | C/T | 3 | 196647738 | CENTB2 | C | 6 | T | 1.1 | 0.78 |
| ARE_SNP | rs12105811 | A/G | 2 | 201724490 | CFLAR | A | 10.8 | G | 5.9 | 0.82 |
| ARE_SNP | rs11203035 | G/T | 10 | 90959915 | CH25H | C | 6.5 | A | 3.7 | 0.67 |
| ARE_SNP | rs171024 | A/G | 20 | 5836702 | CHGB | G | 5.6 | A | 5.6 | 0.84 |
| ARE_SNP | rs3829603 | G/T | 17 | 7287766 | CHRNB1 | G | 6.2 | T | 5.5 | 0.81 |
| ARE_SNP | rs4151132 | C/T | 17 | 7286765 | CHRNB1 | G | 5.7 | A | 0.7 | 0.86 |
| ARE_SNP | rs3012624 | C/T | X | 71446270 | CITED1 | G | 5.2 | A | 1.5 | 0.56 |
| ARE_SNP | rs3757460 | A/G | 7 | 100668360 | CLDN15 | A | 12.6 | G | 7.6 | 0.89 |
| ARE_SNP | rs1871461 | A/G | 5 | 157223260 | CLINT1 | A | 6.9 | G | 1.9 | 0.89 |
| ARE_SNP | rs6668576 | C/T | 1 | 158583645 | COPA | A | 7.8 | G | 6.6 | 0.87 |
| ARE_SNP | rs12446976 | C/T | 16 | 19455431 | CP110 | A | 10.7 | G | 7.3 | 0.83 |
| ARE_SNP | rs3816240 | G/T | 8 | 87636421 | CPNE3 | A | 7.6 | C | 4.8 | 0.64 |
| ARE_SNP | rs11697820 | C/T | 20 | 2729441 | CPXM1 | A | 10.2 | G | 5.3 | 0.87 |
| ARE_SNP | rs16859202 | A/G | 1 | 165792229 | CREG1 | A | 5.8 | G | 0.8 | 0.88 |
| ARE_SNP | rs2984804 | A/G | 1 | 165792445 | CREG1 | A | 8.6 | G | 7.5 | 0.87 |
| ARE_SNP | rs34185767 | C/T | 5 | 156504471 | CRSP9 | G | 6.6 | A | 5.4 | 0.94 |
| ARE_SNP | rs5760913 | A/G | 22 | 23942280 | CRYBB2 | C | 5.7 | T | 0.8 | 0.68 |
| ARE_SNP | rs1834021 | C/T | 16 | 56792268 | CSNK2A2 | A | 6.6 | G | 5.2 | 0.92 |
| ARE_SNP | rs6384 | A/G | 21 | 44018492 | CSTB | G | 9.2 | A | 9.2 | 0.88 |
| ARE_SNP | rs7768619 | C/T | 6 | 132319026 | CTGF | A | 6.6 | G | 3.2 | 0.83 |
| ARE_SNP | rs34296867 | A/G | 6 | 132314489 | CTGF | A | 5.1 | G | 3.7 | 0.84 |
| ARE_SNP | rs17131285 | C/T | 1 | 70645593 | CTH | G | 7.4 | A | 2.6 | 0.86 |
| ARE_SNP | rs3758832 | A/C | 11 | 87712046 | CTSC | G | 6.7 | T | 1.7 | 0.82 |
| ARE_SNP | rs11593858 | A/G | 10 | 35421379 | CUL2 | A | 5.9 | G | 3.7 | 0.88 |
| ARE_SNP | rs292241 | C/T | 6 | 90660226 | CX62 | A | 9.1 | G | 5.7 | 0.82 |
| ARE_SNP | rs7571060 | A/G | 2 | 38160813 | CYP1B1 | A | 5.5 | G | 5 | 0.32 |
| ARE_SNP | rs11083581 | G/T | 19 | 46080789 | CYP2A7 | A | 5.1 | C | 0.1 | 0.27 |
| ARE_SNP | rs3760657 | C/T | 19 | 46187273 | CYP2B6 | A | 10.3 | G | 9.1 | 0.82 |
| ARE_SNP | rs776740 | A/T | 7 | 99120079 | CYP3A5 | A | 12.9 | T | 8 | 0.82 |
| ARE_SNP | rs3810427 | A/C | 19 | 15907650 | CYP4F11 | A | 6.5 | C | 1.5 | 0.88 |
| ARE_SNP | rs12460831 | A/G | 19 | 15908758 | CYP4F11 | G | 9.8 | A | 8.3 | 0.78 |
| ARE_SNP | rs2280750 | A/T | 19 | 15618428 | CYP4F3 | A | 6.2 | T | 4.9 | 0.57 |
| ARE_SNP | rs10010062 | C/T | 4 | 4436130 | D4S234E | G | 6.7 | A | 1.7 | 0.82 |
| ARE_SNP | rs1148555 | A/G | 12 | 56204901 | DDIT3 | C | 5.4 | T | 0.5 | 0.78 |
| ARE_SNP | rs4699779 | A/C | 4 | 101331682 | DDIT4L | A | 6.4 | C | 4.7 | 0.83 |
| ARE_SNP | rs17047438 | C/T | 2 | 118285876 | DDX18 | G | 7 | A | 6.5 | 0.86 |
| ARE_SNP | rs2738166 | C/T | 8 | 6730439 | DEFB1 | G | 6.2 | A | 4.7 | 0.76 |
| ARE_SNP | rs6588537 | C/T | 1 | 55126781 | DHCR24 | C | 5.4 | T | 0.5 | 0.92 |
| ARE_SNP | rs3758276 | C/T | 9 | 33014917 | DNAJA1 | G | 11.2 | A | 6.3 | 0.42 |
| ARE_SNP | rs17658295 | C/T | 5 | 172134580 | DUSP1 | A | 6.3 | G | 1.3 | 0.56 |
| ARE_SNP | rs12418062 | G/T | 11 | 1551357 | DUSP8 | A | 5.2 | C | 0.2 | 0.60 |
| ARE_SNP | rs7947250 | A/C | 11 | 1553024 | DUSP8 | C | 14.1 | A | 9.2 | 0.86 |
| ARE_SNP | rs2273436 | C/T | 14 | 101500729 | DYNC1H1 | A | 8.4 | G | 3.4 | 0.39 |
| ARE_SNP | rs17390635 | C/G | 1 | 43414803 | EBNA1BP2 | C | 6.7 | G | 1.7 | 0.83 |
| ARE_SNP | rs11263839 | A/C | 1 | 36167312 | EIF2C3 | A | 7 | C | 5.9 | 0.80 |
| ARE_SNP | rs3176894 | C/T | 10 | 97504449 | ENTPD1 | G | 8.1 | A | 3.1 | 0.86 |
| ARE_SNP | rs3753660 | A/G | 1 | 224079399 | EPHX1 | G | 7.4 | A | 2.4 | 0.86 |
| ARE_SNP | rs11594945 | A/G | 10 | 50421232 | ERCC6 | A | 10.4 | G | 7 | 0.80 |
| ARE_SNP | rs4758965 | C/T | 12 | 51949002 | ESPL1 | C | 5.6 | T | 5.3 | 0.65 |
| ARE_SNP | rs17341505 | A/G | 12 | 22669111 | ETNK1 | C | 6.2 | T | 2.4 | 0.80 |
| ARE_SNP | rs10838231 | C/T | 11 | 44069549 | EXT2 | A | 8.3 | G | 3.3 | 0.64 |
| ARE_SNP | rs2655060 | C/T | 12 | 48584802 | FAIM2 | A | 5.1 | G | 0.2 | 0.86 |
| ARE_SNP | rs3761654 | G/T | 2 | 162810584 | FAP | C | 9.1 | A | 5.8 | 0.90 |
| ARE_SNP | rs4664052 | G/T | 2 | 162812146 | FAP | C | 6.9 | A | 1.9 | 0.93 |
| ARE_SNP | rs3806370 | A/G | 1 | 159787778 | FCGR3A | A | 5.4 | G | 4.1 | 0.84 |
| ARE_SNP | rs4754450 | A/G | 11 | 109802258 | FDX1 | A | 6.7 | G | 1.7 | 0.90 |
| ARE_SNP | rs2532095 | G/T | 4 | 15544858 | FGFBP1 | G | 6.5 | T | 5.6 | 0.77 |
| ARE_SNP | rs4607925 | G/T | 1 | 169549477 | FMO4 | G | 6.9 | T | 2 | 0.82 |
| ARE_SNP | rs9405496 | G/T | 6 | 1553630 | FOXC1 | G | 9 | T | 7.6 | 0.84 |
| ARE_SNP | rs1860433 | A/C | 12 | 2858205 | FOXM1 | G | 5.7 | T | 5.2 | 0.72 |
| ARE_SNP | rs11666254 | C/T | 19 | 56954974 | FPRL1 | C | 5.3 | T | 0.3 | 0.81 |
| ARE_SNP | rs3763609 | A/G | 9 | 70837230 | FXN | A | 6.6 | G | 6 | 0.84 |
| ARE_SNP | rs932374 | C/T | 1 | 207915055 | G0S2 | C | 6.2 | T | 2.5 | 0.90 |
| ARE_SNP | rs10062143 | A/G | 5 | 151127235 | G3BP1 | A | 6.7 | G | 4.8 | 0.63 |
| ARE_SNP | rs6823013 | A/G | 4 | 76800088 | G3BP2 | G | 5.3 | A | 3.8 | 0.82 |
| ARE_SNP | rs4256746 | A/G | X | 153431823 | G6PD | A | 5 | G | 2.7 | 0.78 |
| ARE_SNP | rs3791878 | A/C | 2 | 171380437 | GAD1 | G | 5 | T | 3.7 | 0.80 |
| ARE_SNP | rs2815266 | A/G | 1 | 67919581 | GADD45A | A | 5.1 | G | 0.2 | 0.74 |
| ARE_SNP | rs1555522 | C/T | 9 | 100607919 | GALNT12 | A | 6.6 | G | 5.5 | 0.86 |
| ARE_SNP | rs1543506 | C/T | 9 | 100606999 | GALNT12 | G | 10.5 | A | 9.2 | 0.80 |
| ARE_SNP | rs7652445 | G/T | 3 | 116822827 | GAP43 | G | 6.1 | T | 1.2 | 0.82 |
| ARE_SNP | rs11544794 | C/T | 21 | 33840463 | GART | A | 6.1 | G | 4.7 | 0.84 |
| ARE_SNP | rs11259842 | C/T | 13 | 113599974 | GAS6 | C | 7.9 | T | 2.9 | 0.42 |
| ARE_SNP | rs2017257 | A/T | 17 | 40352675 | GFAP | A | 5.1 | T | 2.5 | 0.80 |
| ARE_SNP | rs2070935 | G/T | 17 | 40348644 | GFAP | A | 8.8 | C | 3.8 | 0.85 |
| ARE_SNP | rs335962 | A/G | 6 | 2194126 | GMDS | A | 5.1 | G | 0.2 | 0.86 |
| ARE_SNP | rs13207508 | C/T | 6 | 2191617 | GMDS | C | 6.4 | T | 6.3 | 0.68 |
| ARE_SNP | rs10078827 | G/T | 5 | 180605459 | GNB2L1 | C | 7.2 | A | 2.2 | 0.52 |
| ARE_SNP | rs28425205 | A/G | 9 | 139157922 | GRIN1 | G | 5.7 | A | 3.7 | 0.67 |
| ARE_SNP | rs16963989 | A/G | 16 | 55523021 | HERPUD1 | A | 7.8 | G | 2.8 | 0.94 |
| ARE_SNP | rs2249571 | C/T | 6 | 29900554 | HLA-G | G | 5.6 | A | 5.4 | 0.40 |
| ARE_SNP | rs3756273 | C/T | 4 | 175681812 | HPGD | G | 6 | A | 1.2 | 0.82 |
| ARE_SNP | rs9381300 | C/T | 6 | 44321911 | HSP90AB1 | G | 10.5 | A | 9.1 | 0.83 |
| ARE_SNP | rs35258303 | C/T | 10 | 14916891 | HSPA14 | A | 6.8 | G | 4.9 | 0.84 |
| ARE_SNP | rs4757941 | C/T | 11 | 20339054 | HTATIP2 | A | 11.6 | G | 10.2 | 0.85 |
| ARE_SNP | rs6058189 | C/T | 20 | 29656050 | ID1 | A | 7.9 | G | 6.4 | 0.82 |
| ARE_SNP | rs1329588 | G/T | 9 | 130980459 | IER5L | A | 8 | C | 6.3 | 0.83 |
| ARE_SNP | rs2071204 | A/G | 3 | 50305991 | IFRD2 | A | 5.9 | G | 5.1 | 0.82 |
| ARE_SNP | rs5742612 | A/G | 12 | 101398994 | IGF1 | G | 6.9 | A | 5.4 | 0.87 |
| ARE_SNP | rs4616574 | C/T | 22 | 35879413 | IL2RB | C | 5.2 | T | 0.3 | 0.79 |
| ARE_SNP | rs2234712 | C/T | 16 | 27231929 | IL4R | A | 11.2 | G | 6.2 | 0.82 |
| ARE_SNP | rs12875067 | A/G | 13 | 110163105 | ING1 | A | 8.2 | G | 3.2 | 0.84 |
| ARE_SNP | rs2656070 | C/T | 15 | 76517307 | IREB2 | G | 5.7 | A | 0.7 | 0.78 |
| ARE_SNP | rs4728142 | A/G | 7 | 128361203 | IRF5 | A | 5.6 | G | 4.3 | 0.79 |
| ARE_SNP | rs4459557 | C/G | 16 | 31269144 | ITGAX | C | 13.8 | G | 13 | 0.66 |
| ARE_SNP | rs2829840 | A/G | 21 | 25930916 | JAM2 | A | 7.2 | G | 6 | 0.66 |
| ARE_SNP | rs10744662 | A/G | 12 | 4785469 | KCNA6 | A | 6.1 | G | 3.8 | 0.67 |
| ARE_SNP | rs701202 | C/G | 1 | 231814294 | KCNK1 | C | 8.5 | G | 3.5 | 0.87 |
| ARE_SNP | rs34676151 | C/T | 12 | 51364959 | KRT1 | G | 10.3 | - | -25.1 | 0.83 |
| ARE_SNP | rs4796695 | A/G | 17 | 36917641 | KRT13 | C | 8.1 | T | 3.2 | 0.83 |
| ARE_SNP | rs34621770 | A/T | 1 | 31006659 | LAPTM5 | A | 5.4 | T | 0.4 | 0.84 |
| ARE_SNP | rs10841872 | C/G | 12 | 21705429 | LDHB | G | 7.5 | C | 3.2 | 0.73 |
| ARE_SNP | rs7215367 | C/T | 17 | 32368225 | LHX1 | C | 7.5 | T | 7.3 | 0.80 |
| ARE_SNP | rs3766023 | C/G | 1 | 87580762 | LMO4 | G | 6 | C | 1 | 0.85 |
| ARE_SNP | rs840463 | C/T | 5 | 121446501 | LOX | C | 5.3 | T | 5 | 0.81 |
| ARE_SNP | rs840466 | G/T | 5 | 121443820 | LOX | A | 5.3 | C | 0.3 | 0.73 |
| ARE_SNP | rs3755166 | A/G | 2 | 169928127 | LRP2 | A | 9 | G | 4 | 0.85 |
| ARE_SNP | rs2256974 | G/T | 6 | 31663371 | LST1 | C | 6.7 | A | 6.1 | 0.78 |
| ARE_SNP | rs966626 | C/T | 9 | 113405849 | LTB4DH | A | 5 | G | 0.1 | 0.75 |
| ARE_SNP | rs4683236 | A/G | 3 | 46483528 | LTF | A | 12.2 | G | 7.3 | 0.60 |
| ARE_SNP | rs10732646 | C/T | 12 | 90031922 | LUM | G | 5.2 | A | 0.3 | 0.89 |
| ARE_SNP | rs2283729 | C/T | X | 43562986 | MAOB | C | 6.8 | T | 1.9 | 0.57 |
| ARE_SNP | rs11759192 | G/T | 6 | 36194954 | MAPK14 | G | 7.3 | T | 7.3 | 0.44 |
| ARE_SNP | rs2575369 | A/G | 16 | 1757432 | MAPK8IP3 | A | 7.7 | G | 6.3 | 0.74 |
| ARE_SNP | rs10459881 | C/T | 16 | 1742233 | MAPK8IP3 | A | 7 | G | 6.4 | 0.75 |
| ARE_SNP | rs3937008 | A/C | 18 | 30872891 | MAPRE2 | G | 6.5 | T | 3.3 | 0.51 |
| ARE_SNP | rs35651927 | A/G | 2 | 119411688 | MARCO | G | 7.1 | A | 2.3 | 0.83 |
| ARE_SNP | rs10414710 | G/T | 19 | 3757181 | MATK | A | 5.9 | C | 0.9 | 0.74 |
| ARE_SNP | rs35451939 | C/T | 10 | 54202034 | MBL2 | A | 10.3 | G | 5.3 | 0.88 |
| ARE_SNP | rs2384044 | C/G | 10 | 54206089 | MBL2 | C | 8.3 | G | 4 | 0.79 |
| ARE_SNP | rs6758093 | C/T | 2 | 46998366 | MCFD2 | G | 9 | A | 7.5 | 0.88 |
| ARE_SNP | rs2658718 | A/G | 12 | 94388296 | METAP2 | G | 7.6 | A | 2.7 | 0.79 |
| ARE_SNP | rs3751356 | A/G | 13 | 23363897 | MIPEP | C | 6.3 | T | 6.1 | 0.32 |
| ARE_SNP | rs9510928 | A/G | 13 | 23365994 | MIPEP | G | 5.7 | A | 0.7 | 0.69 |
| ARE_SNP | rs3864970 | A/G | 13 | 23363717 | MIPEP | A | 11.8 | G | 10.4 | 0.41 |
| ARE_SNP | rs7037941 | A/C | 9 | 20526297 | MLLT3 | A | 6.1 | C | 3.5 | 0.79 |
| ARE_SNP | rs17860944 | C/T | 11 | 102156774 | MMP10 | A | 5 | G | 0.1 | 0.87 |
| ARE_SNP | rs3848722 | C/T | 20 | 44067227 | MMP9 | A | 5 | G | 3.1 | 0.86 |
| ARE_SNP | rs13040272 | C/T | 20 | 44066518 | MMP9 | G | 8.1 | A | 6.6 | 0.80 |
| ARE_SNP | rs9622534 | C/T | 22 | 35743553 | MPST | A | 5.3 | G | 0.3 | 0.56 |
| ARE_SNP | rs10994675 | A/G | 10 | 51233999 | MSMB | C | 6.1 | T | 2.4 | 0.76 |
| ARE_SNP | rs10098474 | A/G | 8 | 9949027 | MSRA | G | 6 | A | 1.1 | 0.44 |
| ARE_SNP | rs9926088 | C/T | 16 | 55225983 | MT1A | C | 8.3 | T | 4.5 | 0.78 |
| ARE_SNP | rs10636 | C/G | 16 | 55200844 | MT2A | G | 5.2 | C | 2.6 | 0.71 |
| ARE_SNP | rs7196503 | C/T | 16 | 55198240 | MT2A | G | 5 | A | 0.1 | 0.95 |
| ARE_SNP | rs9900403 | A/C | 17 | 10266461 | MYH8 | A | 9.6 | C | 7 | 0.91 |
| ARE_SNP | rs895374 | A/C | 19 | 63758730 | MZF1 | A | 7.8 | C | 2.8 | 0.54 |
| ARE_SNP | rs1979325 | C/G | 12 | 55408203 | NACA | C | 5.7 | G | 5.6 | 0.81 |
| ARE_SNP | rs11991697 | A/G | 8 | 91069037 | NBN | G | 7.1 | A | 2.3 | 0.95 |
| ARE_SNP | rs11090572 | C/G | 22 | 28327139 | NF2 | C | 5.1 | G | 0.2 | 0.81 |
| ARE_SNP | rs35190455 | C/G | 12 | 52974791 | NFE2 | G | 8.7 | C | 4.3 | 0.77 |
| ARE_SNP | rs13082859 | A/G | 3 | 48319679 | NME6 | A | 9.1 | G | 4.1 | 0.68 |
| ARE_SNP | rs6837793 | A/G | 4 | 164476185 | NPY1R | C | 7.2 | T | 7 | 0.82 |
| ARE_SNP | rs4455659 | C/T | 6 | 24234264 | NRSN1 | A | 7.4 | G | 2.4 | 0.83 |
| ARE_SNP | rs11107001 | A/G | 12 | 92292042 | NUDT4 | A | 5.8 | G | 5.3 | 0.69 |
| ARE_SNP | rs28565256 | A/G | 3 | 9765977 | OGG1 | A | 5.7 | G | 0.8 | 0.82 |
| ARE_SNP | rs35389011 | G/T | 3 | 38181867 | OXSR1 | G | 5.3 | T | 4.7 | 0.84 |
| ARE_SNP | rs459112 | A/T | 3 | 38181580 | OXSR1 | A | 8.3 | T | 5.5 | 0.81 |
| ARE_SNP | rs11708861 | G/T | 3 | 8782460 | OXTR | G | 8.4 | T | 5.6 | 0.70 |
| ARE_SNP | rs2049050 | C/T | 19 | 18176629 | PDE4C | A | 8.4 | G | 7.1 | 0.39 |
| ARE_SNP | rs12219257 | C/T | 10 | 97041142 | PDLIM1 | C | 9 | T | 7.5 | 0.88 |
| ARE_SNP | rs4913079 | A/G | 5 | 139663213 | PFDN1 | C | 5.6 | T | 5.5 | 0.38 |
| ARE_SNP | rs12411 | A/T | 14 | 74478796 | PGF | A | 8.6 | T | 3.6 | 0.36 |
| ARE_SNP | rs1015549 | C/T | 21 | 37370750 | PIGP | G | 5.7 | A | 0.9 | 0.77 |
| ARE_SNP | rs8192349 | A/G | 15 | 70310819 | PKM2 | C | 8.7 | T | 7.9 | 0.77 |
| ARE_SNP | rs16964479 | A/G | 16 | 8796446 | PMM2 | C | 8 | T | 3.1 | 0.75 |
| ARE_SNP | rs1156770 | G/T | 8 | 82525897 | PMP2 | A | 6.5 | C | 1.6 | 0.82 |
| ARE_SNP | rs11998669 | A/G | 8 | 82523804 | PMP2 | C | 6.4 | T | 6.3 | 0.88 |
| ARE_SNP | rs2353005 | C/T | 19 | 55064574 | PNKP | A | 10.1 | G | 9 | 0.79 |
| ARE_SNP | rs2304555 | A/T | 2 | 113024987 | POLR1B | A | 9.1 | T | 7.6 | 0.83 |
| ARE_SNP | rs719667 | A/G | 2 | 113059153 | POLR1B | C | 6.5 | T | 5 | 0.69 |
| ARE_SNP | rs17885721 | C/T | 7 | 94796461 | PON1 | A | 6 | G | 1 | 0.80 |
| ARE_SNP | rs4520110 | C/G | 7 | 75416248 | POR | C | 5.9 | G | 5.5 | 0.86 |
| ARE_SNP | rs17197936 | C/T | 13 | 37073839 | POSTN | A | 9.1 | G | 7.7 | 0.90 |
| ARE_SNP | rs2472649 | C/T | 4 | 75076572 | PPBP | A | 7.4 | G | 6.3 | 0.82 |
| ARE_SNP | rs3762111 | A/C | 12 | 78853375 | PPP1R12A | A | 7.2 | C | 4.6 | 0.83 |
| ARE_SNP | rs690377 | C/T | 15 | 38910464 | PPP1R14D | A | 7 | G | 2 | 0.69 |
| ARE_SNP | rs631744 | C/G | 11 | 111145830 | PPP2R1B | C | 6.1 | G | 1.1 | 0.93 |
| ARE_SNP | rs34231866 | C/T | 19 | 12773100 | PRDX2 | A | 8.9 | G | 5.5 | 0.85 |
| ARE_SNP | rs35665723 | C/G | 10 | 120928422 | PRDX3 | G | 5.6 | C | 4.3 | 0.97 |
| ARE_SNP | rs11600990 | A/G | 11 | 63839383 | PRDX5 | A | 5.9 | G | 4.4 | 0.92 |
| ARE_SNP | rs11157930 | G/T | 14 | 22473011 | PRMT5 | C | 12.2 | A | 7.2 | 0.85 |
| ARE_SNP | rs17860796 | A/G | 6 | 170705897 | PSMB1 | G | 8.1 | A | 6.6 | 0.89 |
| ARE_SNP | rs3754982 | C/T | 2 | 231629353 | PSMD1 | G | 5.3 | A | 0.3 | 0.86 |
| ARE_SNP | rs2286728 | C/G | 12 | 6744939 | PTMS | C | 6.1 | G | 1.7 | 0.41 |
| ARE_SNP | rs7098445 | A/G | 10 | 129593392 | PTPRE | A | 7.9 | G | 6.6 | 0.80 |
| ARE_SNP | rs618369 | G/T | 1 | 43844383 | PTPRF | C | 5.2 | A | 2 | 0.64 |
| ARE_SNP | rs34836368 | G/T | 12 | 69603041 | PTPRR | - | 12.3 | A | 12 | 0.85 |
| ARE_SNP | rs2604869 | C/T | 19 | 45975533 | RAB4B | C | 7.6 | T | 6.4 | 0.82 |
| ARE_SNP | rs6690928 | C/G | 1 | 8782078 | RERE | C | 5.8 | G | 4.6 | 0.69 |
| ARE_SNP | rs6658019 | G/T | 1 | 190819425 | RGS1 | A | 5.5 | C | 4.9 | 0.65 |
| ARE_SNP | rs16858983 | A/T | 1 | 180686263 | RGSL2 | A | 10.5 | T | 5.5 | 0.77 |
| ARE_SNP | rs606661 | A/G | 1 | 180707377 | RGSL2 | A | 7 | G | 5.5 | 0.63 |
| ARE_SNP | rs3845458 | C/T | 1 | 180823075 | RNASEL | G | 8.8 | A | 5.1 | 0.87 |
| ARE_SNP | rs854921 | C/G | 6 | 90180305 | RRAGD | G | 7.4 | C | 4.3 | 0.42 |
| ARE_SNP | rs2603795 | C/T | 10 | 16900262 | RSU1 | A | 10.8 | G | 8.9 | 0.78 |
| ARE_SNP | rs4265380 | C/T | 1 | 25165943 | RUNX3 | A | 5.3 | G | 4 | 0.76 |
| ARE_SNP | rs7585590 | A/G | 2 | 20290672 | SDC1 | G | 9.2 | A | 8.4 | 0.84 |
| ARE_SNP | rs6986129 | A/T | 8 | 59624175 | SDCBP | A | 9.7 | T | 4.8 | 0.68 |
| ARE_SNP | rs12089192 | C/G | 1 | 1160885 | SDF4 | G | 6.8 | C | 1.9 | 0.83 |
| ARE_SNP | rs7127061 | C/T | 11 | 111461501 | SDHD | A | 8.1 | G | 6.6 | 0.88 |
| ARE_SNP | rs9856181 | C/T | 3 | 151749350 | SERP1 | G | 7.7 | A | 2.8 | 0.70 |
| ARE_SNP | rs2281517 | A/G | 14 | 93859540 | SERPINA6 | G | 5.8 | A | 5.8 | 0.70 |
| ARE_SNP | rs3761160 | C/T | 20 | 41620711 | SGK2 | A | 15.9 | G | 14 | 0.70 |
| ARE_SNP | rs12610409 | C/G | 19 | 44086111 | SIRT2 | C | 6.5 | G | 3.9 | 0.78 |
| ARE_SNP | rs28622444 | C/T | X | 118486059 | SLC25A5 | C | 10 | T | 5 | 0.84 |
| ARE_SNP | rs4976217 | C/T | 5 | 68437697 | SLC30A5 | A | 5.5 | G | 4.3 | 0.76 |
| ARE_SNP | rs337253 | A/C | 5 | 68458825 | SLC30A5 | A | 6.6 | C | 6.1 | 0.64 |
| ARE_SNP | rs11951414 | C/T | 5 | 68467628 | SLC30A5 | A | 12.8 | G | 12.2 | 0.64 |
| ARE_SNP | rs2581437 | A/G | 4 | 41683774 | SLC30A9 | A | 7 | G | 5.6 | 0.76 |
| ARE_SNP | rs6837931 | A/T | 4 | 72564097 | SLC4A4 | A | 5.4 | T | 3.7 | 0.59 |
| ARE_SNP | rs13092196 | A/C | 3 | 11009004 | SLC6A1 | C | 6.6 | A | 1.6 | 0.87 |
| ARE_SNP | rs5021960 | A/G | 22 | 29803891 | SMTN | A | 6.7 | G | 4.8 | 0.59 |
| ARE_SNP | rs6882787 | C/G | 5 | 122136610 | SNX2 | G | 5.4 | C | 0.4 | 0.74 |
| ARE_SNP | rs16853859 | A/C | 1 | 177528033 | SOAT1 | C | 5.5 | A | 5.5 | 0.88 |
| ARE_SNP | rs7527646 | A/G | 1 | 54645697 | SSBP3 | A | 8 | G | 6.5 | 0.91 |
| ARE_SNP | rs2343869 | A/G | 12 | 26238269 | SSPN | C | 5.7 | T | 1.9 | 0.72 |
| ARE_SNP | rs6978286 | A/G | 7 | 116404768 | ST7 | C | 6.2 | T | 1.2 | 0.71 |
| ARE_SNP | rs4796793 | C/G | 17 | 37795736 | STAT3 | G | 9.4 | C | 5.1 | 0.80 |
| ARE_SNP | rs2187827 | C/T | 6 | 36567444 | STK38 | A | 5 | G | 3.4 | 0.88 |
| ARE_SNP | rs7524624 | A/T | 1 | 27966928 | STX12 | A | 12.5 | T | 7.5 | 0.92 |
| ARE_SNP | rs11608466 | A/C | 12 | 129891314 | STX2 | A | 7.9 | C | 2.9 | 0.80 |
| ARE_SNP | rs6802933 | A/T | 3 | 12020146 | SYN2 | A | 6.2 | T | 3.6 | 0.82 |
| ARE_SNP | rs2618401 | A/G | 3 | 12017001 | SYN2 | C | 8.4 | T | 8.3 | 0.57 |
| ARE_SNP | rs2072684 | C/G | 16 | 1975416 | SYNGR3 | G | 5.7 | C | 0.7 | 0.72 |
| ARE_SNP | rs2075547 | A/C | 11 | 116573744 | TAGLN | G | 6.9 | T | 5.6 | 0.94 |
| ARE_SNP | rs11079786 | C/T | 17 | 43160915 | TBX21 | A | 5.5 | G | 0.5 | 0.78 |
| ARE_SNP | rs615532 | A/G | 9 | 27094709 | TEK | C | 7.7 | T | 5.7 | 0.67 |
| ARE_SNP | rs9631470 | A/T | 3 | 101908709 | TFG | A | 9.7 | T | 8.4 | 0.70 |
| ARE_SNP | rs1052897 | A/T | 7 | 100078707 | TFR2 | A | 7.3 | T | 2.4 | 0.80 |
| ARE_SNP | rs6583288 | A/G | 3 | 197293961 | TFRC | C | 6.8 | T | 1.8 | 0.78 |
| ARE_SNP | rs6589737 | C/T | 11 | 118800857 | THY1 | G | 5.1 | A | 0.2 | 0.44 |
| ARE_SNP | rs10515193 | C/T | 5 | 74094847 | TINP1 | G | 6.7 | A | 1.7 | 0.82 |
| ARE_SNP | rs28171 | A/G | 16 | 65128922 | TK2 | A | 5.8 | G | 4.6 | 0.80 |
| ARE_SNP | rs5743818 | G/T | 4 | 38505558 | TLR6 | A | 6.5 | C | 4.8 | 0.82 |
| ARE_SNP | rs17207127 | A/G | 6 | 31649927 | TNF | A | 6.8 | G | 5.3 | 0.95 |
| ARE_SNP | rs9282875 | A/G | 6 | 31650245 | TNF | A | 5 | G | 3.8 | 0.80 |
| ARE_SNP | rs2274227 | A/C | 1 | 177318629 | TOR3A | G | 7.8 | T | 7.8 | 0.81 |
| ARE_SNP | rs1863438 | A/G | 8 | 81220414 | TPD52 | G | 5.1 | A | 3.9 | 0.60 |
| ARE_SNP | rs2071402 | A/G | 2 | 1396206 | TPO | A | 10.3 | G | 9.1 | 0.79 |
| ARE_SNP | rs11686524 | G/T | 2 | 1391251 | TPO | C | 7.7 | A | 4 | 0.64 |
| ARE_SNP | rs4390325 | G/T | 11 | 18499446 | TSG101 | A | 8 | C | 7 | 0.79 |
| ARE_SNP | rs1110400 | A/G | 16 | 88513631 | TUBB3 | G | 10.1 | A | 8.9 | 0.83 |
| ARE_SNP | rs34490506 | C/T | 16 | 88513884 | TUBB3 | C | 7.9 | T | 2.9 | 0.88 |
| ARE_SNP | rs9622402 | G/T | 22 | 35209990 | TXN2 | A | 10.6 | C | 5.6 | 0.77 |
| ARE_SNP | rs362511 | A/G | 6 | 29638829 | UBD | G | 5.1 | A | 4.3 | 0.75 |
| ARE_SNP | rs769052 | C/T | 5 | 138924617 | UBE2D2 | G | 5.9 | A | 5.7 | 0.50 |
| ARE_SNP | rs7687015 | G/T | 4 | 141713327 | UCP1 | A | 13.5 | C | 10.7 | 0.81 |
| ARE_SNP | rs3783598 | A/C | 1 | 100956406 | VCAM1 | A | 6.6 | C | 1.7 | 0.75 |
| ARE_SNP | rs3731055 | A/G | 3 | 14195443 | XPC | C | 5.8 | T | 0.8 | 0.82 |
| ARE_SNP | rs3731056 | A/T | 3 | 14195191 | XPC | A | 7.4 | T | 2.4 | 0.82 |
| tagSNP | rs2904803 | C/T | 10 | 4993355 | AKR1C1 |  |  |  |  | 0.56 |
| tagSNP | rs6601882 | C/T | 10 | 5011218 | AKR1C1 |  |  |  |  | 0.70 |
| tagSNP | rs4596966 | A/G | 10 | 4989304 | AKR1C1 |  |  |  |  | 0.69 |
| tagSNP | rs3890593 | A/G | 10 | 5005854 | AKR1C1 |  |  |  |  | 0.66 |
| tagSNP | rs7909151 | C/T | 10 | 5016096 | AKR1C1 |  |  |  |  | 0.74 |
| tagSNP | rs2904804 | G/T | 10 | 4999759 | AKR1C1 |  |  |  |  | 0.67 |
| tagSNP | rs4143630 | A/G | 10 | 5053944 | AKR1C2 |  |  |  |  | 0.50 |
| tagSNP | rs10904392 | A/C | 10 | 5058113 | AKR1C2 |  |  |  |  | 0.82 |
| tagSNP | rs12414884 | G/T | 10 | 5053398 | AKR1C2 |  |  |  |  | 0.49 |
| tagSNP | rs12261648 | G/T | 10 | 5054317 | AKR1C2 |  |  |  |  | 0.61 |
| tagSNP | rs17134158 | A/G | 10 | 5055166 | AKR1C2 |  |  |  |  | 0.69 |
| tagSNP | rs2895059 | A/G | 10 | 5055747 | AKR1C2 |  |  |  |  | 0.71 |
| tagSNP | rs2801883 | A/G | 10 | 5129003 | AKR1C3 |  |  |  |  | 0.60 |
| tagSNP | rs10904415 | A/G | 10 | 5126203 | AKR1C3 |  |  |  |  | 0.63 |
| tagSNP | rs12263243 | A/T | 10 | 5120783 | AKR1C3 |  |  |  |  | 0.45 |
| tagSNP | rs11252932 | A/G | 10 | 5121180 | AKR1C3 |  |  |  |  | 0.67 |
| tagSNP | rs1937849 | G/T | 10 | 5123839 | AKR1C3 |  |  |  |  | 0.59 |
| tagSNP | rs1937841 | A/G | 10 | 5131289 | AKR1C3 |  |  |  |  | 0.59 |
| tagSNP | rs4881400 | A/C | 10 | 5134037 | AKR1C3 |  |  |  |  | 0.76 |
| tagSNP | rs1937924 | C/G | 10 | 5148682 | AKR1C3 |  |  |  |  | 0.50 |
| tagSNP | rs1805419 | A/G | 19 | 54150916 | FTL|BAX |  |  |  |  | 0.75 |
| tagSNP | rs2230267 | C/T | 19 | 54160899 | FTL|BAX |  |  |  |  | 0.29 |
| tagSNP | rs2270938 | A/T | 19 | 54165839 | FTL|BAX |  |  |  |  | 0.69 |
| tagSNP | rs2397146 | A/G | 6 | 53468078 | GCLC |  |  |  |  | 0.74 |
| tagSNP | rs10948751 | G/T | 6 | 53469359 | GCLC |  |  |  |  | 0.88 |
| tagSNP | rs12525474 | A/G | 6 | 53474020 | GCLC |  |  |  |  | 0.72 |
| tagSNP | rs670548 | A/G | 6 | 53474948 | GCLC |  |  |  |  | 0.92 |
| tagSNP | rs661603 | C/T | 6 | 53478066 | GCLC |  |  |  |  | 0.83 |
| tagSNP | rs524553 | C/T | 6 | 53478354 | GCLC |  |  |  |  | 0.82 |
| tagSNP | rs2100375 | A/G | 6 | 53493434 | GCLC |  |  |  |  | 0.83 |
| tagSNP | rs3799696 | C/T | 6 | 53504855 | GCLC |  |  |  |  | 0.95 |
| tagSNP | rs502862 | A/G | 6 | 53506518 | GCLC |  |  |  |  | 0.70 |
| tagSNP | rs510088 | A/G | 6 | 53516748 | GCLC |  |  |  |  | 0.83 |
| tagSNP | rs2235970 | C/T | 1 | 94117725 | GCLM |  |  |  |  | 0.84 |
| tagSNP | rs6687387 | C/T | 1 | 94118915 | GCLM |  |  |  |  | 0.91 |
| tagSNP | rs2301022 | C/T | 1 | 94145466 | GCLM |  |  |  |  | 0.86 |
| tagSNP | rs3827715 | C/T | 1 | 94142211 | GCLM |  |  |  |  | 0.84 |
| tagSNP | rs2978663 | C/T | 8 | 30685487 | GSR |  |  |  |  | 0.26 |
| tagSNP | rs2978295 | C/G | 8 | 30695416 | GSR |  |  |  |  | 0.82 |
| tagSNP | rs2250192 | A/C | 8 | 30659165 | GSR |  |  |  |  | 0.87 |
| tagSNP | rs3594 | A/C | 8 | 30655202 | GSR |  |  |  |  | 0.87 |
| tagSNP | rs2551715 | C/T | 8 | 30666178 | GSR |  |  |  |  | 0.66 |
| tagSNP | rs8190996 | A/G | 8 | 30673548 | GSR |  |  |  |  | 0.85 |
| tagSNP | rs735266 | A/T | 22 | 34098063 | HMOX1 |  |  |  |  | 0.65 |
| tagSNP | rs2071749 | C/T | 22 | 34113413 | HMOX1 |  |  |  |  | 0.84 |
| tagSNP | rs5755720 | C/T | 22 | 34116873 | HMOX1 |  |  |  |  | 0.88 |
| tagSNP | rs743811 | A/G | 22 | 34122974 | HMOX1 |  |  |  |  | 0.66 |
| tagSNP | rs743815 | C/T | 22 | 34127333 | HMOX1 |  |  |  |  | 0.79 |
| tagSNP | rs737777 | G/T | 22 | 34128046 | HMOX1 |  |  |  |  | 0.66 |
| tagSNP | rs713873 | C/T | 22 | 34129611 | HMOX1 |  |  |  |  | 0.82 |
| tagSNP | rs7246953 | C/T | 19 | 10482108 | KEAP1 |  |  |  |  | 0.84 |
| tagSNP | rs1048290 | C/G | 19 | 10461442 | KEAP1 |  |  |  |  | 0.76 |
| tagSNP | rs10409101 | C/G | 19 | 10482208 | KEAP1 |  |  |  |  | 0.94 |
| tagSNP | rs4243387 | C/T | 2 | 177826011 | NFE2L2 |  |  |  |  | 0.78 |
| tagSNP | rs13001694 | A/G | 2 | 177827236 | NFE2L2 |  |  |  |  | 0.85 |
| tagSNP | rs2886161 | C/T | 2 | 177836085 | NFE2L2 |  |  |  |  | 0.92 |
| tagSNP | rs7557529 | C/T | 2 | 177843343 | NFE2L2 |  |  |  |  | 0.82 |
| tagSNP | rs16865105 | A/C | 2 | 177844875 | NFE2L2 |  |  |  |  | 0.91 |
| tagSNP | rs9068 | A/G | 2 | 177793383 | NFE2L2 |  |  |  |  | 0.86 |
| tagSNP | rs13029183 | C/T | 2 | 177796097 | NFE2L2 |  |  |  |  | 0.74 |
| tagSNP | rs2917666 | C/G | 16 | 68321461 | NQO1 |  |  |  |  | 0.74 |
| tagSNP | rs12232410 | A/G | 16 | 68294352 | NQO1 |  |  |  |  | 0.93 |
| tagSNP | rs1469908 | A/G | 16 | 68321913 | NQO1 |  |  |  |  | 0.78 |
| tagSNP | rs7359387 | A/C | 16 | 68291166 | NQO1 |  |  |  |  | 0.90 |
| tagSNP | rs7359336 | A/G | 16 | 68290961 | NQO1 |  |  |  |  | 0.81 |
| tagSNP | rs10517 | A/G | 16 | 68301261 | NQO1 |  |  |  |  | 0.69 |
| tagSNP | rs202449 | A/T | 21 | 31968154 | SOD1 |  |  |  |  | 0.68 |
| tagSNP | rs10432782 | A/C | 21 | 31958262 | SOD1 |  |  |  |  | 0.65 |
| tagSNP | rs1041740 | C/T | 21 | 31962033 | SOD1 |  |  |  |  | 0.81 |
| tagSNP | rs4342445 | C/T | 6 | 160018212 | SOD2 |  |  |  |  | 0.69 |
| tagSNP | rs2758329 | A/G | 6 | 160018273 | SOD2 |  |  |  |  | 0.80 |
| tagSNP | rs7855 | C/T | 6 | 160020292 | SOD2 |  |  |  |  | 0.80 |
| tagSNP | rs8031 | A/T | 6 | 160020630 | SOD2 |  |  |  |  | 0.92 |
| tagSNP | rs5746136 | C/T | 6 | 160023074 | SOD2 |  |  |  |  | 0.78 |
| tagSNP | rs5746081 | A/G | 6 | 160035444 | SOD2 |  |  |  |  | 0.80 |
| tagSNP | rs2758352 | A/G | 6 | 160042911 | SOD2 |  |  |  |  | 0.51 |
| tagSNP | rs8073498 | A/C | 17 | 7510423 | TP53 |  |  |  |  | 0.81 |
| tagSNP | rs12951053 | G/T | 17 | 7518132 | TP53 |  |  |  |  | 0.83 |
| tagSNP | rs12602273 | C/G | 17 | 7523738 | TP53 |  |  |  |  | 0.84 |
| tagSNP | rs2287497 | C/T | 17 | 7533505 | TP53 |  |  |  |  | 0.80 |
| tagSNP | rs10759402 | C/T | 9 | 112040034 | TXN |  |  |  |  | 0.75 |
| tagSNP | rs4135233 | C/T | 9 | 112045138 | TXN |  |  |  |  | 0.85 |
| tagSNP | rs2776 | A/G | 9 | 112046109 | TXN |  |  |  |  | 0.81 |
| tagSNP | rs4135225 | C/T | 9 | 112046512 | TXN |  |  |  |  | 0.83 |
| tagSNP | rs4135221 | A/G | 9 | 112047058 | TXN |  |  |  |  | 0.68 |
| tagSNP | rs4135215 | C/T | 9 | 112047320 | TXN |  |  |  |  | 0.75 |
| tagSNP | rs4135212 | A/G | 9 | 112048244 | TXN |  |  |  |  | 0.78 |
| tagSNP | rs4135208 | C/T | 9 | 112049067 | TXN |  |  |  |  | 0.84 |
| tagSNP | rs4135203 | C/G | 9 | 112050671 | TXN |  |  |  |  | 0.91 |
| tagSNP | rs2418076 | A/G | 9 | 112053172 | TXN |  |  |  |  | 0.88 |
| tagSNP | rs4135192 | A/G | 9 | 112053409 | TXN |  |  |  |  | 0.52 |
| tagSNP | rs4135188 | A/G | 9 | 112054548 | TXN |  |  |  |  | 0.81 |
| tagSNP | rs4135182 | G/T | 9 | 112055641 | TXN |  |  |  |  | 0.83 |
| tagSNP | rs4135168 | A/G | 9 | 112056706 | TXN |  |  |  |  | 0.83 |
| tagSNP | rs1410051 | C/T | 9 | 112057173 | TXN |  |  |  |  | 0.85 |
| tagSNP | rs4135165 | A/G | 9 | 112057383 | TXN |  |  |  |  | 0.68 |
| tagSNP | rs2301242 | A/T | 9 | 112058823 | TXN |  |  |  |  | 0.33 |
| tagSNP | rs2301241 | A/G | 9 | 112059329 | TXN |  |  |  |  | 0.71 |
| tagSNP | rs3808888 | A/G | 9 | 112059975 | TXN |  |  |  |  | 0.83 |
| tagSNP | rs10778318 | C/T | 12 | 103195636 | TXNRD1 |  |  |  |  | 0.83 |
| tagSNP | rs4564401 | A/G | 12 | 103227358 | TXNRD1 |  |  |  |  | 0.82 |
| tagSNP | rs5018287 | A/G | 12 | 103231281 | TXNRD1 |  |  |  |  | 0.91 |
| tagSNP | rs17202060 | A/G | 12 | 103254976 | TXNRD1 |  |  |  |  | 0.84 |
| tagSNP | rs7138318 | C/T | 12 | 103260524 | TXNRD1 |  |  |  |  | 0.85 |
| tagSNP | rs10861203 | A/G | 12 | 103260828 | TXNRD1 |  |  |  |  | 0.72 |
| tagSNP | rs7977617 | G/T | 12 | 103271967 | TXNRD1 |  |  |  |  | 0.83 |
| tagSNP | rs7977553 | C/T | 12 | 103272116 | TXNRD1 |  |  |  |  | 0.77 |
| tagSNP | rs17808695 | C/T | 12 | 103277344 | TXNRD1 |  |  |  |  | 0.89 |

Table S6. Novel SNPs discovered by sequencing the MAFG locus (chr17:77,467,438-77,483,879). The offset to MAFG mRNA NM_002359 or NM_032711 was annotated as “offset_TSS1” or “offset_SS2” respectively. The minor allele frequency (MAF), Hardy-Weinberg (HW) test, genotyped rate, and SNP ID in dbSNP if available, were listed.

| chrPos | offset2TSS1* | offset2TSS2** | LOCATION | MAF | HW p-value | %Genotyped | Alleles | dbSNP |
| --- | --- | --- | --- | --- | --- | --- | --- | --- |
| 77482956 | -4077 | -8255 | upstream | 0.093 | 0.601 | 97.7 | C:A |  |
| 77482914 | -4035 | -8213 | upstream | 0.035 | 1.000 | 97.7 | C:A |  |
| 77481210 | -2331 | -6509 | upstream | 0.333 | 0.465 | 95.5 | G:C |  |
| 77481166 | -2287 | -6465 | upstream | 0.014 | 1.000 | 79.5 | T:C |  |
| 77481092 | -2213 | -6391 | upstream | 0.243 | 0.151 | 79.5 | C:G |  |
| 77481072 | -2193 | -6371 | upstream | 0.057 | 1.000 | 79.5 | G:A |  |
| 77481067 | -2188 | -6366 | upstream | 0.043 | 1.000 | 79.5 | C:G |  |
| 77481062 | -2183 | -6361 | upstream | 0.043 | 1.000 | 79.5 | G:A |  |
| 77480590 | -1711 | -5889 | upstream | 0.043 | 1.000 | 79.5 | T:C |  |
| 77480336 | -1457 | -5635 | upstream | 0.034 | 1.000 | 100 | C:T |  |
| 77480108 | -1229 | -5407 | upstream | 0.023 | 1.000 | 100 | C:T |  |
| 77479859 | -980 | -5158 | upstream | 0.057 | 1.000 | 79.5 | T:C | rs74006139 |
| 77479711 | -832 | -5010 | upstream | 0.015 | 1.000 | 75 | C:T |  |
| 77477948 | 931 | -3247 | INTRON|upstream | 0.057 | 1.000 | 79.5 | T:A |  |
| 77475689 | 3190 | -988 | INTRON|upstream | 0.014 | 1.000 | 79.5 | A:G |  |
| 77474440 | 4439 | 261 | INTRON | 0.014 | 1.000 | 79.5 | G:T |  |
| 77473857 | 5022 | 844 | CDS | 0.011 | 1.000 | 100 | C:T | rs61752690 |
| 77473338 | 5541 | 1363 | UTR3 | 0.014 | 1.000 | 79.5 | T:C |  |
| 77472128 | 6751 | 2573 | UTR3 | 0.011 | 1.000 | 100 | C:T |  |
| 77472034 | 6845 | 2667 | UTR3 | 0.167 | 0.604 | 88.6 | C:T | rs3744810 |
| 77470790 | 8089 | 3911 | UTR3 | 0.015 | 1.000 | 75 | G:T |  |
| 77470314 | 8565 | 4387 | UTR3 | 0.267 | 0.034 | 97.7 | T:C |  |
| 77470207 | 8672 | 4494 | UTR3 | 0.273 | 0.025 | 100 | A:G |  |
| 77470171 | 8708 | 4530 | UTR3 | 0.295 | 0.010 | 100 | A:C |  |
| 77470112 | 8767 | 4589 | UTR3 | 0.150 | 0.003 | 90.9 | C:A |  |
| 77469872 | 9007 | 4829 | UTR3 | 0.488 | 0.048 | 93.2 | C:G |  |
| 77469864 | 9015 | 4837 | UTR3 | 0.420 | 1.000 | 100 | A:G |  |
| 77469782 | 9097 | 4919 | UTR3 | 0.291 | 0.013 | 97.7 | A:C |  |
| 77469779 | 9100 | 4922 | UTR3 | 0.102 | 1.000 | 100 | G:A |  |
| 77469704 | 9175 | 4997 | UTR3 | 0.238 | 0.119 | 90.9 | G:A |  |
| 77469673 | 9206 | 5028 | UTR3 | 0.014 | 1.000 | 79.5 | C:A |  |
| 77469644 | 9235 | 5057 | UTR3 | 0.141 | 0.883 | 88.6 | A:T |  |
| 77469636 | 9243 | 5065 | UTR3 | 0.227 | 0.120 | 100 | A:G |  |

Table S7. The mRNA level of MAFG gene (probe ID: 204970_s_at) was examined in a larger, related dataset (Gene Expression Omnibus accession number GSE4115), that was previously published (Spira, A. *et al.* Nature Medicine. 2007. 13, 361-366). In that dataset, the average log2 expression of MAFG was significantly greater in smokers without cancer compared to smoker with cancer.

|  | MAFG (204970_s_at)  Mean log2 expression | t-test |
| --- | --- | --- |
| Smokers Without Cancer (n = 97) | 6.890 | p= 0.0039 |
| Smokers With Cancer(n=90) | 6.768 |

Figure S1A. The linkage disequilibrium (LD) plot of MAFG SNPs. These SNPs were discovered by resequencing the MAFG locus and the LD plot (bottom part) was generated by the Haploview software. The numeric value within each block is the r^2^ value (expressed as a percent), which measures the correlation between alleles of two SNPs. SNPs and their chromosomal positions are displayed above the LD plot and two alternatively spliced forms of MAFG gene are shown.


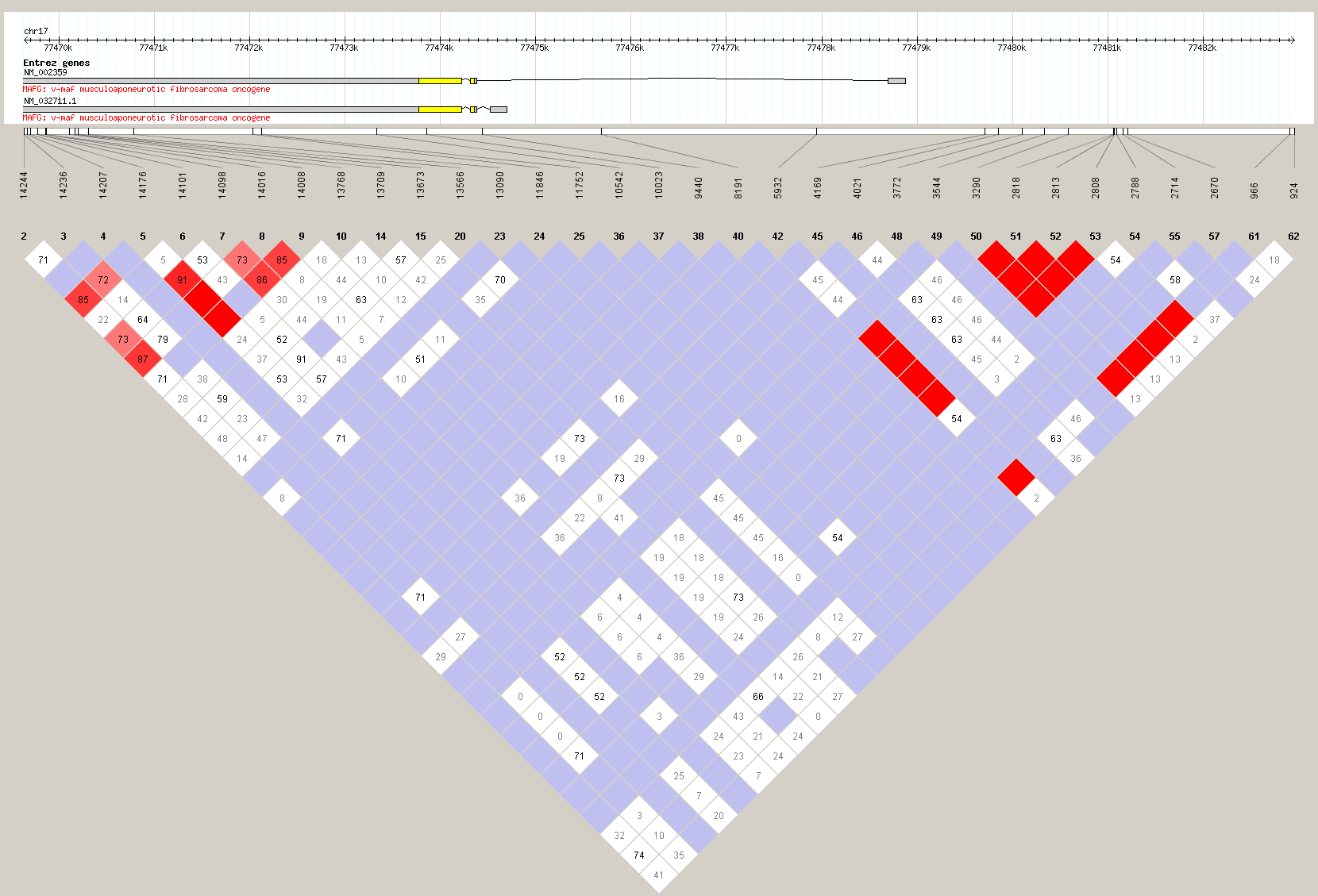

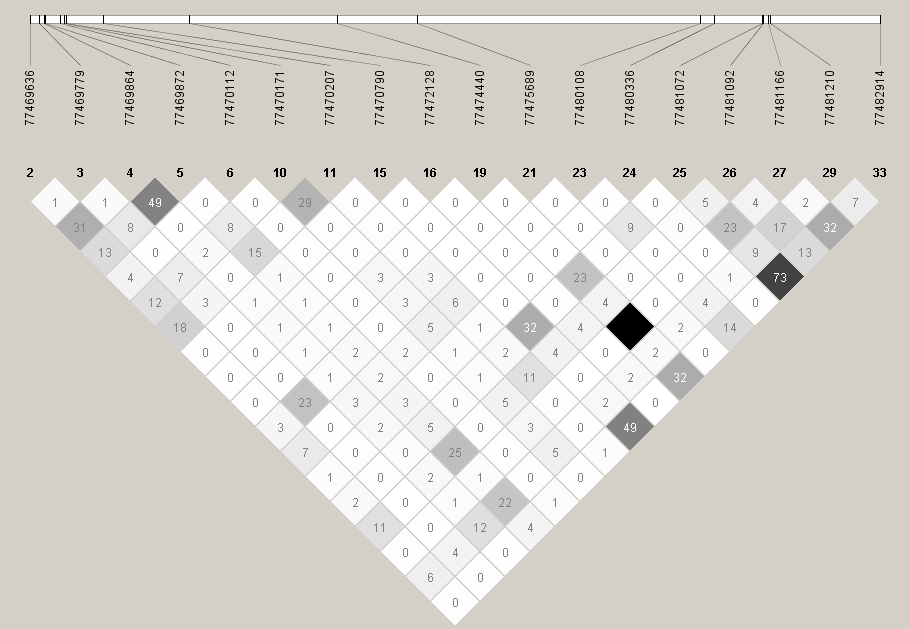


Figure S1B. The associations of MAFG SNPs with gene expression (black dots) or lung cancer status (pink dots). These SNPs were discovered by resequencing the MAFG locus. A novel MAFG SNP at chr17:77469864 in the 3’ UTR (indicated by red box and arrow) differed in frequency among SC and SNC (p_corrected = 0.0199), and was also marginally associated with the higher expression level of MAFG (p_corrected = 0.0596).
